# Supplementary material for: A humanized monoclonal antibody targeting an ectonucleotidase rescues cardiac metabolism and heart function after myocardial infarction
Source: Cell Rep Med. 2024 Oct 24;5(11):101795. doi: 10.1016/j.xcrm.2024.101795 (PMC11604407; doi:10.1016/j.xcrm.2024.101795)
Supplement: Document S1. Figures S1–S10 and Tables S1–S5 and S7–S9 [file mmc1.pdf]

Cell Reports Medicine, Volume *5*

Supplemental information

A humanized monoclonal antibody targeting an ectonucleotidase rescues cardiac metabolism and heart function after myocardial infarction

Shen Li, Bo Tao, Jijun Wan, Enca Montecino-Rodriguez, Ping Wang, Feiyang Ma, Baiming Sun, Yiqian Gu, Sivakumar Ramadoss, Lianjiu Su, Qihao Sun, Johanna Ten Hoeve, Linsey Stiles, Jeffrey Collins, R. Michael van Dam, Mikayla Tamboline, Richard Taschereau, Orian Shirihai, Douglas B. Kitchen, Matteo Pellegrini, Thomas Graeber, Kenneth Dorshkind, Shili Xu, and Arjun Deb

Supplementary Materials for

A Humanized Monoclonal Antibody Targeting an Ectonucleotidase Rescues Cardiac Metabolism and Heart Function after Myocardial Infarction

Shen Li[1,2,3,4,5,6], Bo Tao[1,2,3,4,5,6], Jijun Wan[1,2,3,4,5,6], Enca Montecino-Rodriguez[13], Ping Wang[1,2,3,4,5,6], Feiyang Ma[7], Baiming Sun[1,2,3,4,5,6],Yiqian Gu[3,4,5], Sivakumar Ramadoss[1,2,3,4,5,6], Lianjiu Su[1,2,3,4,5,6], Qihao Sun[1,2,3,4,5,6], Johanna Ten Hoeve[6,8,9,10], Linsey Stiles[6,11], Jeffrey Collins[6,9,10], R. Michael van Dam[6,9,10], Mikayla Tamboline[6,9,10], Richard Taschereau[6,9,10],Orian Shirihai[6,11], Douglas B. Kitchen[12], Matteo Pellegrini[3,4,5], Thomas Graeber[6,8,9,10], Kenneth Dorshkind[13], Shili Xu[6,9,10,14], Arjun Deb*[1,2,3,4,5,6]

*Address Correspondence to: adeb@mednet.ucla.edu

The following is included in the supplementary material.

Fig. S1 to S10
Tables S1-S9

Fig. S1

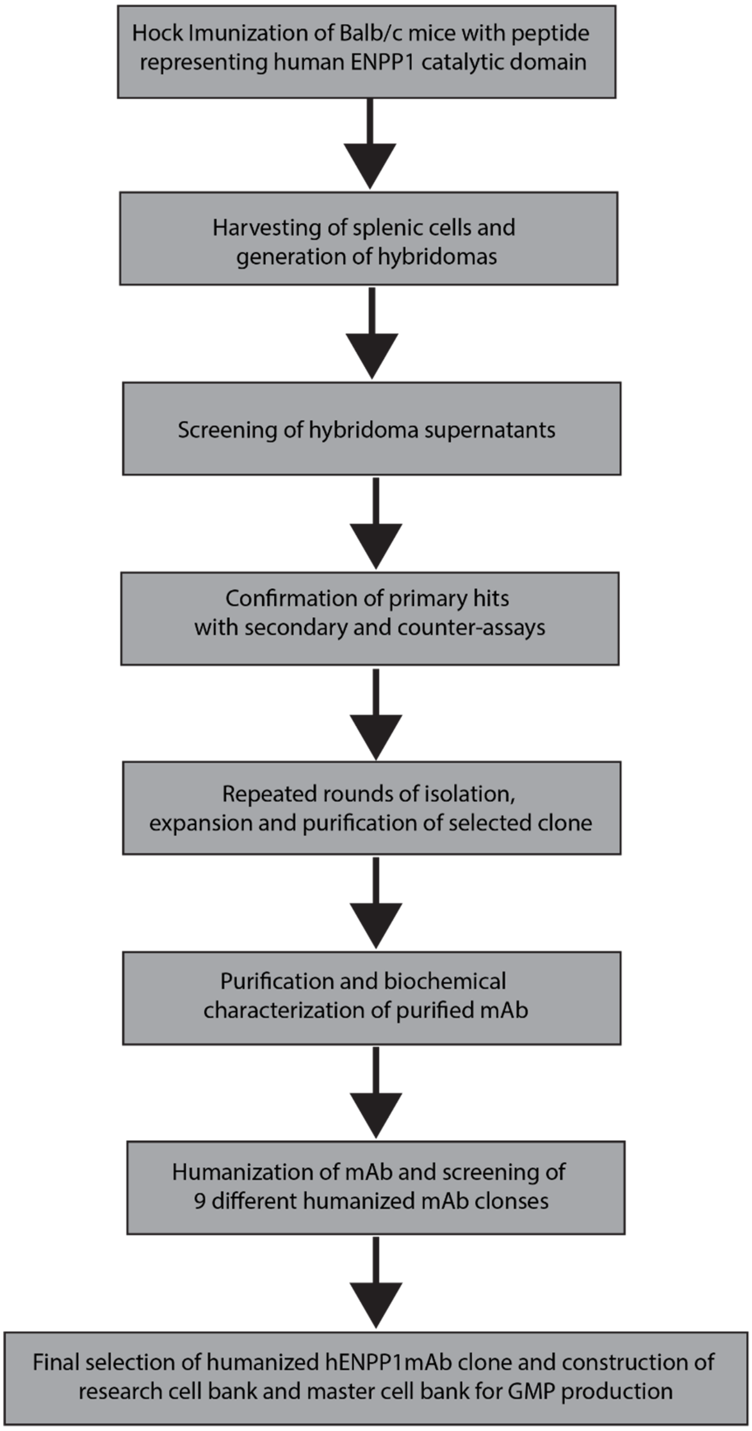

Fig S1. Schematic diagram of hENPP1mAb generation. Related to Figure 1. Schematic diagram of steps followed to generate a humanized ENPP1mAb. A peptide representing the human ENPP1 catalytic domain (generated in a mammalian cell line) was injected into Balb/c mice to immunize mice, followed by isolation of splenic cells and generation of hybridomas, screening of hybridoma supernatants and selection and expansion of clones, purification of mouse monoclonal antibody, followed by rounds of further cloning, and then full humanization of antibody, followed by screening of humanized clones and final selection of hENPP1mAb for research cell and master cell bank production.

Fig. S2

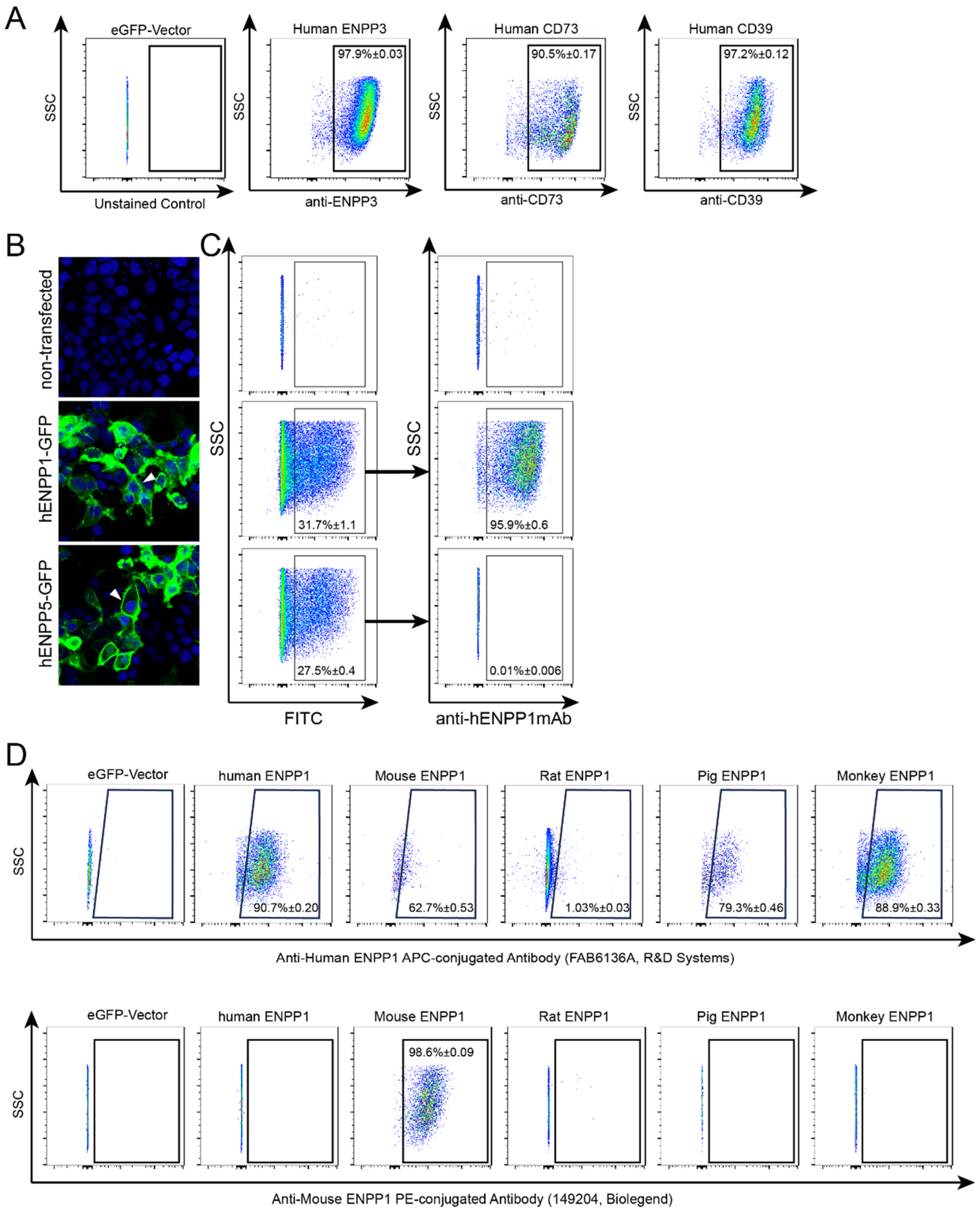
A
eGFP-Vector
Human ENPP3
Human CD73
Human CD39
97.9%±0.03
90.5%±0.17
97.2%±0.12
SSC
Unstained Control
anti-ENPP3
anti-CD73
anti-CD39
B
non-transfected
hENPP1-GFP
hENPP5-GFP
C
SSC
31.7%±1.1
27.5%±0.4
95.9%±0.6
0.01%±0.006
FITC
anti-hENPP1mAb
D
eGFP-Vector
human ENPP1
Mouse ENPP1
Rat ENPP1
Pig ENPP1
Monkey ENPP1
90.7%±0.20
62.7%±0.53
1.03%±0.03
79.3%±0.46
88.9%±0.33
Anti-Human ENPP1 APC-conjugated Antibody (FAB6136A, R&D Systems)
98.6%±0.09
Anti-Mouse ENPP1 PE-conjugated Antibody (149204, Biolegend)

Fig S2. Verification the overexpression of different targets in HEK cell line. Related to Figure 1 and 2. **(A)** Commercial anti-ENPP3, anti-Cd73, and anti-CD39 antibodies were used to detect corresponding antigens in HEK cells over-expressing them. (n=3). **(B)** hENPP1-GFP and hENPP5-GFP fusion proteins were expressed in HEK cells. Left panel demonstrates surface expression of fused hENPP1-GFP and hENPP5-GFP on cell surface (arrowheads) compared to non-transfected cells. **(C)** Flow cytometry to determine expression of GFP and binding to hENPP1mAb. FITC expression in hENPP1-GFP and hENPP5-GFP expressing HEK cells, with unstained cells used for gating. hENPP1mAb binds to hENPP1-GFP fused protein but does not bind to hENPP5-GFP. **(D)** Flow cytometry demonstrating binding of species specific ENPP1 to either commercially available anti-human ENPP1 antibody or anti-mouse ENPP1 antibody. Species specific ENPP1 was cloned and over-expressed in HEK cells (n=3) The anti-human antibody reacts against human, pig and monkey ENPP1. A mouse specific antibody is used to demonstrate mouse specific ENPP1 expression**.**

Fig S3

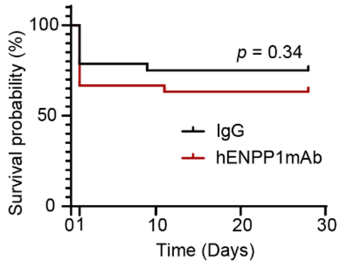

Fig S3. Kaplan Meier curves of survival after MI in the IgG and hENPP1mAb injected animals (n=28 in IgG, n=30 in hENPP1mAb). Related to Figure 3 and 4.

Fig. S4

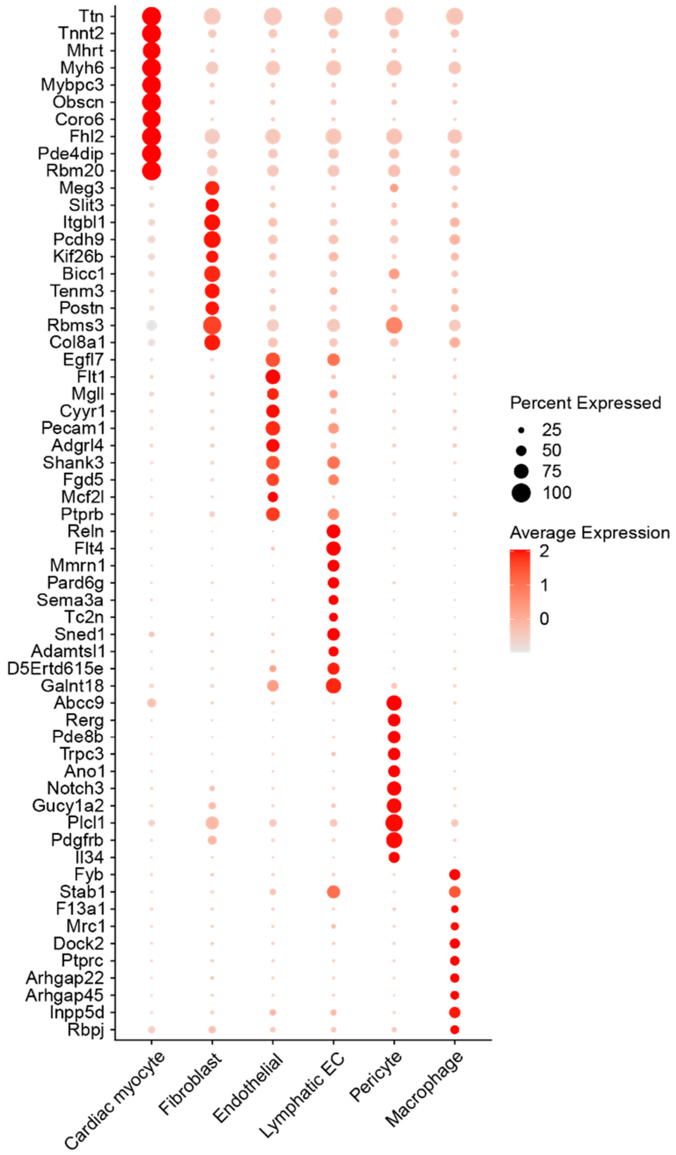

Fig S4. Single-nuclei RNA sequencing of hearts of IgG and hENPP1mAb treated animals at 7 days post MI. Related to Figure 5. Canonical genes used to characterize various cell population in the infarcted

Fig. S5

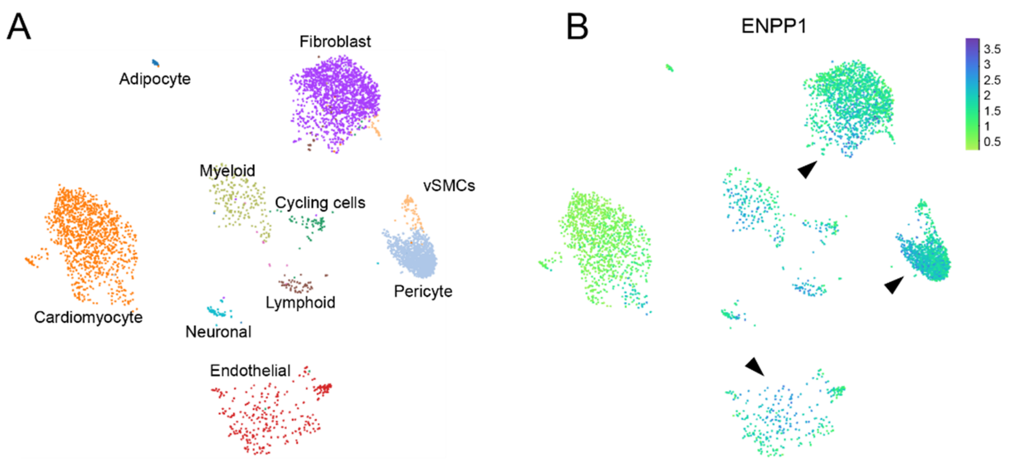

Fig S5. ENPP1 expression in different cell clusters in the infarcted human heart. Related to Figure 5. **(A)** Uniform manifold approximation and projection (UMAP) of snRNA-seq data of infarcted human heart samples. **(B)** Analysis of ENPP1 expression across cell populations. ENPP1 is observed to be expressed (arrowheads) predominantly in non-myocyte population of cardiac tissue in infarcted human hearts. (n=23 patients, Sampling time points, 2-166 days after infarction.) (ENPP1 expression data analyzed from published data sets in Kuppe, Christoph et al. "Spatial multi-omic map of human myocardial infarction." Nature Vol. 608,7924 (2022): 766-777. doi:10.1038/s41586-022-05060-x)

Fig. S6

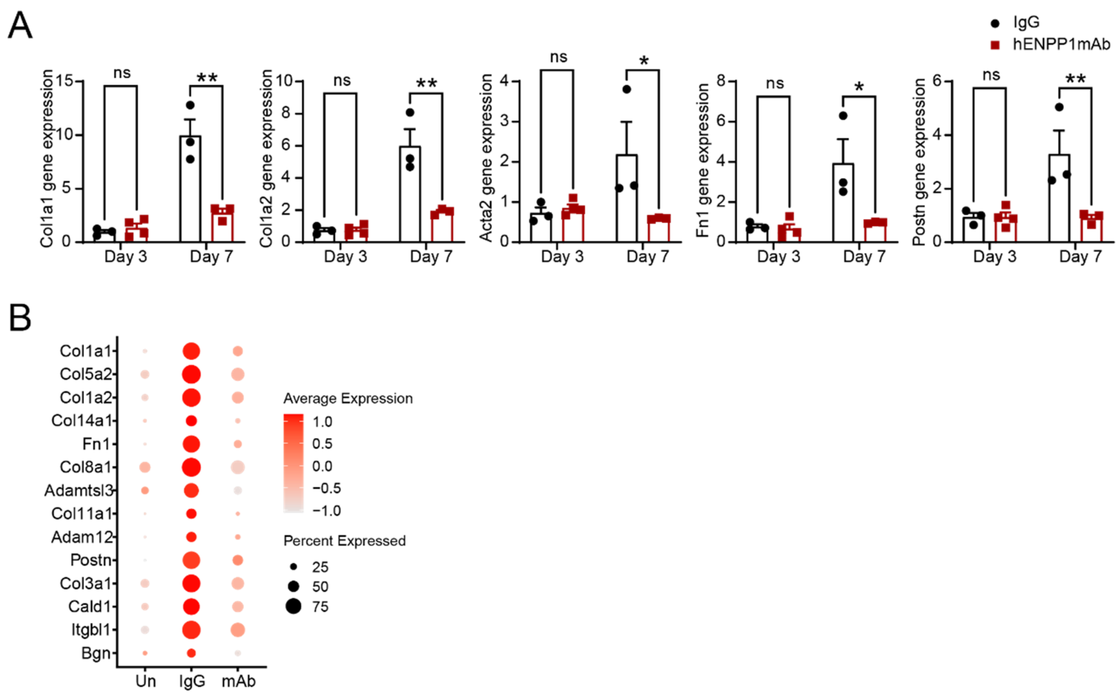

Fig S6. Extracellular matrix and myofibroblast gene expression in IgG and hENPP1mAb treated animals after MI. Related to Figure 5. (A) qPCR on injured region of hearts of IgG and hENPP1mAb treated animals at Day 3 and Day 7 following MI. (n=3 in Day 3 IgG, Day 7 IgG, and Day 7 hENPP1mAb; n=4 in Day 3 mAb). **(B)** Expression of ECM and myofibroblast genes in hearts of IgG and hENPP1mAb injected animals (Day 7 MI) compared to sham injured animals. Data represented as mean ± S.E.M., **p<0.01, *p<0.05, ns: not significant, Statistical significance was determined using Unpaired multiple t-test in **(A)**.

Fig. S7

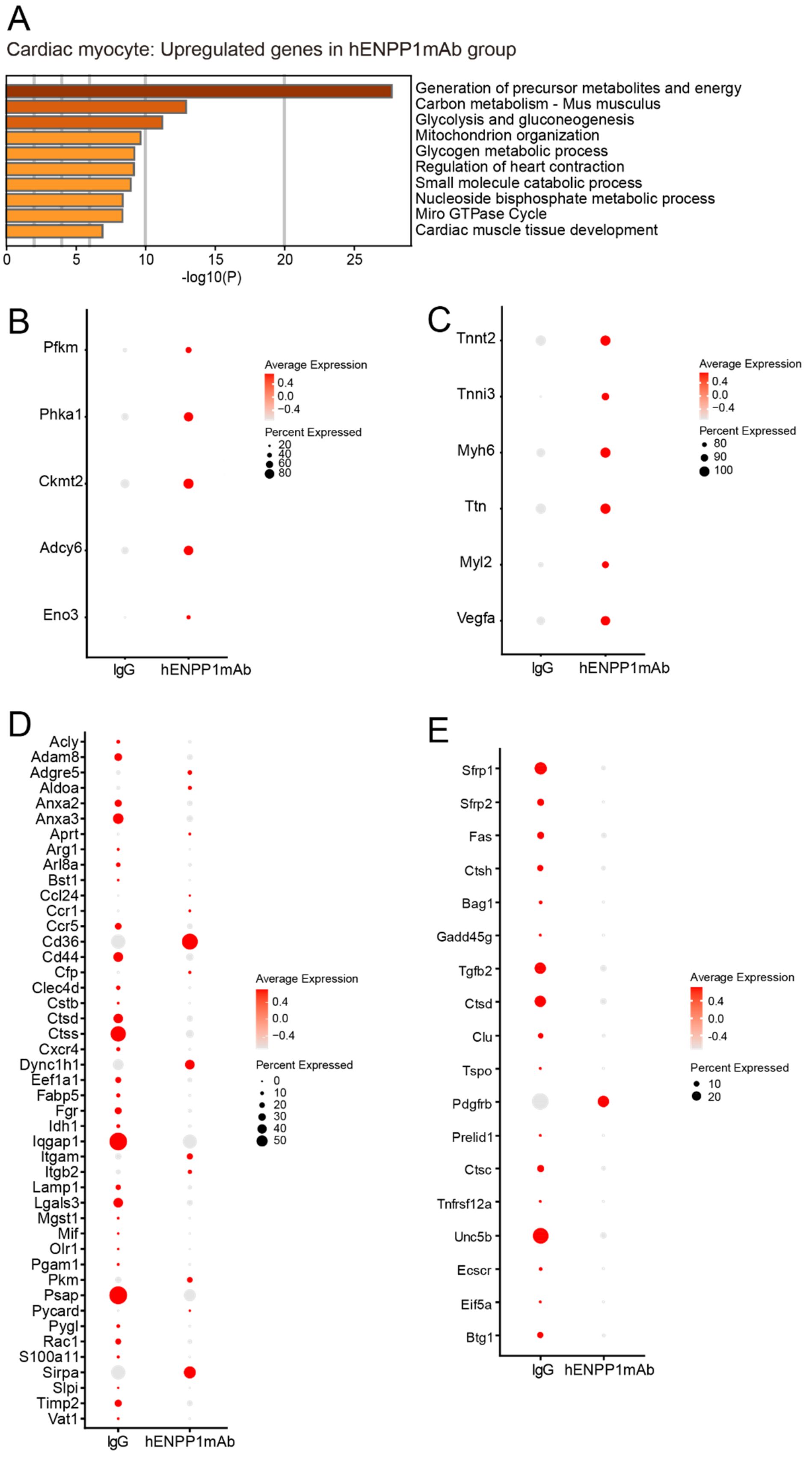

A
Cardiac myocyte: Upregulated genes in hENPP1mAb group
Generation of precursor metabolites and energy
Carbon metabolism - Mus musculus
Glycolysis and gluconeogenesis
Mitochondrion organization
Glycogen metabolic process
Regulation of heart contraction
Small molecule catabolic process
Nucleoside bisphosphate metabolic process
Miro GTPase Cycle
Cardiac muscle tissue development
0
5
10
15
20
25
-log10(P)
B
Pfkm
Phka1
Ckmt2
Adcy6
Eno3
IgG
hENPP1mAb
Average Expression
0.4
0.0
−0.4
Percent Expressed
20
40
60
80
C
Tnnt2
Tnni3
Myh6
Ttn
Myl2
Vegfa
IgG
hENPP1mAb
Average Expression
0.4
0.0
−0.4
Percent Expressed
80
90
100
D
Acly
Adam8
Adgre5
Aldoa
Anxa2
Anxa3
Aprt
Arg1
Arl8a
Bst1
Ccl24
Ccr1
Ccr5
Cd36
Cd44
Cfp
Clec4d
Cstb
Ctsd
Ctss
Cxcr4
Dync1h1
Eef1a1
Fabp5
Fgr
Idh1
Iqgap1
Itgam
Itgb2
Lamp1
Lgals3
Mgst1
Mif
Olr1
Pgam1
Pkm
Psap
Pycard
Pygl
Rac1
S100a11
Sirpa
Slpi
Timp2
Vat1
IgG
hENPP1mAb
Average Expression
0.4
0.0
−0.4
Percent Expressed
0
10
20
30
40
50
E
Sfrp1
Sfrp2
Fas
Ctsh
Bag1
Gadd45g
Tgfb2
Ctsd
Clu
Tspo
Pdgfrb
Prelid1
Ctsc
Tnfrsf12a
Unc5b
Ecscr
Eif5a
Btg1
IgG
hENPP1mAb
Average Expression
0.4
0.0
-0.4
Percent Expressed
10
20

Fig S7. Single Nuclei RNA-Seq demonstrating transcriptomic changes in myocytes and non-myocyte populations in hearts of IgG and hENPP1mAb treated animals at Day 7 post MI. Related to Figure 5. **(A)** GO analysis of main pathways differentially upregulated in cardiac myocytes in hENPP1mAb treated versus IgG control animals. **(B)** Dot plot demonstrating differential upregulation in myocytes of genes regulating glycolysis in hENPP1mAb injected animals. **(C)** Dot plot demonstrating differential upregulation of genes encoding for contractile proteins in hENPP1mAb injected animals. **(D)** Dot plot demonstrating the expression of inflammatory genes in macrophages of IgG versus hENPP1mAb injected animals. **(E)** Dot blot representing gene expression of pro-apoptotic genes across myocyte and non-myocyte cell populations of IgG versus hENPP1mAb injected animals. ($p<0.05$ for all genes shown differentially expressed, n=3/group for single nuclear transcriptomics)

Figure S8

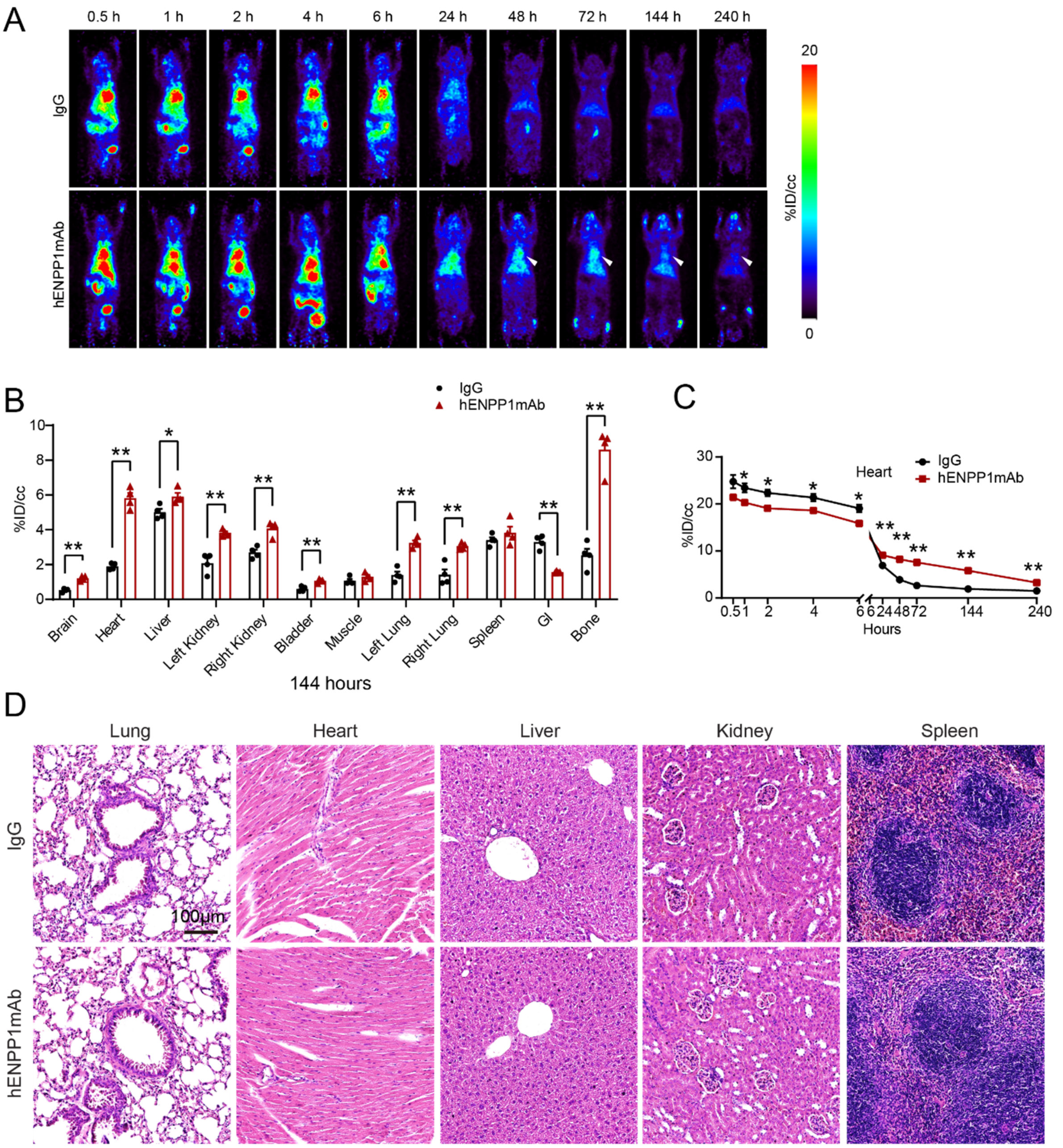

Fig S8. Organ wide biodistribution of radio-labeled hENPP1mAb after single dose administration in humanized ENPP1 animals. Related to Figure 2. (A) Representative longitudinal microPET imaging at 0.5, 1, 2, 4, 6, 24, 48, 72, 144, and 240 hours after Zr-IgG or 89Zr-hENPP1mAb injection in humanized ENPP1 mice. Arrowhead indicates the abundant 89Zr-hENPP1mAb signal in the heart at 48, 72, 144 and 240 hours (n=4 animals/group). **(B)** In vivo biodistribution of 89Zr-IgG or 89Zr-hENPP1mAb in brain, heart, liver, kidney, bladder, muscle, lung, spleen, gastrointestinal system and bone at 144 hours after injection (n=4 animals/group). **(C)** Time–activity (%ID/cc) curves in the hearts of 89Zr-IgG or 89Zr-hENPP1mAb treated animals. **(D)** Hematoxylin/eosin staining demonstrating normal histology of lung, heart, liver, kidney and spleen in both IgG (n=14) and hENPP1mAb injected animals (n=13). Data are represented as mean±SEM. $^{**}p<0.01$, $^{*}p<0.05$, Statistical significance was determined using Student's t-test, 2 tailed.

Fig. S9

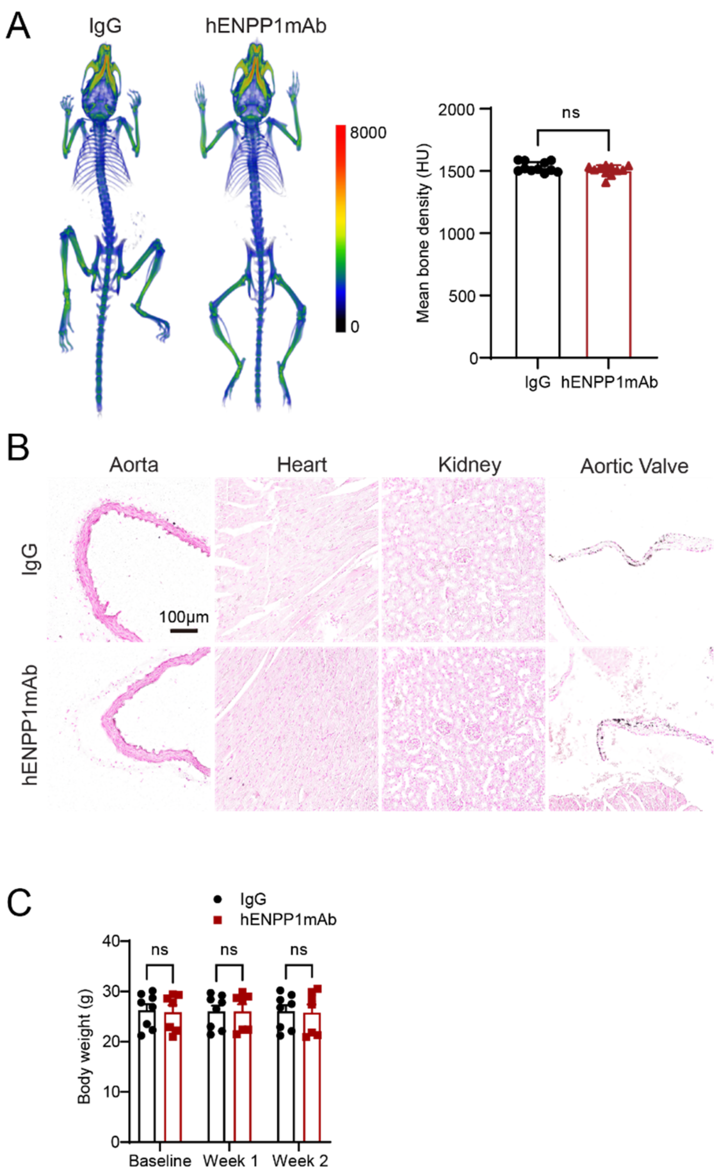

Fig S9. hENPP1mAb does not cause decreased bone mass or ectopic calcification. Related to Figure 2. (A) 3D rendering of bone density in IgG and hENPP1mAb treated humanized ENPP1 mice, 2 weeks after completion of 2 weeks of hENPP1mAb (i.e. 4 weeks after initial dose). Quantitative estimation of bone density (n=11 animals/group). The mean bone density (HU, Hounsfield unit) was calculated based on x-ray attenuation on microCT. **(B)** Von Kossa staining demonstrating absence of calcium deposits in aorta, heart, kidney and aortic valve in both IgG (n=14) and hENPP1mAb (n=13) treated mice. Organs were harvested at 4 weeks (i.e. 2 weeks after completion of 2 week treatment with hENPP1mAb). **(C)** Body weight at baseline, week 1 and week 2 following IgG control or hENPP1mAb treatment (n=8 in IgG and n=7 in hENPP1mAb). Data are represented as mean±SEM. ns: not significant, Statistical significance was determined using Student’s t-test, 2 tailed.

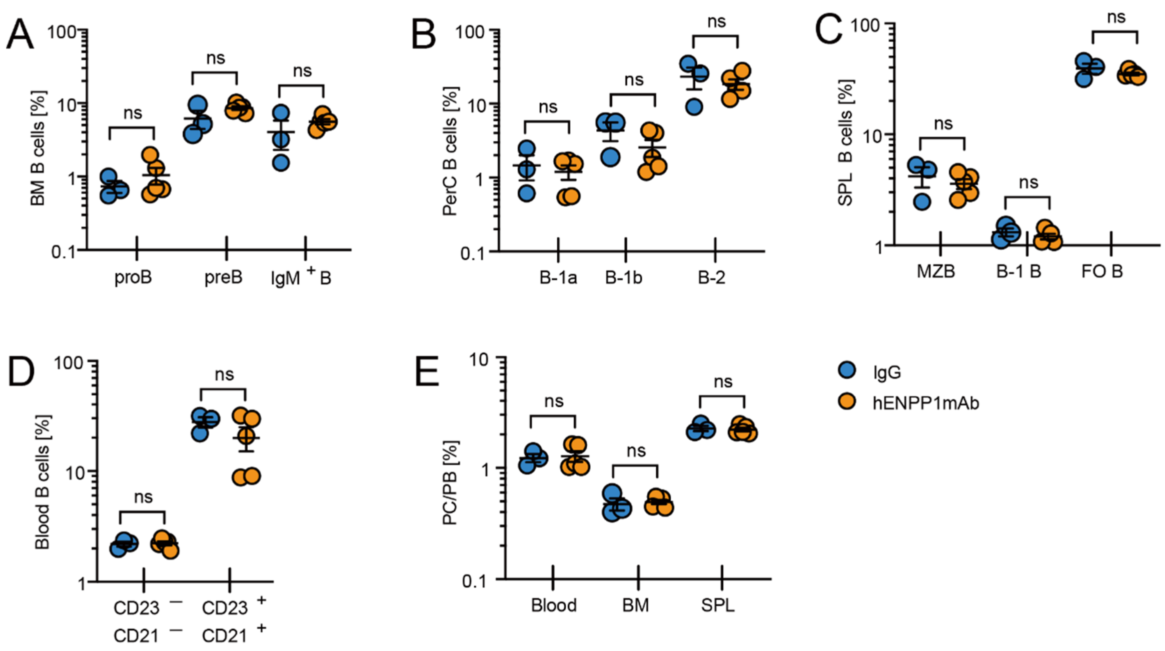

Fig S10. B cell populations between IgG treated and hENPP1mAb treated mice at 7 days following injection. Related to Figure 2. (A) Frequencies of B lineage cells in the bone marrow (BM) (proB, preB and IgM+ B cells). **(B)** Frequencies of B-1a, B-1b and B-2 cells in the peritoneal cavity (PerC). **(C)** Frequencies of Marginal Zone (MZB), B-1 and Follicular (FO) B-2 cells in the spleen (SPL). **(D)** Frequencies of CD23— CD21— IgM+ and CD23+ CD21+ IgM+ B cells in peripheral blood (Blood) and **(E)** Frequencies of plasma cells/plasma blasts (PC/PB) in peripheral blood (Blood), bone marrow (BM) and spleen (SPL). n=3 in IgG and n=5 in hENPP1mAb. Data represented as mean ± S.E.M., ns: not significant, Statistical significance was determined using Unpaired multiple t-test.

Table S1. Contingency table demonstrating number of animals with mild, moderate or severe impairment of ejection fraction at 4 weeks post MI, following treatment with hENPP1mAb versus IgG. Related to Figure 3.

	<20%	20%-40%	>40%	Total
IgG	11	10	0	21
hENPP1mAb	1	8	10	19

χ^2 =18.50, p value < 0.0001

Table S2. Contingency table demonstrating number of animals with mild, moderate or severe fibrosis at 4 weeks following MI in hENPP1mAb versus IgG treated groups. Related to Figure 4.

	<20%	20%-40%	>40%	Total
IgG	1	5	9	15
hENPP1mAb	7	12	3	22

χ^2 =9.39, p value =0.0091

Table S3. Serum biochemistry, complete blood counts (CBC) and differential blood counts in IgG and hENPP1mAb treated animals. Related to Figure 2. (p>0.05 for all values listed)

Biochemistry	IgG (n=14)	hENPP1mAb (n=13)
ALP (U/L)	86.14±29.046	83.77±26.671
AST (U/L)	280.5±218.169	387.46±206.731
ALT (U/L)	70.14±63.043	71.54±41.780
Albumin (g/dL)	3.03±0.338	3.09±0.161
Total Protein (g/dL)	4.89±0.475	5.05±0.161
BUN (mg/dL)	21.79±4.042	22.54±1.506
Creatinine (mg/dL)	0.10±0.104	0.11±0.104
Glucose (mg/dL)	209.79±55.445	217.85±27.850
Calcium (mg/dL)	8.59±0.818	8.43±0.523
Phosphorus (mg/dL)	8.21±1.959	8.48±0.939

CBC Parameters	IgG (n=14)	hENPP1mAb (n=13)
WBC (K/uL)	2.89±1.585	4.42±3.453
RBC (M/uL)	9.69±0.739	9.57±0.693
HGB (g/dL)	14.03±0.731	14.01±1.074
HCT (%)	44.87±3.334	44.5±3.062
MCV (fL)	46.29±0.994	46.46±1.127
Red Cell Distribution Width (%)	20.94±1.241	20.45±1.053
MCH (pg)	14.5±0.586	14.63±0.275
MCHC (g/dL)	31.31±1.275	31.47±0.705
Platelet Count (K/uL)	513.79±349.156	464.92±405.397

.

Differential (%)	IgG (n=14)	hENPP1mAb (n=13)
Neutrophil	10.78±5.353	9.12±5.937
Band (%)	none seen	none seen
Lymphocytes	81.11±5.945	82.36±7.209
Monocytes	4.6±2.134	6.23±3.164
Eosinpphils	3.24±2.632	2.17±1.770
Basophils	0.26±0.377	0.12±0.286
Metamyelocyte	none seen	none seen
Melocyte	none seen	none seen
Promyelocyte	none seen	none seen
Unclassified	none seen	none seen

Table S4. Serum biochemistry, CBC and Differential blood counts after 3 days of IgG and hENPP1mAb treatment. Related to Figure 2. ($p>0.05$ for all values listed)

Biochemistry	IgG (n=3)	hENPP1mAb (n=3)
ALP (U/L)	112.67±15.044	135.67±2.082
AST (U/L)	1041.50±919.946	828.67±250.698
ALT (U/L)	260.67±200.884	165.33±110.744
Albumin (g/dL)	2.97±0.231	2.93±0.321
Total Protein (g/dL)	4.77±0.416	4.73±0.586
BUN (mg/dL)	30.00±3.000	28.67±3.215
Creatinine (mg/dL)	0.10±0.100	0.10±0.100
Glucose (mg/dL)	242.00±20.664	245.00±27.875
Calcium (mg/dL)	8.47±2.146	9.00±0.265
Phosphorus (mg/dL)	10.17±0.987	9.37±2.281

CBC Parameters	IgG (n=3)	hENPP1mAb (n=3)
WBC (K/uL)	5.17±2.212	4.73±0.473
RBC (M/uL)	9.34±0.598	9.03±1.512
HGB (g/dL)	13.77±0.777	13.87±1.060
HCT (%)	42.47±3.584	39.70±8.390
MCV (fL)	45.67±1.528	44.00±2.646
Red Cell Distribution Width (%)	23.07±4.652	24.07±4.990
MCH (pg)	14.77±0.635	15.57±2.369
MCHC (g/dL)	32.53±2.747	35.97±7.941
Platelet Count (K/uL)	546.00±540.381	366.00±291.484

Differential (%)	IgG (n=3)	hENPP1mAb (n=3)
Neutrophil	7.83±4.537	9.77±8.177
Band (%)	0	0
Lymphocytes	87.33±6.658	84.77±11.927
Monocytes	4.07±2.101	4.10±2.152
Eosinophils	0.77±1.328	1.10±1.905
Basophils	0	0.27±0.462
Metamyelocyte	0	0
Melocyte	0	0
Promyelocyte	0	0
Unclassified	0	0

Body weight (g)	IgG (n=3)	hENPP1mAb (n=3)
Day 0	22.37±0.72	21.77±0.37
Day 3	21.43±0.39	21.83±0.47

Table S5. Elimination phase pharmacokinetic parameters of IgG and hENPP1mAb in Tg32 mice. Related to Figure 7.

	Half-Life (days)	Clearance (mL/days)	AUC (μg.days/mL)	Volume of Distribution (mL)
**IgG**	14.0±0.7	0.142±0.017	1274±161	2.85±0.32
**hENPP1mAb**	14.7±0.8	0.138±0.009	1210±36	2.93±0.24

Table S7. Sequences of cloning primers used. Related to STAR Methods.

	Forward Primer	Reverse Primer
human ENPP1	GCGGCCGCTGAGTTAACTATTCTAGAA<br>CCATGGACGTGGGGGAGGAGCCGCT	GGGGGGGGAGGGAGAGGGGCGGAT<br>CCTCAGTCTTCTTGGCTAAAGG
pig ENPP1	CGCTGAGTTAACTATTCTAGAACCATG<br>GAGCGCGACGGCTGTG	GGGGAGGGAGAGGGGCGGATCCTC<br>AGTCTTCTTGGTTAAAG
rat ENPP1	CGCTGAGTTAACTATTCTAGAACCATG<br>GAGCGCGACGGCGAACA	GGGGAGGGAGAGGGGCGGATCCTC<br>AGTCTTCTTGGCTGAAGAT
mouse ENPP1	GCGGCCGCTGAGTTAACTATTCTAGAA<br>CCATGGAGCGCGACGGCGA	GGGGGGGGAGGGAGAGGGGCGGAT<br>CCCTAGTCTTCTTGGCTGAAGATTG
monkey ENPP1<br>1st round upstream	ACGATGGAGCGCGACGGCT	AGGCAGCATCACAGCGACAGTTCC
monkey ENPP1<br>1st round<br>downstream	ATGGACGTGGGGGAGGAGC	GCTGAGTACGCAAGTTTCCCTGAGAT
monkey ENPP1<br>2nd round upstream	TATTCTAGAACCATGGAGCGCGACGGC<br>TG	AGGCAGCATCACAGCGACAGTTCC
monkey ENPP1<br>2nd round<br>downstream	TATATTCCATGGACGTGGGGGAGGAG<br>C	CTAGGATCCTCAGTCTTCTTGGCTAA<br>AGGTTGGCAAATGTG
human ENPP4	TATGCTAGCCACCATGAAGTTATTAGTA<br>ATACTTTTGTTTTCTGG	ATCCCGGGTCACCCAATTAAAGGATC<br>ATC
human ENPP5	TCCGCTAGCCACCATGACTTCGAAATT<br>TCTCTTGGTG	ATCCCGGGTTAGGCTTGTAATAATGG<br>TTGAGCT
human CD39	TCCGCTAGCCACCATGGAAAGTGAAGA<br>GTTGGCA	ATCCCGGGCTATACCATATCTTTCCA<br>GAAATATGAAG
human CD73	TATGCTAGCCACCATGTGTCCCCGAGC<br>CG	ATCCCGGGCTATTGGTATAAAACAAA<br>GATCACTGC
ENPP5-GFP	CCGGTCGCCACCATGGTGAGCAAGGG	CATGGTGGCGACCGGGGCTTGTAAT<br>AATGGTTGAGCT

Table S8. Sequences of ECM genes primers used. Related to STAR Methods.

	Forward primer	Reverse primer
Col1a1	TTCTCCTGGCAAAGACGGAC	CGGCCACCATCTTGAGACTT
Col1a2	CCCAGAGTGGAACAGCGATT	ATGAGTTCTTCGCTGGGGTG
Acta2	GTACCACCATGTACCCAGGC	GCTGGAAGGTAGACAGCGAA
Fn1	ATGAGAAGCCTGGATCCCCT	GAGAGCTTCCTGTCCTGTCT
Postn	TCCCTGATGTCATTACTGATCC	AACCATCTTCAGCCCTGAGC

Table S9. In vivo micro-CT imaging Parameters. Related to STAR Methods.

Acquisition Parameters	
x-ray tube voltage:	50 kVp
x-ray tube current:	200 μA
Exposure time:	20 ms
Number of projections:	21600
Scan duration:	12 mins
Capture rate:	1751 projections/min.
Camera pixels:	972 x 768
Pixel size:	149.6 μm

Reconstruction Parameters	
Method:	Iterative reconstruction (OSEM)
Number of subsets:	16
Number of iterations:	6
Voxel size:	0.108 mm isotropic
Volumetric image size:	380 x 380 x 200 voxels (thorax only)
Post-reconstruction filter:	Gaussian (spatial), Median (temporal)
