# Supplementary material for: A humanized monoclonal antibody targeting an ectonucleotidase rescues cardiac metabolism and heart function after myocardial infarction
Source: Cell Rep Med. 2024 Oct 24;5(11):101795. doi: 10.1016/j.xcrm.2024.101795 (PMC11604407; doi:10.1016/j.xcrm.2024.101795)
Supplement: Document S2. Article plus supplemental information [file mmc5.pdf]

Cell Reports Medicine

Article

A humanized monoclonal antibody targeting an ectonucleotidase rescues cardiac metabolism and heart function after myocardial infarction

Graphical abstract

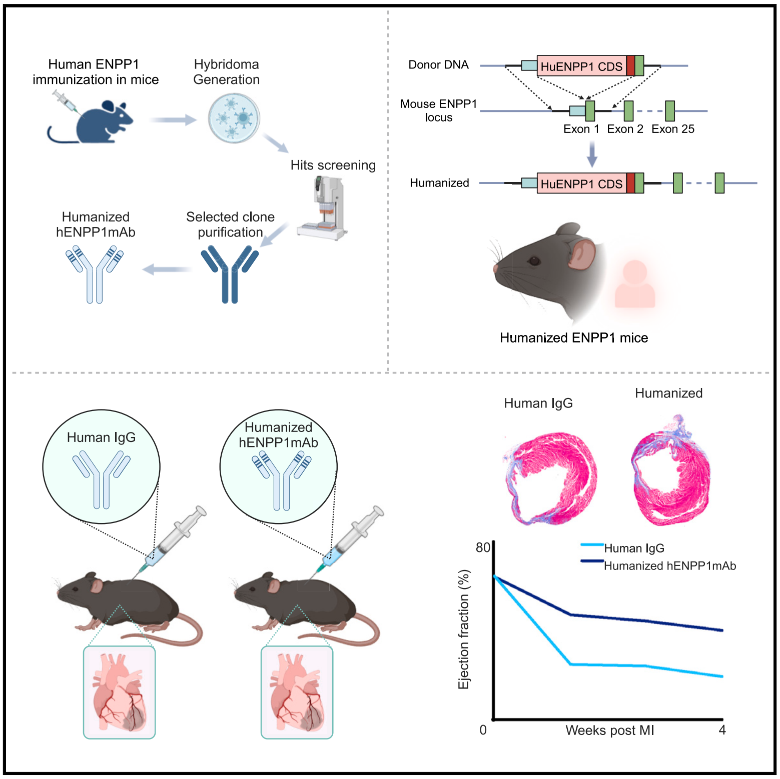

Highlights

- ENPP1 is known to initiate an aberrant metabolic cascade after myocardial infarction
- A humanized monoclonal antibody targeting human ENPP1 is engineered (hENPP1mAb)
- Systemic administration of hENPP1mAb rescues post-infarct metabolic defects
- In humanized mice, a single dose of hENPP1mAb rescues post-infarct heart function

Authors

Shen Li, Bo Tao, Jijun Wan, ..., Kenneth Dorshkind, Shili Xu, Arjun Deb

Correspondence

adeb@mednet.ucla.edu

In brief

After myocardial infarction, the ectonucleotidase ENPP1 initiates an aberrant metabolic cascade that disrupts nucleotide biosynthesis and worsens heart repair. Li et al. engineer a humanized monoclonal antibody targeting ENPP1 (hENPP1mAb) and demonstrate that a single dose of hENPP1mAb in humanized animals rescues metabolic defects and enhances post-infarct heart function.

Li et al., 2024, Cell Reports Medicine *5*, 101795
November 19, 2024 © 2024 The Author(s). Published by Elsevier Inc.
https://doi.org/10.1016/j.xcrm.2024.101795

CellPress

Cell Reports Medicine

CellPress
OPEN ACCESS

Article

A humanized monoclonal antibody targeting an ectonucleotidase rescues cardiac metabolism and heart function after myocardial infarction

Shen Li,[1,2,3,4,5,6] Bo Tao,[1,2,3,4,5,6] Jijun Wan,[1,2,3,4,5,6] Enca Montecino-Rodriguez,[13] Ping Wang,[1,2,3,4,5,6] Feiyang Ma,[7] Baiming Sun,[1,2,3,4,5,6] Yiqian Gu,[3,4,5] Sivakumar Ramadoss,[1,2,3,4,5,6] Lianjiu Su,[1,2,3,4,5,6] Qihao Sun,[1,2,3,4,5,6] Johanna Ten Hoeve,[6,8,9,10] Linsey Stiles,[6,11] Jeffrey Collins,[6,9,10] R. Michael van Dam,[6,9,10] Mikayla Tamboline,[6,9,10] Richard Taschereau,[6,9,10] Orian Shirihai,[6,11] Douglas B. Kitchen,[12] Matteo Pellegrini,[3,4,5] Thomas Graeber,[6,8,9,10] Kenneth Dorshkind,[13] Shili Xu,[6,9,10,14] and Arjun Deb[1,2,3,4,5,6,15,*]

[1]Division of Cardiology, Department of Medicine, University of California, Los Angeles, Los Angeles, CA, USA
[2]Cardiovascular Theme, David Geffen School of Medicine, University of California, Los Angeles, Los Angeles, CA, USA
[3]Department of Molecular, Cell & Developmental Biology, University of California, Los Angeles, Los Angeles, CA, USA
[4]Eli & Edythe Broad Center of Regenerative Medicine & Stem Cell Research, University of California, Los Angeles, Los Angeles, CA, USA
[5]Molecular Biology Institute, University of California, Los Angeles, Los Angeles, CA, USA
[6]California Nanosystems Institute, University of California, Los Angeles, Los Angeles, CA, USA
[7]Department of Cell and Developmental Biology, Feinberg School of Medicine, Northwestern University, Chicago, IL 60611, USA
[8]UCLA Metabolomics Center, University of California, Los Angeles, Los Angeles, CA, USA
[9]Crump Institute of Molecular Imaging, University of California, Los Angeles, Los Angeles, CA, USA
[10]Department of Molecular and Medical Pharmacology, University of California, Los Angeles, Los Angeles, CA, USA
[11]Department of Medicine, Endocrinology, David Geffen School of Medicine, University of California, Los Angeles, Los Angeles, CA 90095, USA
[12]Discovery Services, Curia Global, Inc., Albany, NY 12212, USA
[13]Department of Pathology and Laboratory Medicine, David Geffen School of Medicine at UCLA, Los Angeles, CA 90095, USA
[14]Jonsson Comprehensive Cancer Center, David Geffen School of Medicine, University of California, Los Angeles, Los Angeles, CA 90095, USA
[15]Lead contact
*Correspondence: adeb@mednet.ucla.edu
https://doi.org/10.1016/j.xcrm.2024.101795

SUMMARY

Myocardial infarction (MI) results in aberrant cardiac metabolism, but no therapeutics have been designed to target cardiac metabolism to enhance heart repair. We engineer a humanized monoclonal antibody against the ectonucleotidase ENPP1 (hENPP1mAb) that targets metabolic crosstalk in the infarcted heart. In mice expressing human ENPP1, systemic administration of hENPP1mAb metabolically reprograms myocytes and non-myocytes and leads to a significant rescue of post-MI heart dysfunction. Using metabolomics, single-nuclear transcriptomics, and cellular respiration studies, we show that the administration of the hENPP1-mAb induces organ-wide metabolic and transcriptional reprogramming of the heart that enhances myocyte cellular respiration and decreases cell death and fibrosis in the infarcted heart. Biodistribution and safety studies showed specific organ-wide distribution with the antibody being well tolerated. In humanized animals, with drug clearance kinetics similar to humans, we demonstrate that a single "shot" of the hENPP1mAb after MI is sufficient to rescue cardiac dysfunction.

INTRODUCTION

The adult mammalian heart has a limited ability to regenerate and heals via a fibrotic repair response after acute myocardial infarction (MI).[1] MI contributes to 40%–70% of all cases of incident heart failure, and the degree of cardiac fibrosis in failing hearts has been shown to be an independent prognostic indicator of survival and adverse cardiovascular events.[2,3] Modulation of cardiac wound healing to redirect the cardiac injury response from a fibrotic to a reparative remains a broad goal of cardiovascular therapeutics. Following MI, the cardiac microenvironment significantly changes with the recruitment of a diverse population of cells in a precise spatiotemporal manner,[4] enabling cellular crosstalk between myocytes and non-myocytes that is thought to affect various phases of wound healing.[5] Cell-cell crosstalk has been regarded as an attractive target to enhance cardiac repair as crosstalk affects multiple repair processes such as fibrosis, inflammation, angiogenesis, and cell metabolism.[5] Yet, there are no drugs available that enhance cardiac wound healing after acute MI.

© 2024 The Author(s). Published by Elsevier Inc.
This is an open access article under the CC BY license (http://creativecommons.org/licenses/by/4.0/).

We have recently described a metabolic crosstalk between myocytes and non-myocytes in the infarcted heart that affected cardiac repair.[6] Following ischemic cardiac injury, the extracellular ATP concentration increases from extravasation of intracellular ATP from injured myocytes.[7,8] We showed that an ectonucleotidase ENPP1 (ectonucleotide phosphodiesterase/pyrophosphatase 1) was significantly upregulated in the infarcted heart.[6] ENPP1 is a type II transmembrane protein that hydrolyzes extracellular ATP into AMP.[6,9] We showed that AMP generated by ENPP1 initiated a deleterious metabolic crosstalk between myocytes and non-myocytes that led to the formation of adenine and purine nucleosides and disrupted pyrimidine biosynthesis in cycling and non-cycling cells. A metabolic catastrophe caused by defects in pyrimidine biosynthesis resulted in functional defects in both myocytes and non-myocytes and worsened cardiac repair.[6] These observations suggest that ENPP1 could serve as a potential molecular target for augmenting cardiac repair. However, the efficacy of targeting human ENPP1 for cardiac repair remains unknown.

Here, we engineer a humanized monoclonal antibody targeting human ENPP1, create humanized ENPP1 mice that express the human instead of the murine ortholog, and demonstrate that administration of hENPP1mAb leads to transcriptional and metabolic rewiring of the infarcted heart and leads to superior post-infarct heart function. In animals genetically engineered to express antibody clearance kinetics similar to that in humans, we demonstrate that a single dose of humanized hENPP1mAb is sufficient to enhance post-infarct cardiac repair with significantly better preservation of post-infarct heart function.

RESULTS

Generation of a humanized monoclonal antibody against human ENPP1 that is potent and highly specific

We initially generated a monoclonal antibody targeting the extracellular catalytic domain of human ENPP1. For this purpose, a peptide of 840 amino acids that represented the catalytic domain of human ENPP1, and synthesized in a mammalian cell line, was injected into wild-type BALB/c mice followed by harvesting of splenic cells, generation of hybridomas, screening of hybridoma supernatants, and selection of the appropriate clone (Figure S1). The selected monoclonal was subsequently humanized for development as a clinical candidate, where the complementarity-determining regions (CDRs) of the antibody were grafted onto a human antibody scaffold,[10] and the degree of "humanness" of the humanized antibody was scored with proprietary software. The final humanized ENPP1 monoclonal antibody (hENPP1mAb) was thereafter synthesized in a recombinant manner in a stable mammalian cell line (Figure S1).

We overexpressed human ENPP1 in human embryonal kidney (HEK) cells and using flow cytometry observed the dissociation constant (K_D, that provides a measure of avidity) of hENPP1mAb to be 0.08 nM (Figure 1A). A luciferase-based cell-free assay to determine the potency of hENPP1mAb to inhibit human ENPP1 catalytic activity demonstrated an IC_{50} of 1.3 nM (Figure 1B). As the crystal structure of ENPP1 has been described,[11,12] we next performed homology modeling and protein-protein docking interactions using computational tools, and hENPP1mAb was predicted to bind near the active site of human ENPP1 (Figure 1C). All the best-scoring models on protein-protein docking demonstrated that hENPP1mAb significantly blocked access to the enzyme site, and portions of the CDR L-2 and CDR H-3 were predicted to insert into the active catalytic site of human ENPP1 (Figure 1D). We next examined the binding of hENPP1-mAb to a variety of related human ectonucleotidases and other human proteins. As members of the ENPP family such as ENPP3, ENPP4, and ENPP5 have been shown to possess ectonucleotidase activity,[13] we cloned and overexpressed these genes in HEK cells, but hENPP1mAb did not bind to other members of the human ENPP family on flow cytometry, demonstrating binding specificity only to human ENPP1 (Figure 1E). hENPP1mAb also did not bind to other ectonucleotidases active in the heart such as CD39 or CD73, but ENPP3, CD39, and CD73 antigens were recognized by antibodies specific to those antigens (Figure S2A). As there are no antigen-specific antibodies for ENPP4/5 for flow cytometry, to demonstrate membrane expression, we created ENPP-GFP fusion constructs and demonstrate successful expression of hENPP5 on the cell surface but lack of binding to hENPP1mAb on flow cytometry (Figures S2B and S2C). As a positive control, with flow cytometry, hENPP1mAb recognized hENPP1-GFP fusion constructs expressed on the cell surface demonstrating the specificity of hENPP1mAb for hENPP1 (Figures S2B and S2C) To determine off-target binding to any human protein, we utilized a membrane array system where more than 6,000 secreted and membrane-bound human proteins are expressed on the cell surface of HEK cells.[14,15] An immunoassay is then performed and scored to determine the binding of hENPP1mAb to each of the greater than 6,000 human proteins on the array. Using this assay, with appropriate positive and negative controls, we observed that hENPP1mAb only binds to the secreted and membrane-bound forms of hENPP1 and does not demonstrate specific binding to any other human protein (Figures 1F–1H).

Generation of a humanized ENPP1 animal for determining *in vivo* efficacy of hENPP1mAb

As the ENPP1mAb was initially isolated from mice following injection of the human ENPP1 peptide fragment, we next determined species reactivity of hENPP1mAb against ENPP1 orthologs of different species. For this purpose, we cloned the ENPP1cDNA of mouse, rat, pig, monkey, and human, overexpressed them in HEK cell lines, and determined binding of the hENPP1mAb to ENPP1 orthologs of different species by flow cytometry (Figure 2A). We observed that hENPP1mAb exhibited binding to human and monkey ENPP1 but not to ENPP1 orthologs of other species tested (Figures 2A and S2C).

As the hENPP1mAb did not exhibit binding to ENPP1 orthologs of lower species, and to test the efficacy of the hENPP1mAb *in vivo*, we genetically engineered a mouse expressing human ENPP1 and not the mouse ortholog (humanized ENPP1 mouse). To generate the humanized ENPP1 mouse, we used CRISPR-Cas9 genome editing to knock in the hENPP1CDS into the murine ENPP1 gene loci (first exon). A polyadenylation signal was included in the knockin cassette (downstream of hENPP1 CDS) to prevent murine ENPP1 gene transcription (Figure 2B). Using discriminatory primers, we observed that hearts of

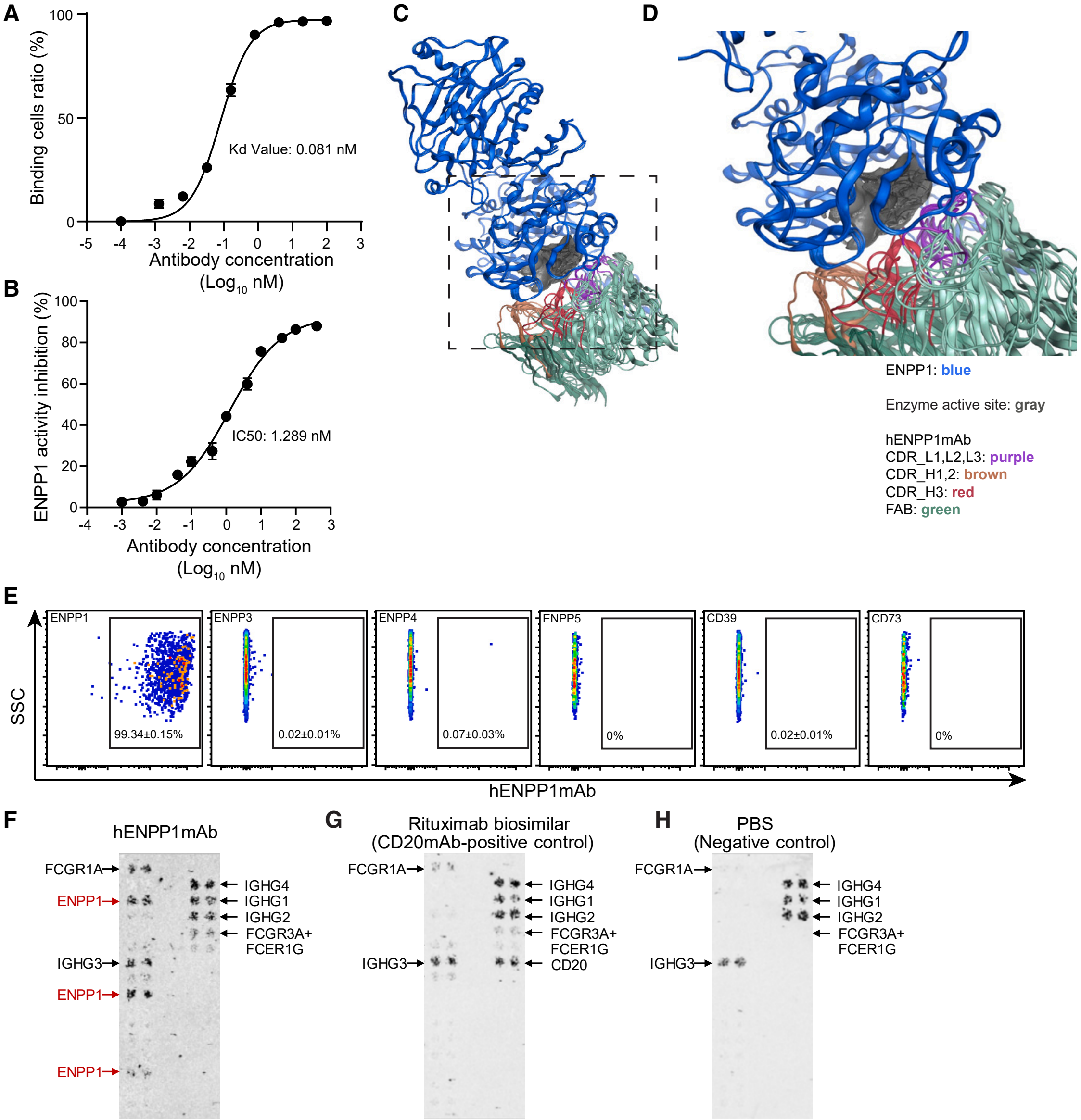

Figure 1. Avidity, potency, homology modeling, and off-target binding assays for hENPP1mAb

(A) Dissociation constant of hENPP1mAb calculated with flow cytometry to determine concentration-dependent binding of hENPP1mAb to HEK cells over-expressing human ENPP1. Calculated K_D value (measure of avidity) shown (n = 3).

(B) Potency curve of hENPP1mAb in inhibiting human ENPP1 catalytic activity in a concentration-dependent manner. Calculated IC50 shown (n = 3).

(C and D) Homology modeling and protein-protein docking interactions show the (C) docked model of hENPP1mAb and human ENPP1. The antibody chains (CDR of light and heavy chains) occlude the enzyme active site (dark gray surface) (human ENPP1 antigen in blue, Fab segment of hENPP1mAb in green, CDRs color coded as shown). (D) Magnified image demonstrating several CDRs of hENPP1mAb inserting into human ENPP1 catalytic domain.

(E) Flow cytometry to determine binding of hENPP1mAb to other members of human ENPP family and to other phosphatases (n = 5/group).

(F–H) Retrogenix membrane array screening by immunoblotting to determine binding of hENPP1mAb to 6,101 human plasma membrane proteins and 396 human heterodimers expressed on HEK cells.

(F) hENPP1mAb shows a significant specific interaction with human ENPP1 isoforms (red) (plasma membrane isoforms upper and middle, and tethered secreted form, lower). Note hENPP1mAb also shows binding to IGHG that may serve as IgG receptors.

(G) Rituximab is used as a positive control to determine any non-specific binding of a mAb and demonstrates no binding to human ENPP1 and binding to IGHG proteins.

(H) PBS is used as a negative control for the entire assay and demonstrates signal against the IGHG proteins as well. Immunoblotting demonstrates representative images of n = 3. Data are represented as mean ± SEM.

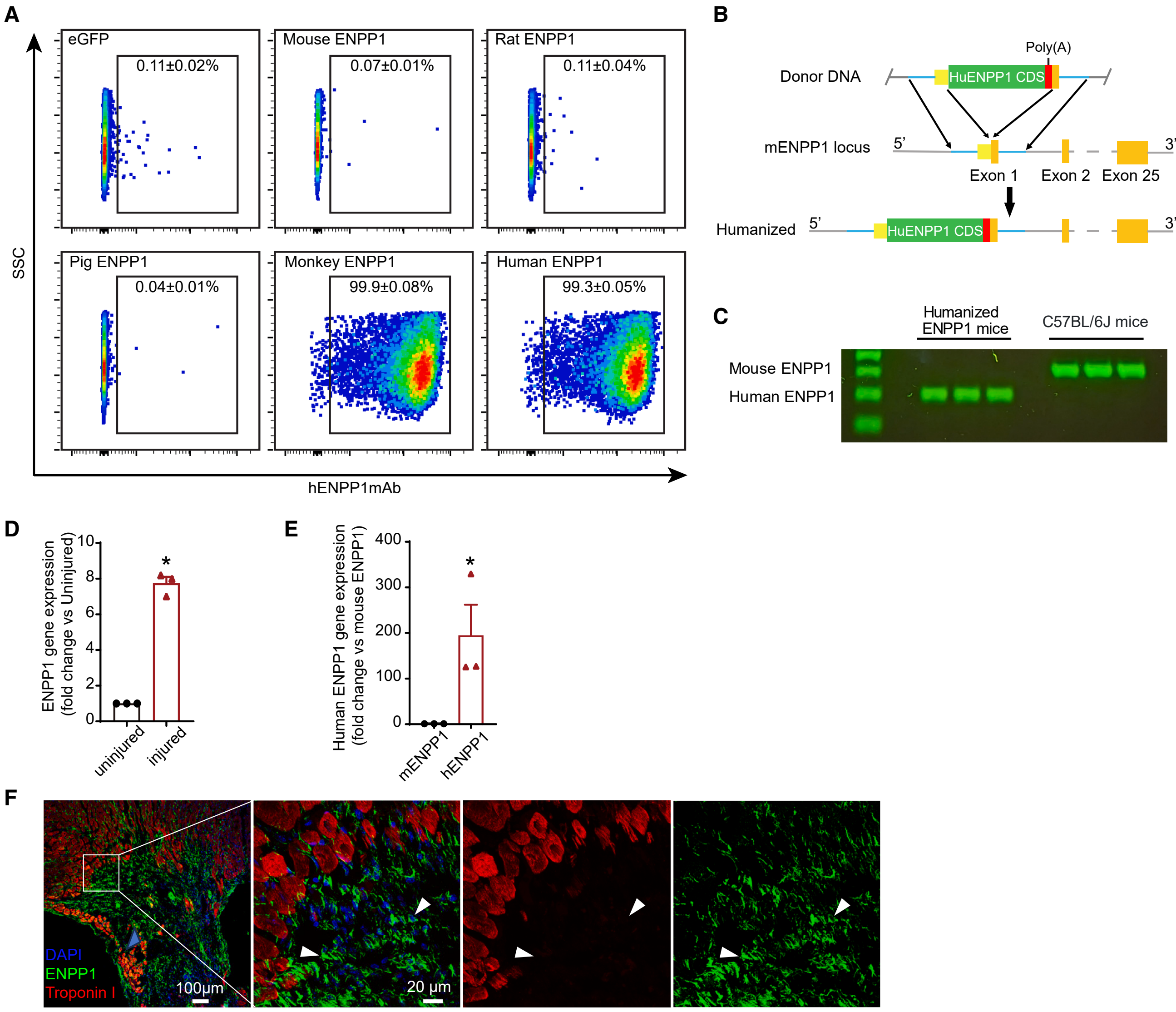

Figure 2. Species reactivity of hENPP1mAb and development of humanized ENPP1 mice

(A) Flow cytometry to determine binding of hENPP1mAb against mouse, rat, pig, monkey, and human ENPP1 overexpressed in HEK cell line. HEK cells expressing eGFP used as a negative control (*n* = 3 for eGFP and monkey, *n* = 5 in mouse, rat, pig, and human).

(B) Schematic representation of generation of the humanized ENPP1 mouse. Using CRISPR-Cas9, human ENPP1CDS with a PolyA signal at the 3′ end is inserted to replace the 1st exon of murine ENPP1 gene.

(C) Agarose gel electrophoresis of RT-PCR products of heart tissue from humanized ENPP1 mice or wild-type C57BL/6J mice (*n* = 3 animals/group). Discriminatory PCR primers are used to distinguish murine and human ENPP1 expression.

(D) qPCR demonstrating ENPP1 gene expression in the injured region of the heart compared with uninjured region at 7 days after MI (*n* = 3 animals/group).

(E) qPCR on infarcted heart of humanized ENPP1 mouse at day 7 post MI demonstrating the absence of murine ENPP1 and expression of human ENPP1 in the infarcted region (*n* = 3 animals/group).

(F) Immunostaining for ENPP1 (green, arrowheads) and cardiac troponin I (red) in the injured regions at day 7 after MI. Magnified images demonstrate cells in the infarcted region of the inset expressing human ENPP1 (arrowheads). Note that ENPP1 expression is present in troponin-negative regions. Data are represented as mean ± SEM. $^*p < 0.05$, Statistical significance was determined using Student's t test, 2 tailed.

humanized ENPP1 animals expressed human but not murine ENPP1 (Figure 2C). Next, we subjected the humanized ENPP1 animal to MI via ligation of the left anterior descending coronary artery and observed by qPCR that the expression of ENPP1 gene increased significantly in the infarcted area (Figure 2D) similar to what we had observed in post-MI wild-type animals. The infarcted heart of the humanized ENPP1 animals demonstrated increased gene expression of human but not mouse ENPP1 (Figure 2E) suggesting that regulatory elements driving post-injury ENPP1 expression were not disrupted in the humanized ENPP1 mouse. Immunostaining of the infarcted region demonstrated upregulated ENPP1 protein expression predominantly

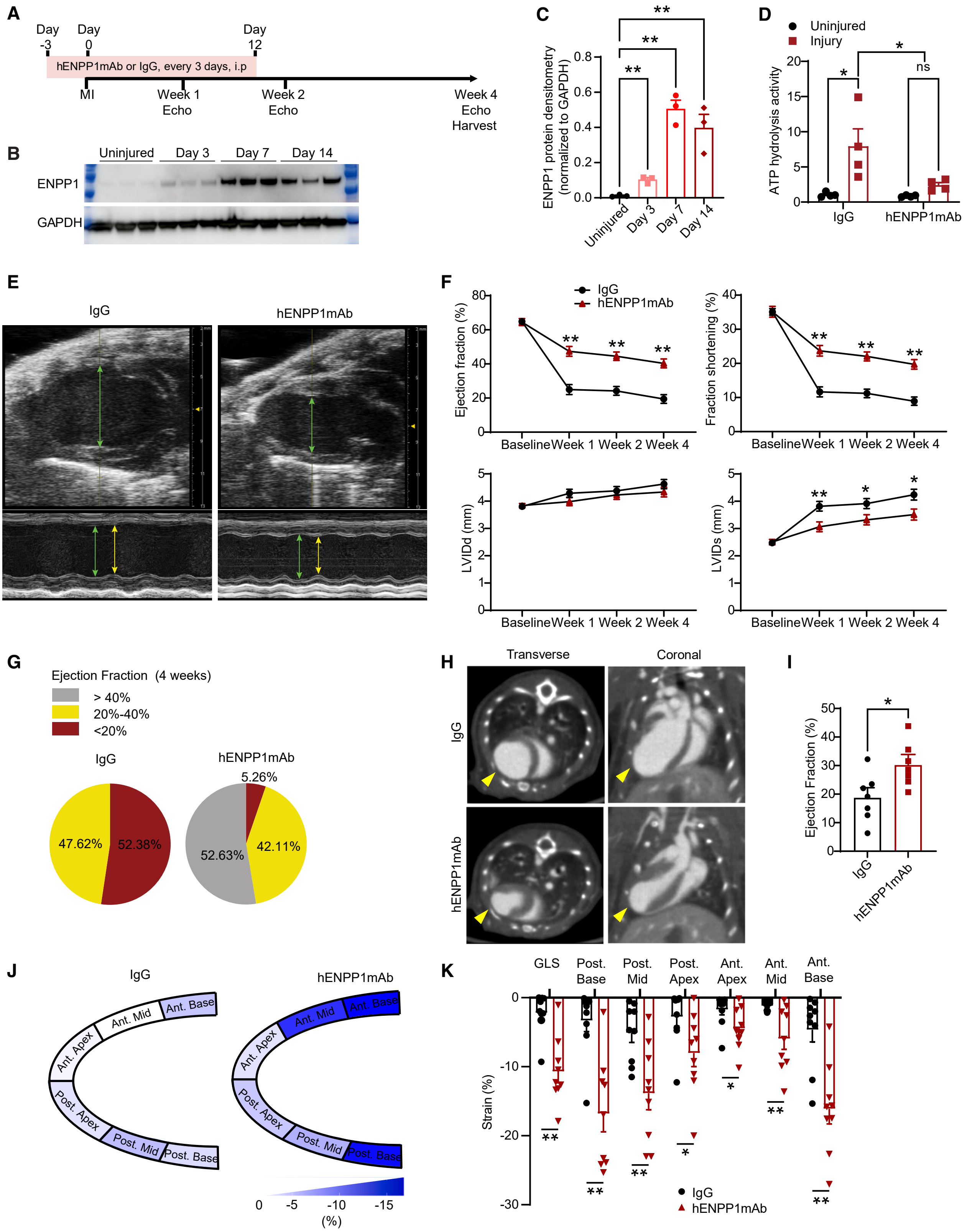

(legend on next page)

in the region of the scar (Figure 2F). The humanized ENPP1 mouse thus recapitulates ENPP1 expression in the wild-type mouse after MI and provides a platform to test the efficacy of hENPP1mAb for MI.

hENPP1mAb protects animals against MI-induced cardiac dysfunction

We subjected the humanized ENPP1 mouse to MI. The hENPP1-mAb has an IgG1 backbone and, in contrast to humans, the half-life of an IgG antibody is in the order of a few days in mice and thus necessitates repeated administration.[16] We injected the hENPP1mAb, 10 mg/kg, i.p. (intra-peritoneally) twice weekly (every 3 days) and administered the first dose 3 days prior to injury followed by repeated i.p. administration every 3 days for 12 days post injury (Figure 3A). Control animals were injected with human IgG in an identical manner and concentration (Figure 3A). Western blotting on whole heart lysates demonstrated that ENPP1 protein expression in the heart increases robustly within 7 days of MI (Figures 3B and 3C). To confirm that the hENPP1mAb inhibits ENPP1 nucleotidase activity *in vivo*, we first isolated infarcted hearts of humanized ENPP1 animals at 7 days post MI. We measured ATP hydrolytic activity and observed that in IgG-injected animals, injury induced an increase in ATP hydrolytic activity consistent with increased ENPP1 activity, but in hENPP1mAb-injected animals, there was no increase in ectonucleotidase activity after injury (Figure 3D). Animals were subjected to weekly B and M mode echocardiography, and we observed that by day 7 post MI, ejection fraction (EF) and rractional shortening (FS) in hENPP1mAb-injected animals were almost double that of IgG-injected animals (EF 47.34% ± 2.86% in hENPP1mAb versus 24.95% ± 2.95% in IgG group, and FS 23.70% ± 1.55% in hENPP1mAb versus 11.66% ± 1.48% in IgG groups, $p < 0.01$) (Figures 3E and 3F). The end systolic dimension of the ventricle (LVIDs) was also significantly decreased in the hENPP1mAb-injected animals compared to IgG-injected controls, demonstrating decreased post-infarct ventricular dilatation in hENPP1mAb-injected animals (Figures 3E and 3F). The functional benefits persisted at 4 weeks approximately 2 weeks after completion of the last dose of hENPP1mAb administration (Figure 3F). To determine the effects of the hENPP1mAb on post-infarct heart failure, we stratified the post-infarct EF (week 4) as severe heart failure (EF<20%), moderate heart failure (EF: 20%–40%), and mild heart failure (EF>40%). The fraction of animals that developed severe heart failure at 4 weeks in the IgG-injected group was 52% but only 5% of the animals in the hENPP1mAb-injected group developed severe heart failure demonstrating an order of magnitude improvement in the severity of post-infarct heart failure with hENPP1mAb therapy (Figure 3G; Table S1). Analysis of survival curves also did not show any significant difference between the IgG and the hENPP1mAb-injected animals (Figure S3). To examine the scar size and cardiac remodeling in greater detail, we next performed contrast-enhanced gated cardiac computed tomography (CT) scans of live animals at 14 days following hENPP1mAb or IgG injection. Transverse cuts on gated cardiac CT demonstrated a far more dilated left ventricle in the IgG-injected animals, and coronal slices demonstrated a smaller anterior wall scar with thicker ventricular walls in the hENPP1mAb-injected animals (Figure 3H). Consistent with echocardiography, EF estimated by gated cardiac CT imaging was also significantly higher in the hENPP1mAb-injected animals (Figure 3I), and robust vigorous contraction of the left ventricular walls was noted on gated cardiac CT imaging in the hENPP1mAb-injected animals (Videos S1 and S2). We next performed strain imaging to determine the contractile forces (wall strain) generated by various segments of the infarcted mouse heart in hENPP1mAb versus IgG-injected animals.[17] Strain imaging simply represents the deformation of the cardiac muscle in systole and can be represented as $\Delta L/L_0$,[18] where ΔL represents the change of the muscle fiber/segment in length at the end of systole and L_0 is the initial muscle fiber length prior to onset of systole (as the length is shortening during systole, strain numbers are usually represented as negative in value). The segments of the heart were divided from base to apex and posterior to anterior (Figure 3J). We observed that the wall strain (contractile force) was significantly greater (deeper color on heatmap) across all segments of hearts of animals that received hENPP1mAb (Figure 3J). Quantification of strain forces demonstrated several fold higher strain forces generated in the myocardial segments of animals that received hENPP1mAb compared to IgG (Figure 3K).

Figure 3. hENPP1mAb attenuates post-infarct cardiac dysfunction in humanized ENPP1 animals

(A) Strategy for hENPP1mAb administration in humanized ENPP1 animals subjected to MI.
(B) Western blotting for ENPP1 in wild-type mice hearts at 3, 7, and 14 days following MI.
(C) Quantitative densitometry of ENPP1 level ($n = 3$).
(D) Extracellular ATP hydrolytic activity in injured and uninjured hearts of animals treated with IgG or hENPP1mAb ($n = 4$ animals/group).
(E) B (top) and M-mode (below) echocardiogram demonstrating superior contractile function in hENPP1mAb-treated animals. Diastolic (green line) and systolic internal dimensions (yellow line) in hearts of hENPP1mAb/IgG-treated animals.
(F) Ejection fraction, fractional shortening, and left ventricular (LV) chamber size in systole (LVIDs) and diastole (LVIDd) in IgG or hENPP1mAb-treated animals at 1, 2, and 4 weeks following MI ($n = 21$/IgG and $n = 19$/hENPP1mAb).
(G) Pie chart illustrating the fraction of animals with mild, moderate, and severe reduction in EF at 4 weeks after injury following IgG or hENPP1mAb administration.
(H) 4D gated cardiac CT showing transverse and coronal views of the heart of IgG or hENPP1mAb-injected animals at day 14 post MI (arrowheads point to the thin wall post infarct scar that is decreased in hENPP1mAb-injected groups).
(I) Ejection fraction measurement by gated cardiac CT ($n = 7$ animals/group).
(J) Myocardial strain analysis of cardiac segments in longitudinal axis at day 7 post MI in IgG versus hENPP1mAb-treated animals. Heatmap demonstrating wall strain generated with deeper color corresponding to greater contractile force.
(K) Myocardial deformation measurements to demonstrate strain forces generated at various cardiac segments between IgG and hENPP1mAb-treated animals. GLS, global longitudinal strain); Post, posterior base; Post. Mid; Post. Apex; Ant., Anterior apex; Ant. Mid and Ant. base. ($n = 9$ animals/group). Data are expressed as mean ± SEM. $^{**}p < 0.01$, $^{*}p < 0.05$, ns: not significant. Statistical significance was determined using ordinary one-way ANOVA with Tukey's multiple comparison test (C and D), unpaired multiple t test (F), or Student's t test, 2 tailed (I and K).

hENPP1mAb administration is associated with histologic evidence of superior post-infarct repair

We next examined histological correlates of superior post-infarct heart function. The degree of post-MI fibrosis is known to be an independent prognostic factor regulating cardiovascular outcomes after MI with higher degree of fibrosis associated with significantly worse outcomes.[3] We performed Masson trichrome staining to determine the degree of cardiac fibrosis at 4 weeks after injury and observed significantly decreased fibrosis in hearts of hENPP1mAb-injected animals compared to IgG-injected controls (Figures 4A and 4B). We stratified the severity of fibrosis at 4 weeks post MI according to the ratio of fibrotic area to the left ventricular surface area (severe fibrosis: >40%, moderate fibrosis: 20%–40%, and mild fibrosis: <20%). In IgG-injected animals, 60% of the animals exhibited severe fibrosis at 4 weeks post MI but only 14% of the animals that received hENPP1mAb developed severe fibrosis (Figure 4C; Table S2). In contrast to thin scarred walls in IgG-injected animals, hematoxylin-eosin staining demonstrated thicker walls in the hearts of hENPP1mAb-injected animals suggestive of superior cardiac remodeling (Figure 4D). Peri-infarct hypertrophy is an adverse prognostic sign after MI,[19] and we measured the effects of hENPP1mAb on post-infarct cardiac hypertrophy. At 4 weeks after MI, the heart weight and the heart weight/body weight ratios were significantly decreased in animals that received hENPP1-mAb compared to IgG with no change in body weight alone (Figure 4E). Immunofluorescent staining with myocyte markers demonstrated significantly decreased myocyte size (surface area) in the infarcted hearts of hENPP1mAb-injected animals at 4 weeks after injury consistent with decreased post-infarct hypertrophy (Figure 4F). Immunostaining for endothelial capillaries demonstrated a significant increase in CD31-expressing capillaries suggestive of a superior cardiac repair process (Figure 4G).

hENPP1mAb alters the transcriptional repair response in myocytes and non-myocytes after MI

We next investigated the mechanisms of benefit of hENPP1mAb and performed single-nuclear transcriptomics to determine how the transcriptional repair response is altered by hENPP1-mAb in a cell-specific manner. For this purpose, we harvested the hearts of hENPP1mAb or IgG-treated animals at 7 days following MI and harvested nuclei to perform single-nuclear transcriptomics using the 10× Genomics platform. Uniform manifold approximation and projection (UMAP) analysis demonstrated expected population of cells in the infarcted region including myocytes, macrophages, endothelial cells, and fibroblasts (Figures 5A and S4). Distribution of cells across the IgG and hENPP1mAb populations demonstrated increased myocytes and endothelial cells and decreased fibroblasts and macrophages in the hENPP1mAb group compared to the IgG group (Figures 5B and 5C). We have previously demonstrated that ENPP1 is expressed by the non-myocyte population and predominantly by cardiac fibroblasts.[6] We also validated our observation by examining published single-cell transcriptomic datasets[20] from infarcted human hearts and observed the expression of ENPP1 in the non-myocyte population including human cardiac fibroblasts (Figure S5). Our findings are also consistent with another recently published single-nuclear transcriptomic dataset in individuals with cardiomyopathy that demonstrated ENPP1 expression in myofibroblasts in end-stage cardiomyopathic hearts.[21] We first examined gene expression changes in the entire fibroblast population, and gene ontogeny (GO) analysis demonstrated that cardiac fibroblasts in the hENPP1mAb-treated group exhibited the downregulation of genes related to cytoskeletal organization, extracellular matrix (ECM), cell migration, and cell substrate adhesion (Figure 5D). As this suggested that genes associated with or identifying activated fibroblasts or myofibroblasts were downregulated in the hENPP1mAb group, we performed subcluster analysis of fibroblasts to determine how the fibroblast populations were altered in the hENPP1mAb-treated group. UMAP analysis based on transcriptional signatures was used to subcluster the fibroblasts into 3 populations across both the hENPP1mAb and IgG-treated groups (Figure 5E; Table S6). Fibroblasts of the IgG and the hENPP1mAb-injected hearts were asymmetrically distributed throughout these fibroblast subclusters with fibroblasts from IgG-treated animals predominantly contributing to cluster 0 and fibroblasts from hENPP1mAb-treated animals predominantly contributing to cluster 1 (Figures 5F and 5G). Subcluster 0 represented the myofibroblast population with abundant expression of matrix and myofibroblast genes including Col1a1, Postn, and Acta 2 (Figure 5H) and thus the number of cells contributing to myofibroblasts (cluster 0) in the hENPP1-mAb group was significantly lower compared to the IgG group (Figures 5G and 5H). In contrast, subcluster 1 of the fibroblast population predominantly comprised fibroblasts from the hENPP1mAb-injected animals (Figure 5G). Cluster 1 population of fibroblasts represented a population that exhibited decreased expression of ECM genes such as collagens and myofibroblast genes compared to cluster 0 (Figure 5I). In contrast to Col1 and Col3-encoding genes that were enriched in cluster 0 fibroblasts, cluster 1 fibroblasts expressed collagen genes coding for COL4 or COL5 (Figure 5I) that typically support vasculature and maintain ECM structure.[22,23] Other ECM genes such as *laminin*, *decorin*, and *spondin* that are not typically enriched in scar tissue but thought to play a role in matrix organization, cardiac muscle support,[24] and matricellular signaling[25] were abundantly expressed in cluster 1 compared to cluster 0 (Figure 5I; Table S6). A dot-blot analysis clearly demonstrated that ECM and myofibroblasts signatures were significantly downregulated overall in fibroblasts in the hearts of hENPP1mAb-injected animals (Figure 5J). We confirmed these observations and performed qPCR on the hearts of IgG and hENPP1mAb-treated animals at day 3 and day 7 following MI and observed significant downregulation of ECM and myofibroblast genes in the hearts of hENPP1mAb-injected animals at day 7 post MI (Figure S6A). We also compared the expression of ECM and myofibroblast genes in the hearts of IgG and hENPP1mAb injected animals (day 7 MI) to sham-injured animals and observed that hENPP1mAb significantly altered the transcriptional response with ECM gene expression in the hearts of hENPP1mAb animals closer to sham-injured hearts than that in IgG-injected animals (Figure S6B). The administration of hENPP1mAb thus alters the transcriptional repair response in cardiac fibroblasts and results in an altered fibroblast population with muted myofibroblast and ECM signatures.

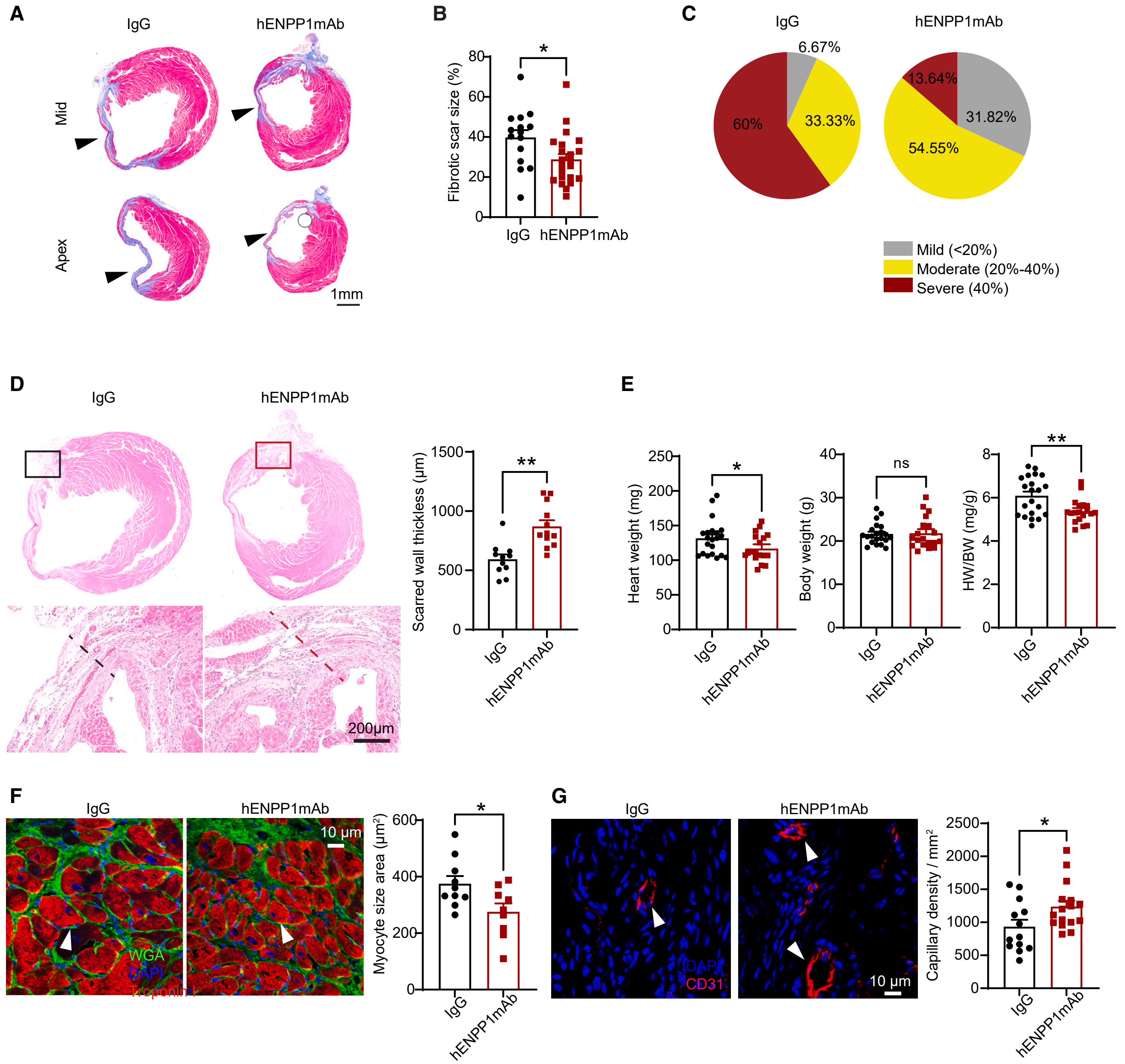

Figure 4. Humanized ENPP1 animals treated with hENPP1mAb after MI exhibit histologic evidence of superior cardiac repair

(A) Masson trichrome staining to demonstrate scar size as a fraction of LV surface area measured 4 weeks after injury at the apex and mid ventricle in IgG or hENPP1mAb-injected humanized ENPP1 animals.

(B) Quantitation of scar surface area (n = 15/IgG and 22/hENPP1mAb) and (C) pie chart illustrating the fraction of animals with mild, moderate, and severe fibrosis following IgG or hENPP1mAb administration.

(D) Hematoxylin/eosin staining to demonstrate the thickness of infarcted wall at 4 weeks after MI in IgG or hENPP1mAb-injected animals with quantification of wall thickness (n = 11/IgG and n = 12/hENPP1mAb).

(E) Heart weight (HW), body weight (BW), and HW/BW ratio in IgG versus hENPP1mAb-treated animals (n = 21/IgG and n = 19/hENPP1mAb).

(F) Immunostaining for cardiac troponin and wheat germ agglutinin to determine myocyte surface area and quantification (surrogate for cardiac muscle hypertrophy) 4 weeks after MI in IgG or hENPP1mAb-injected animals (n = 10/IgG and n = 9/hENPP1mAb).

(G) Staining for endothelial cells (CD31) to determine capillary formation (arrowheads) 4 weeks after MI in IgG or hENPP1mAb-treated animals and quantification of capillary formation (n = 13/IgG and n = 16/hENPP1mAb). Data are represented as mean ± SEM. ** $p < 0.01$, * $p < 0.05$, ns: not significant. Statistical significance was determined using Student's t test, 2 tailed.

CellPress
OPEN ACCESS

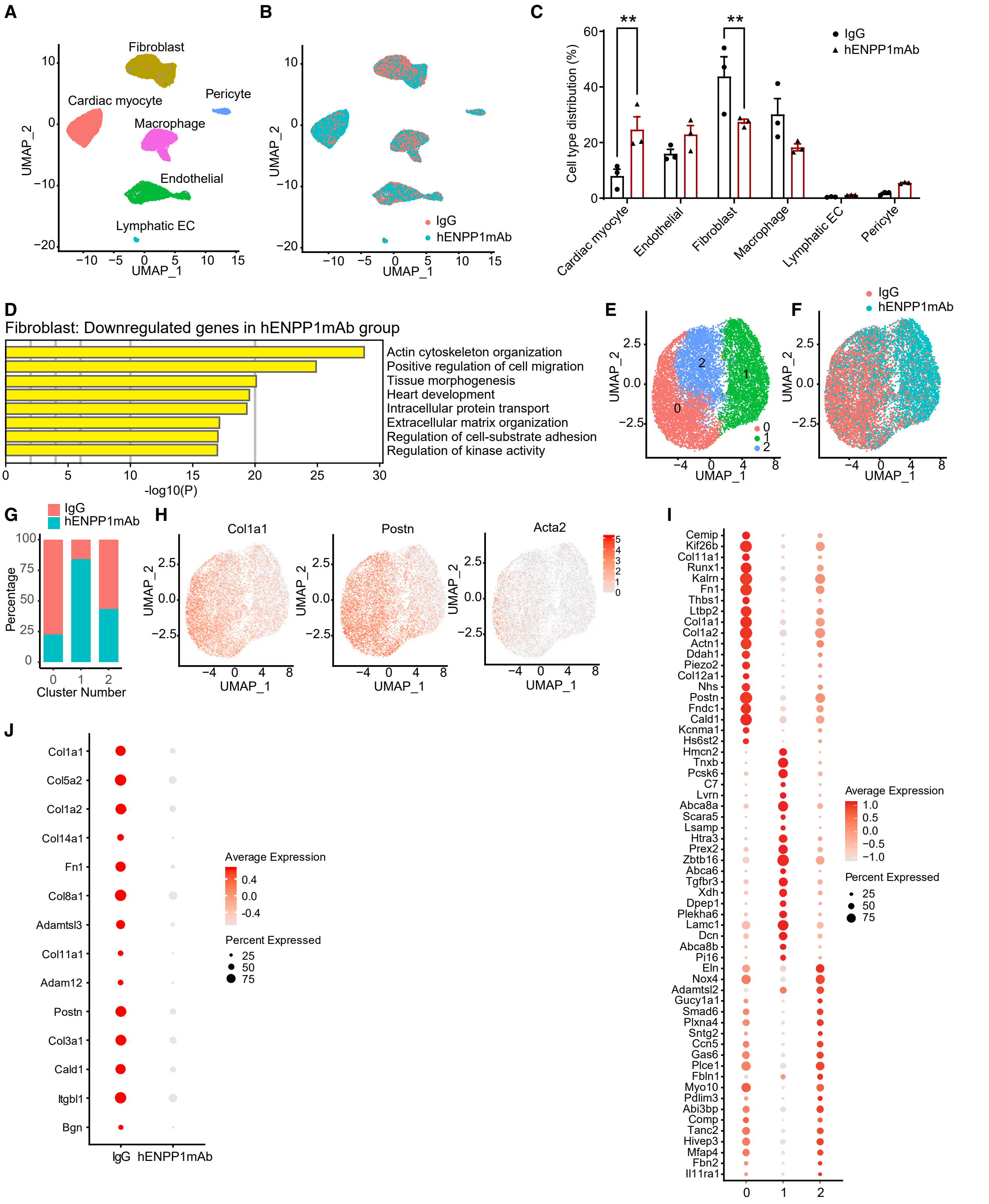

(legend on next page)

As cardiac contractile function and wall tension were increased in the hearts of hENPP1mAb-injected animals, we next examined transcriptional changes in the myocyte population. A GO analysis of genes differentially upregulated in myocytes of hearts of hENPP1mAb versus IgG-injected animals demonstrated genes belonging to metabolic pathways affecting glycolysis, glycogen metabolism, and cardiac contractile process (Figure S7A). Genes regulating glycolysis directly like phosphofructokinase (Pfkm) or Enolase (Eno) or affecting regulation of key glycolytic enzymes were differentially upregulated in the myocytes of hENPP1mAb-injected animals (Figure S7B). Expression of genes encoding for contractile proteins such as (Tnnt2, Tnni3, Myh6, and Ttn) was also significantly upregulated in the myocytes of animals treated with hENPP1mAb (Figure S7C). The facilitation of glycolysis and increased expression of contractile genes provide an underlying mechanism of enhanced post-infarct heart function in hENPP1mAb-injected animals. Increased post-infarct inflammation is associated with adverse infarct outcomes in humans, and we examined the expression of inflammatory gene signatures in macrophages and observed that the expression of inflammatory genes was significantly suppressed in hENPP1-mAb-injected animals (Figure S7D). We have shown that an ENPP1-mediated metabolic cascade increases cell death in the infarcted heart, and we looked at pro-apoptotic signatures across the entire cell population in the infarcted heart and observed that hENPP1mAb decreased cell death signatures (Figure S7E).

hENPP1mAb rescues metabolic defects in the infarcted heart and enhances aerobic cellular respiration of the infarcted heart

ENPP1 is an ectonucleotidase and we have shown previously that ENPP1-mediated hydrolysis initiates a metabolic cascade that disrupts pyrimidine biosynthesis.[6] To determine the effects of hENPP1mAb on the cardiac metabolome, we harvested hearts of IgG versus hENPP1mAb-injected animals at 7 days following MI, extracted metabolites, and performed metabolomic analysis using liquid chromatography and mass spectrometry. We observed that pyrimidines or intermediary metabolites connected with pyrimidine biosynthesis were significantly upregulated in the hearts of hENPP1mAb-injected animals (Figure 6A). Pyrimidines or pyrimidine bases such as uridine, uracil, ornithine, cytidine, and deoxycytidine were increased in the infarcted hearts of hENPP1mAb-injected animals along with increased ribose and metabolites of the pentose phosphate pathway required for ribose synthesis (Figure 6A). A Kyoto Encyclopedia of Genes and Genomes (KEGG) pathway analysis pointed to pyrimidine metabolism as the most significantly differentially expressed metabolic pathway in the hearts of hENPP1mAb versus IgG-injected animals (Figure 6B). Pyrimidine biosynthesis is connected to NAD biosynthesis as PRPP (phosphoribosyl pyrophosphate is required for both), and we had shown previously that ENPP1 metabolic cascade disrupts pyrimidine biosynthesis at the PRPP incorporation step.[6] We examined NAD+/NADH levels and observed the NAD, NADH, and nicotinamide levels to be significantly higher in the infarcted hearts of hENPP1mAb-injected animals (Figure 6C). These findings are overall consistent with increased expression of glycolytic genes in myocytes with hENPP1mAb noted earlier. To determine whether increased NAD levels is associated with increased cellular respiration, we performed Seahorse cellular respiration assays to determine the oxygen consumption rate (OCR) in homogenates of infarcted hearts of hENPP1mAb versus IgG-injected animals. We observed that the OCR measured at various mitochondrial respiratory complexes (complex I and II) was significantly greater in the hearts of hENPP1mAb versus IgG-injected animals (Figure 6D). Taken together, these observations demonstrate the ability of hENPP1mAb to augment or rescue pyrimidine biosynthesis in infarcted hearts and lead to higher NAD levels with significantly higher aerobic cellular respiration.

hENPP1mAb is safe and well tolerated

We next investigated the safety profile of hENPP1mAb and first assessed the biodistribution of hENPP1mAb. For this purpose, we performed pITC-desferrioxamine conjugation to label hENPP1mAb with the radioisotope Zr89, which decays with positron (β+) emission, and intravenously injected a single dose of the radiolabeled hENPP1mAb or similarly labeled IgG. The animals were imaged in a microPET/CT scanner and images acquired serially from 0.5 h after injection to 240 h (Figure S8A). We observed that 24 h after initial administration and distribution, signal intensity from hENPP1mAb was mainly localized to the

Figure 5. Single-nuclei RNA sequencing of hearts of humanized ENPP1 animals treated with IgG or hENPP1mAb and harvested at 7 days following MI

(A) Uniform manifold approximation and projection (UMAP) demonstrating different phenotypes of cell clusters in the infarcted heart and (B) distribution of cells from IgG and hENPP1mAb-treated animals across these clusters (n = 3 animals/group).

(C) Fraction of different cell populations in IgG versus hENPP1mAb-injected animals.

(D) Gene ontology analysis of main pathways differentially downregulated in cardiac fibroblasts in hENPP1mAb-treated animals versus IgG control animals.

(E) UMAP demonstrating subclustering of fibroblast population across IgG and hENPP1mAb groups and (F) distribution of fibroblasts of IgG versus hENPP1mAb groups across these fibroblast subclusters.

(G) Fraction of fibroblasts in IgG or hENPP1mAb-treated groups contributing to the fibroblast subclusters with cluster 0 contributed by IgG-injected group and cluster 1 by the hENPP1mAb group. ($p < 0.05$ in cluster 0, $p < 0.01$ in cluster 1, and no significance in cluster 2).

(H) Expression of ECM and myofibroblast genes (Col1a1, Postn, and Acta2) across these fibroblast subclusters with abundant expression of ECM genes and myofibroblast marker Postn in subcluster 0 compared to subcluster 1.

(I) Dot plot demonstrating distribution of abundantly expressed genes representing the fibroblast subclusters (Note: myofibroblast and ECM genes are abundant in subcluster 0 compared to subcluster 1).

(J) Dot blot representing expression of myofibroblast and ECM genes in the entire cardiac fibroblast population of IgG versus hENPP1mAb animals. Data are expressed as mean ± SEM. $^{**}p < 0.01$. Statistical significance was determined using Student's t test, 2 tailed.

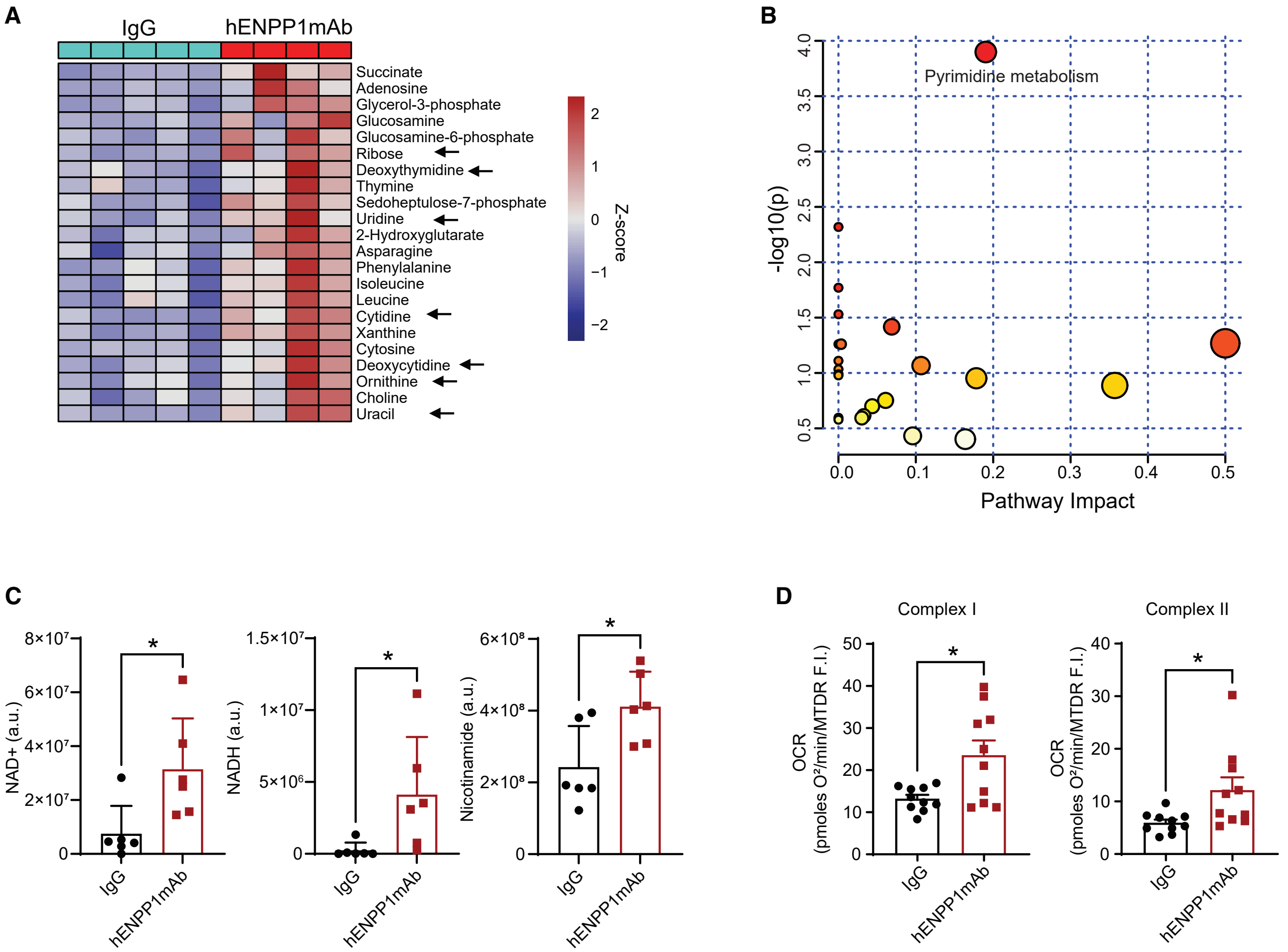

Figure 6. hENPP1mAb administration in humanized mice leads to the rescue of metabolic pathways and augmented cellular respiration in the infarcted heart

(A) Metabolomic analysis of hearts of animals injected with IgG or hENPP1mAb at day 7 post MI demonstrating metabolites that are significantly upregulated ($p < 0.05$) in hearts of hENPP1mAb-injected animals (arrows point to pyrimidines or metabolites in pentose phosphate pathway, demonstrating rescue of pyrimidines in hENPP1mAb-injected animals compared to IgG-injected animals, n = 5/IgG and 4/hENPP1mAb).

(B) KEGG analysis of metabolic pathways that are significantly upregulated in hearts of hENPP1mAb injected animals demonstrating that pyrimidine biosynthetic pathway is the most significant metabolic pathway.

(C) Quantification of NAD, NADH, and nicotinamide levels in hearts of animals injected with hENPP1mAb versus IgG as early as 3 days post infarction (n = 6/group).

(D) Seahorse cellular respiration on heart homogenates (day 3 post MI) demonstrating oxygen consumption rate (OCR) at mitochondrial electron transport complexes I and II normalized to MitoTracker Deep Red (MTDR) fluorescence (n = 10/group). Data are expressed as mean ± SEM. $^{**}p < 0.01$. Statistics was determined using Student's t test, 2 tailed.

heart (9.10 ± 0.18 %ID/cc), liver (8.40 ± 0.11 %ID/cc), and bones (4.63 ± 0.28 %ID/cc) (Figure S8A). At 144 h after initial administration, the signal intensity of hENPP1mAb compared to that from IgG was significantly higher in multiple organs including heart (5.83 ± 0.30 vs. 1.90 ± 0.09 %ID/cc), liver (5.81 ± 0.21 vs. 5.01 ± 0.19 %ID/cc), kidneys (3.82 ± 0.10 vs. 2.09 ± 0.28 %ID/cc), lungs (3.25 ± 0.14 vs. 1.40 ± 0.20 %ID/cc), and bones (8.60 ± 0.61 vs. 2.53 ± 0.38 %ID/cc) confirming target-specific organ-wide distribution of hENPP1mAb (Figure S8B). In the heart, we observed a significant drop in signal intensity from 24 to 72 h that likely corresponds to the clearance of a humanized IgG from mice (Figure S8C).

To determine any potential toxicity associated with the hENPP1mAb, we administered the antibody to humanized ENPP1 animals every 3 days for 2 weeks as performed earlier for therapeutic intervention and harvested the organs at 4 weeks after the first dose was administered. Histology did not show any evidence of toxicity in major organs such as the lung, heart, liver, kidney, and spleen (Figure S8D).

As osteoblasts in bone express ENPP1[26] and hENPP1mAb was observed to bind to bone tissue in the biodistribution study, we administered hENPP1mAb for 2 weeks as aforementioned and then performed CT scans at 4 weeks after initial dose to determine changes in bone densitometry, but we observed

that hENPP1mAb did not have any effects on bone density (Figure S9A). Loss-of-function mutations in ENPP1 have been associated with a rare genetic disease that causes ectopic calcification in neonatal life in humans.[27,28] We harvested the aorta, heart, kidney, and heart valves at 4 weeks following the initial administration of hENPP1mAb as aforementioned, but Von Kossa staining did not demonstrate any calcification of tissues (Figure S9B). Serum chemistry including calcium and phosphate levels following 2 weeks of hENPP1mAb treatment did not demonstrate any abnormalities and no differences were observed compared to IgG-injected animals (Table S3). Complete and differential blood counts also did not demonstrate any abnormality following 2 weeks of hENPP1mAb administration (Table S3). There was also no evidence of acute toxicity associated with hENPP1mAb administration. At day 3 following hENPP1mAb administration, serum chemistry and blood counts were within the normal range and no different from that of IgG-injected animals (Table S4). There was no evidence of weight loss compared to baseline at day 3 or 1 and 2 weeks following hENPP1mAb administration (Table S4; Figure S9C). As ENPP1 has been shown to affect B cell function,[29] we also examined B cell subsets from the peripheral circulation and the spleen by flow cytometry following injection of hENPP1mAb, but did not observe any difference in B cell subsets compared to IgG-injected animals within 7 days of hENPP1mAb/IgG injection (Figure S10). These data suggest that hENPP1mAb is not associated with any obvious signs of toxicity in the organs examined.

A single "shot" of hENPP1mAb after cardiac injury is sufficient to enhance repair and rescue post-infarct heart function

We had administered the hENPP1mAb prior to infarction with repeated administration after MI to account for the time needed for the hENPP1mAb to be systemically absorbed via i.p. route and the shorter half-life of human IgG in mice. However, administration of a drug prior to a clinical event is not a clinically viable strategy. To overcome issues of pharmacokinetics of human IgG in mice, we adopted to use the Tg32 mice (FcRN−/− hFcRn (32) T). These animals have the murine *Fcgrt* gene (Fc receptor, IgG alpha chain transporter) knocked out and carry a transgene expressing the human FCGRT gene under its native promoter (Tg32).[30] These animals thus demonstrate antibody clearance similar to humans and are extremely useful for determining the half-life of humanized monoclonal antibodies in mice, and pharmacokinetic data obtained from these animals correspond closely with phase 1 data in human trials.[16,31] We first used the Tg32 animal to determine the half-life of the hENPP1mAb. For this purpose, we injected a single dose (10 mg/kg) of the antibody intravenously and sampled peripheral blood at various time points following administration to determine the concentration of hENPP1mAb (by measuring human IgG) in peripheral blood of mice. Control animals received polyclonal human IgG and using this system, we determined an approximate half-life of 14 days in Tg32 animals with the clearance of the hENPP1-mAb closely mirroring that of human IgG (Table S5). It is to be noted that as our pK measurements are being carried out in Tg32 animals, that do not express human ENPP1, it does not take into account target-mediated drug disposition as a potential factor regulating the clearance of the drug. We have shown previously that ENPP1 expression peaks at 7 days after MI and the injured region undergoes maximal transcriptional changes within the first 2 weeks after MI.[6,22] As the estimated hENPP1mAb half-life was 14 days, we hypothesized that a single dose of the hENPP1mAb administered after injury should be sufficient to maintain the inhibition of hENPP1 and lead to superior cardiac repair. To address this hypothesis, we crossed humanized ENPP1 animals with Tg32 animals to create progeny humanized ENPP1/Tg32 animals that were homozygous for all alleles (Figure 7A). We subjected these animals to MI and injected a single dose of hENPP1mAb (100 mg/kg) or control IgG intravenously on the day of injury within 2–4 h of MI (Figure 7B). We adopted to administer a higher dose to serve as a loading dose that provides advantages of rapidly achieving high plasma levels and drives an immediate clinical response. Echocardiography was performed at weekly intervals and B and M mode echocardiography demonstrated significantly superior cardiac contractile performance with the EF and FS almost double that of IgG-injected animals (EF at 7 days: 42.73% ± 5.07% in hENPP1mAb versus 23.96% ± 4.73% in IgG groups; FS at 7 days: 21.56% ± 2.95% in hENPP1mAb-injected animals versus 11.37% ± 2.51% in IgG-injected controls, $p < 0.05$) (Figures 7C and 7D). The dilatation of the ventricle, measured by end systolic ventricular dimension (LVIDs), was also significantly less in hENPP1-mAb-injected animals compared to IgG-injected controls (Figures 7C and 7D). Measurement of adverse post-infarct hypertrophy by determining heart weight and body weight ratios at 4 weeks following injury demonstrated significantly decreased post-infarct hypertrophy in hENPP1mAb-injected animals without any change in body weight (Figure 7E). Finally, histological analysis with Masson trichome staining at 4 weeks post MI demonstrated significantly decreased fibrosis in the hearts of animals injected with a single dose of hENPP1mAb (Figure 7F). These observations demonstrate that a single dose of hENPP1-mAb is sufficient to rescue post-MI heart function.

DISCUSSION

Cardiac repair comprises a complex and highly regulated sequence of spatiotemporal events driven by the recruitment of a diverse population of cells in the infarcted region. Crosstalk between cells regulates critical repair processes such as fibrosis, inflammation, cell death, and post-infarct cardiac remodeling. The field of cardiac regenerative therapies has evolved from a myocyte-centric approach to an understanding that cellular crosstalk between different population of cells in the injured heart holds immense therapeutic potential.[5,32] This body of work targets a myocyte-non-myocyte crosstalk that regulates extracellular nucleotide metabolism in the infarcted heart, and demonstrates that inhibiting a metabolic cascade that is initiated by ENPP1 rescues metabolic defects, induces beneficial transcriptional repair response across myocyte and non-myocyte populations, and leads to superior post-infarct heart function. Of all the mammalian ectonucleotidases expressed in the infarcted heart, genetic loss-of-function studies have demonstrated that ENPP1 is the principal ectonucleotidase mediating extracellular ATP hydrolysis.[6] Our strategy thus builds on this

CellPress
OPEN ACCESS

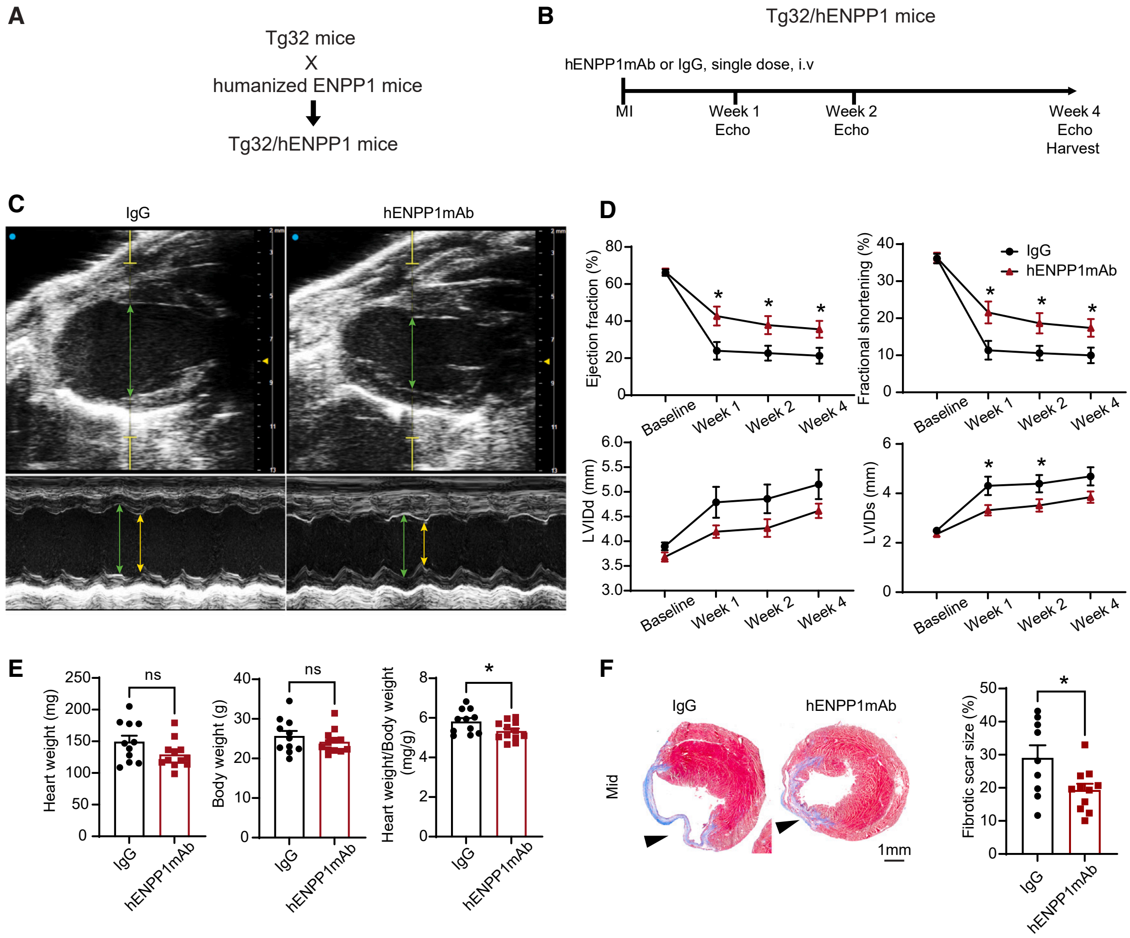

Figure 7. A single dose of hENPP1mAb administered in humanized ENPP1/Tg32 animals after MI is sufficient to significantly rescue post-infarct cardiac function

(A) Genetic strategy of generating humanized ENPP1/Tg32 animals and (B) determining the effects of a single dose of hENPP1mAb administered after MI.

(C) B mode (top) and M mode (below) echocardiogram demonstrating cardiac contractile function and chamber dilatation in IgG versus hENPP1mAb-injected animals. Green line points to cardiac dimensions in diastole and yellow lines point to dimensions in systole.

(D) Ejection fraction, fractional shortening, and LV dimensions in systole (LVIds) and diastole (LVIDd) at 1, 2, and 4 weeks after MI following a single shot of hENPP1mAb or IgG after MI (n = 11/IgG and n = 12/hENPP1mAb).

(E) Heart weight, body weight, and heart weight/body weight ratios of hearts harvested at 4 weeks in animals receiving a single dose of hENPP1mAb or IgG after MI (n = 11/IgG and n = 12/hENPP1mAb).

(F) Masson trichrome staining to demonstrate fibrosis at 4 weeks post MI in animals receiving a single dose of hENPP1mAb or IgG and quantification of fibrosis (n = 9/IgG and n = 11/hENPP1mAb). Data represented as mean ± SEM, ** $p < 0.01$, * $p < 0.05$, ns: not significant. Statistical significance was determined using unpaired multiple t test (D) or Student's t test, 2 tailed (E and F).

rational premise that if deleterious metabolic cascades initiated by the principal cardiac ectonucleotidase ENPP1 can be attenuated, then an adverse metabolic catastrophe in cycling and non-cycling cells can be avoided with beneficial effects on post-infarct cardiac function. Rather than affecting a specific phenotype of cell or cellular process, hENPP1mAb-mediated inhibition of ENPP1 by rescuing defects in pyrimidine and NAD metabolism affected a diverse population of cells including myocytes, fibroblasts, endothelial cells, and inflammatory cells. Such pleiotropic beneficial effects on a diverse population of cells result in decreased fibroblast activation, decreased expression of ECM genes, increased glycolysis, and improved myocyte energetics with increased contractility. It is to be noted that even though hENPP1mAb decreased fibrosis and ECM gene expression, there was no occurrence of cardiac rupture. This is likely because of timing of peak ENPP1 expression. Cardiac rupture typically occurs within the first few days of MI, but peak ENPP1 expression occurs in the heart at 7 days after injury and thus beyond the stage when the infarcted heart is most vulnerable to rupture. As such, targeting ENPP1 decreased ECM gene expression without increasing rupture risk. The beneficial metabolic effects on myocytes also likely resulted in superior myocyte function, which decreases the chance of post-infarct rupture. Thus, hENPP1mAb by exerting beneficial effects on different cardiac cell populations in cardiac repair, whether direct or secondary, stands to have an advantage

over therapeutic strategies aimed at targeting focused processes such as modulation of fibrosis or angiogenesis.

Our study has limitations as well. Modulation of metabolism in the human heart remains an untested therapeutic strategy for MI or prevention of heart failure, though metabolic abnormalities are thought to contribute toward worsening heart function. In this regard, although our study demonstrates the therapeutic efficacy of the hENPP1mAb injected after the occurrence of MI, the transcriptomic, phenotypic, and metabolic data reflect the delivery of the therapeutic prior to MI. However, the functional benefits were similar when the hENPP1mAb was administered after MI, suggesting that mechanisms of benefit of hENPP1mAb including effects on pyrimidine and NAD biosynthesis likely underlie mechanisms of benefit when the therapeutic agent is injected within a few hours of MI. The therapeutic window after MI during which hENPP1mAb would continue to exert beneficial results is also not clear from our study as the hENPP1mAb was administered within a few hours of infarction. However, as wound healing events occur rapidly after cardiac injury, early administration of hENPP1mAb would likely offer the maximal clinical benefit. Our study also did not examine in great detail potential safety concerns related to antibody-dependent cytotoxicity, but we did not see any occurrence of cardiac rupture with no difference in post-MI mortality, thus suggesting that antibody-dependent cellular cytotoxicity, even if present, was not of sufficient magnitude to affect clinical outcomes. Mutations in ENPP1 are known to cause generalized ectopic calcification in neonatal life and hypophosphatemic rickets in adulthood,[33] so our therapeutic strategy should be accompanied by careful monitoring for ectopic calcification and calcium and phosphate balance for a few months following administration of hENPP1mAb. Although we did not see any calcification in the short term in the animals that received hENPP1mAb, the degree and pattern of expression of ENPP1 in the humanized knockin mice that we have used was not extensively evaluated in all tissues, emphasizing the need for thorough monitoring of ectopic calcification and other potential adverse effects on bone mass and calcium/phosphate balance.

Notwithstanding the aforementioned limitations, prevention of coronary thrombosis and attenuating the sympatho-adrenergic and renin-angiotensin system form the mainstay of therapies for MI. There are no drugs currently available that augment tissue repair in the heart or other organs. In this regard, this body of work identifies a monoclonal antibody, we have engineered to affect cardiac repair, by beneficially modulating post-infarct metabolism in both myocytes and non-myocytes. Transcriptional changes after MI occur rapidly and the infarcted region is known to become transcriptionally mature within 2 weeks of injury.[22] As the half-life of the hENPP1mAb is anticipated to be close to 14 days in humans, a single dose of the hENPP1mAb administered after MI would potentially be sufficient to enhance heart repair and attenuate post-infarct decline in heart function.

Limitations of the study

Cardiac metabolism has not been therapeutically targeted for MI or heart failure in humans and thus this remains an untested strategy. ENPP1 mutations can potentially lead to calcium and phosphate imbalance, and future human phase 1 studies would be needed to determine the safety of the monoclonal antibody. Our study also uses a single-dose-based strategy for MI based on the half-life of the antibody as well as on the native repair response of the injured heart. Our study does not address multi-dose safety particularly if the therapeutic is envisioned to treat chronic heart failure.

RESOURCE AVAILABILITY

Lead contact

Further information and requests for resources and reagents should be directed to and will be fulfilled by the lead contact, Arjun Deb (adeb@mednet.ucla.edu).

Materials availability

There are certain restrictions to the availability of the hENPP1mAb. The humanized antibody is manufactured by a CRO in batches and manufacturing is expensive and thus may limit the amounts we are able to provide to investigators. Requests for the hENPP1mAb or other unique reagents generated in this study should be directed to and will be fulfilled by the lead contact, Arjun Deb (adeb@mednet.ucla.edu), with a Materials Transfer Agreement.

Data and code availability

Original/source data of RNA sequencing (accession number: GEO: GSE225826) have been deposited at https://www.ncbi.nlm.nih.gov/geo/ and are publicly available as of the date of publication. This study did not report new original code. Any additional information required to reanalyze the data reported in this paper is available from the lead contact upon request.

ACKNOWLEDGMENTS

We acknowledge Retrogenix Labs for performing the immunoblotting studies with hENPP1mAb. We thank Curia Global, Inc. as contract research organization for manufacturing the hENPP1mAb. We thank Jackson Laboratory for performing half-life experiments in Tg32 mice. We thank Juan F. Alvarez, UCLA, for maintaining and genotyping the humanized ENPP1 and Tg32 animals. This study was funded by the NIH (DK132735, HL169217, HL149658, HL152176, and HL149687), California Institute of Regenerative Medicine (CIRM TRAN 1-12893), and the Department of Defense (PR161247 and PR190268). The GNEXT microPET/CT scanner (Sofie Biosciences) was funded by the NIH S10 Shared Instrumentation for Animal Research Grant (1 S10 OD026917-01A1).

AUTHOR CONTRIBUTIONS

S.L. performed all the experiments, collected and analyzed the data, and contributed to writing the manuscript. B.T., P.W., and B.S. performed all animal surgeries. F.M., Y.G., and M.P. performed single-nuclear analysis and J.W. performed plasmid construction. S.R., L. Su, and Q.S. assisted in bench experiments. J.T.H. and T.G. completed all metabolomic studies, D.B.K. performed computational homology modeling, and M.T., R.T., J.C., R.M.v.D., and S.X. helped in antibody biodistribution and cardiac CT studies. L. Stiles and O.S. performed mitochondrial seahorse experiments and data analysis. E.M.-R. and K.D. performed B cell function analysis. A.D. conceptualized the project, designed the experiments, supervised data collection and analysis, and wrote the manuscript.

DECLARATION OF INTERESTS

The intellectual property associated with hENPP1mAb is held by the Regents, University of California.

STAR★METHODS

Detailed methods are provided in the online version of this paper and include the following:

CellPress
OPEN ACCESS

- KEY RESOURCES TABLE
- EXPERIMENTAL MODEL AND STUDY PARTICIPANT DETAILS
  - Animal care and use
  - Generation of humanized ENPP1 mice
  - Myocardial infarction and hENPP1mAb administration in humanized ENPP1 mice or ENPP1/Tg32 mice
  - Biodistribution studies of hENPP1mAb *in vivo*
- METHOD DETAILS
  - Generation of humanized hENPP1mAb
  - Homology modeling and protein-protein docking of hENPP1mAb with human ENPP1
  - Assessment of binding profile of hENPP1mAb using the Retrogenix membrane array
  - Potency curves of hENPP1mAb in inhibiting human ENPP1 catalytic activity
  - Plasmid construction and transfection
  - Human ENPP3/human CD39/human CD73/human ENPP4/human ENPP5 overexpression verification
  - hENPP1 mAb binding affinity/avidity
  - RT-PCR and gel electrophoresis to distinguish mouse and human ENPP1mRNA sequence
  - RNA extraction and Q-PCR
  - Histology studies
  - Immunoblotting
  - Echocardiography
  - Ectonucleotidase assay of injured heart tissue
  - *In vivo* microCT imaging
  - Single nuclei RNA sequencing
  - *In vivo* toxicity test
  - Complete blood count and serum biochemistry
  - B cell function analysis
  - Metabolomics
  - Seahorse respiration assay
  - Pharmacokinetic analysis of hENPP1mAb in Tg32 homozygous mice
- QUANTIFICATION AND STATISTICAL ANALYSIS

SUPPLEMENTAL INFORMATION

Supplemental information can be found online at https://doi.org/10.1016/j.xcrm.2024.101795.

Received: March 7, 2024
Revised: September 6, 2024
Accepted: September 27, 2024
Published: October 24, 2024

REFERENCES

1. Frangogiannis, N.G. (2017). The extracellular matrix in myocardial injury, repair, and remodeling. J. Clin. Invest. *127*, 1600–1612. https://doi.org/10.1172/JCI87491.
2. Ziaeian, B., and Fonarow, G.C. (2016). Epidemiology and aetiology of heart failure. Nat. Rev. Cardiol. *13*, 368–378. https://doi.org/10.1038/nrcardio.2016.25.
3. Gulati, A., Jabbour, A., Ismail, T.F., Guha, K., Khwaja, J., Raza, S., Morarji, K., Brown, T.D.H., Ismail, N.A., Dweck, M.R., et al. (2013). Association of fibrosis with mortality and sudden cardiac death in patients with nonischemic dilated cardiomyopathy. JAMA, J. Am. Med. Assoc. *309*, 896–908. https://doi.org/10.1001/jama.2013.1363.
4. Frangogiannis, N.G. (2015). Pathophysiology of Myocardial Infarction. Compr. Physiol. *5*, 1841–1875. https://doi.org/10.1002/cphy.c150006.
5. Tzahor, E., and Dimmeler, S. (2022). A coalition to heal-the impact of the cardiac microenvironment. Science *377*, eabm4443. https://doi.org/10.1126/science.abm4443.
6. Li, S., Yokota, T., Wang, P., Ten Hoeve, J., Ma, F., Le, T.M., Abt, E.R., Zhou, Y., Wu, R., Nanthavongdouangsy, M., et al. (2022). Cardiomyocytes disrupt pyrimidine biosynthesis in nonmyocytes to regulate heart repair. J. Clin. Invest. *132*, e149711. https://doi.org/10.1172/JCI149711.
7. Vénéreau, E., Ceriotti, C., and Bianchi, M.E. (2015). DAMPs from Cell Death to New Life. Front. Immunol. *6*, 422. https://doi.org/10.3389/fimmu.2015.00422.
8. Burnstock, G. (2017). Purinergic Signaling in the Cardiovascular System. Circ. Res. *120*, 207–228. https://doi.org/10.1161/CIRCRESAHA.116.309726.
9. Kato, K., Nishimasu, H., Mihara, E., Ishitani, R., Takagi, J., Aoki, J., and Nureki, O. (2012). Expression, purification, crystallization and preliminary X-ray crystallographic analysis of Enpp1. Acta Crystallogr., Sect. F: Struct. Biol. Cryst. Commun. *68*, 778–782. https://doi.org/10.1107/S1744309112019306.
10. Gao, S.H., Huang, K., Tu, H., and Adler, A.S. (2013). Monoclonal antibody humanness score and its applications. BMC Biotechnol. *13*, 55. https://doi.org/10.1186/1472-6750-13-55.
11. Dennis, M.L., Newman, J., Dolezal, O., Hattarki, M., Surjadi, R.N., Nuttall, S.D., Pham, T., Nebl, T., Camerino, M., Khoo, P.S., et al. (2020). Crystal structures of human ENPP1 in apo and bound forms. Acta Crystallogr. D Struct. Biol. *76*, 889–898. https://doi.org/10.1107/S2059798320010505.
12. Kato, K., Nishimasu, H., Okudaira, S., Mihara, E., Ishitani, R., Takagi, J., Aoki, J., and Nureki, O. (2012). Crystal structure of Enpp1, an extracellular glycoprotein involved in bone mineralization and insulin signaling. Proc. Natl. Acad. Sci. USA *109*, 16876–16881. https://doi.org/10.1073/pnas.1208017109.
13. Borza, R., Salgado-Polo, F., Moolenaar, W.H., and Perrakis, A. (2022). Structure and function of the ecto-nucleotide pyrophosphatase/phosphodiesterase (ENPP) family: Tidying up diversity. J. Biol. Chem. *298*, 101526. https://doi.org/10.1016/j.jbc.2021.101526.
14. Arnold, N.D., Pickworth, J.A., West, L.E., Dawson, S., Carvalho, J.A., Casbolt, H., Braithwaite, A.T., Iremonger, J., Renshall, L., Germaschewski, V., et al. (2019). A therapeutic antibody targeting osteoprotegerin attenuates severe experimental pulmonary arterial hypertension. Nat. Commun. *10*, 5183. https://doi.org/10.1038/s41467-019-13139-9.
15. Turner, L., Lavstsen, T., Berger, S.S., Wang, C.W., Petersen, J.E.V., Avril, M., Brazier, A.J., Freeth, J., Jespersen, J.S., Nielsen, M.A., et al. (2013). Severe malaria is associated with parasite binding to endothelial protein C receptor. Nature *498*, 502–505. https://doi.org/10.1038/nature12216.
16. Betts, A., Keunecke, A., van Steeg, T.J., van der Graaf, P.H., Avery, L.B., Jones, H., and Berkhout, J. (2018). Linear pharmacokinetic parameters for monoclonal antibodies are similar within a species and across different pharmacological targets: A comparison between human, cynomolgus monkey and hFcRn Tg32 transgenic mouse using a population-modeling approach. mAbs *10*, 751–764. https://doi.org/10.1080/19420862.2018.1462429.
17. Daniels, L.J., Macindoe, C., Koutsifeli, P., Annandale, M., James, S.L., Watson, L.E., Coffey, S., Raaijmakers, A.J.A., Weeks, K.L., Bell, J.R., et al. (2023). Myocardial deformation imaging by 2D speckle tracking echocardiography for assessment of diastolic dysfunction in murine cardiopathology. Sci. Rep. *13*, 12344. https://doi.org/10.1038/s41598-023-39499-3.
18. Lopez-Candales, A., and Hernandez-Suarez, D.F. (2017). Strain Imaging Echocardiography: What Imaging Cardiologists Should Know. Curr. Cardiol. Rev. *13*, 118–129. https://doi.org/10.2174/1573403X12666161028122649.
19. Kramer, C.M., Rogers, W.J., Park, C.S., Seibel, P.S., Shaffer, A., Theobald, T.M., Reichek, N., Onodera, T., and Gerdes, A.M. (1998). Regional myocyte hypertrophy parallels regional myocardial dysfunction during post-infarct remodeling. J. Mol. Cell. Cardiol. *30*, 1773–1778. https://doi.org/10.1006/jmcc.1998.0741.
20. Kuppe, C., Ramirez Flores, R.O., Li, Z., Hayat, S., Levinson, R.T., Liao, X., Hannani, M.T., Tanevski, J., Wünnemann, F., Nagai, J.S., et al. (2022). Spatial multi-omic map of human myocardial infarction. Nature *608*, 766–777. https://doi.org/10.1038/s41586-022-05060-x.

21. Chaffin, M., Papangeli, I., Simonson, B., Akkad, A.D., Hill, M.C., Arduini, A., Fleming, S.J., Melanson, M., Hayat, S., Kost-Alimova, M., et al. (2022). Single-nucleus profiling of human dilated and hypertrophic cardiomyopathy. Nature *608*, 174–180. https://doi.org/10.1038/s41586-022-04817-8.

22. Yokota, T., McCourt, J., Ma, F., Ren, S., Li, S., Kim, T.H., Kurmangaliyev, Y.Z., Nasiri, R., Ahadian, S., Nguyen, T., et al. (2020). Type V Collagen in Scar Tissue Regulates the Size of Scar after Heart Injury. Cell *182*, 545–562.e23. https://doi.org/10.1016/j.cell.2020.06.030.

23. Vahedi, K., and Alamowitch, S. (2011). Clinical spectrum of type IV collagen (COL4A1) mutations: a novel genetic multisystem disease. Curr. Opin. Neurol. *24*, 63–68. https://doi.org/10.1097/WCO.0b013e32834232c6.

24. Yap, L., Wang, J.W., Moreno-Moral, A., Chong, L.Y., Sun, Y., Harmston, N., Wang, X., Chong, S.Y., Vanezis, K., Öhman, M.K., et al. (2019). In Vivo Generation of Post-infarct Human Cardiac Muscle by Laminin-Promoted Cardiovascular Progenitors. Cell Rep. *26*, 3231–3245.e9. https://doi.org/10.1016/j.celrep.2019.02.083.

25. Zhang, W., Ge, Y., Cheng, Q., Zhang, Q., Fang, L., and Zheng, J. (2018). Decorin is a pivotal effector in the extracellular matrix and tumour microenvironment. Oncotarget *9*, 5480–5491. https://doi.org/10.18632/oncotarget.23869.

26. Gao, M.M., Su, Q.N., Liang, T.Z., Ma, J.X., Liang, T.Z., Stoddart, M.J., Richards, R.G., Zhou, Z.Y., and Zou, N.X. (2018). Transcriptional activation of ENPP1 by osterix in osteoblasts and osteocytes. Eur. Cell. Mater. *36*, 1–14. https://doi.org/10.22203/eCM.v036a01.

27. Roberts, F., Zhu, D., Farquharson, C., and Macrae, V.E. (2019). ENPP1 in the Regulation of Mineralization and Beyond. Trends Biochem. Sci. *44*, 616–628. https://doi.org/10.1016/j.tibs.2019.01.010.

28. Kalal, I.G., Seetha, D., Panda, A., Nitschke, Y., and Rutsch, F. (2012). Molecular diagnosis of generalized arterial calcification of infancy (GACI). J. Cardiovasc. Dis. Res. *3*, 150–154. https://doi.org/10.4103/0975-3583.95373.

29. Wang, H., Gonzalez-Garcia, I., Traba, J., Jain, S., Conteh, S., Shin, D.M., Qi, C., Gao, Y., Sun, J., Kang, S., et al. (2017). ATP-degrading ENPP1 is required for survival (or persistence) of long-lived plasma cells. Sci. Rep. *7*, 17867. https://doi.org/10.1038/s41598-017-18028-z.

30. Roopenian, D.C., Christianson, G.J., and Sproule, T.J. (2010). Human FcRn transgenic mice for pharmacokinetic evaluation of therapeutic antibodies. Methods Mol. Biol. *602*, 93–104. https://doi.org/10.1007/978-1-60761-058-8_6.

31. Avery, L.B., Wang, M., Kavosi, M.S., Joyce, A., Kurz, J.C., Fan, Y.Y., Dowty, M.E., Zhang, M., Zhang, Y., Cheng, A., et al. (2016). Utility of a human FcRn transgenic mouse model in drug discovery for early assessment and prediction of human pharmacokinetics of monoclonal antibodies. mAbs *8*, 1064–1078. https://doi.org/10.1080/19420862.2016.1193660.

32. Rurik, J.G., Tombácz, I., Yadegari, A., Méndez Fernández, P.O., Shewale, S.V., Li, L., Kimura, T., Soliman, O.Y., Papp, T.E., Tam, Y.K., et al. (2022). CAR T cells produced in vivo to treat cardiac injury. Science *375*, 91–96. https://doi.org/10.1126/science.abm0594.

33. Li, Q., Guo, H., Chou, D.W., Berndt, A., Sundberg, J.P., and Uitto, J. (2013). Mutant Enpp1asj mice as a model for generalized arterial calcification of infancy. Dis. Model. Mech. *6*, 1227–1235. https://doi.org/10.1242/dmm.012765.

34. Li, S., Yokota, T., Wang, P., Ten Hoeve, J., Ma, F., Le, T.M., Abt, E.R., Zhou, Y., Wu, R., Nanthavongdouangsy, M., et al. (2022). Cardiomyocyte disrupts pyrimidine biosynthesis in non-myocytes to regulate heart repair. J. Clin. Invest. *132*, e149711. https://doi.org/10.1172/JCI149711.

35. Schwaiger, M., and Wester, H.J. (2011). How Many PET Tracers Do We Need? J. Nucl. Med. *52*, 36s–41s. https://doi.org/10.2967/jnumed.110.085738.

36. Gu, Z., Taschereau, R., Vu, N.T., Prout, D.L., Lee, J., and Chatziioannou, A.F. (2020). Performance evaluation of HiPET, a high sensitivity and high resolution preclinical PET tomograph. Phys. Med. Biol. *65*, 045009. https://doi.org/10.1088/1361-6560/ab6b44.

37. (2022). Molecular Operating Environment (MOE). Chemical Computing Group ULC, 1010 Sherbrooke St. West, Suite #910, Montreal, QC, Canada, H3A 2R7.

38. (2022). Pro_model, Scientific Vector Language (SVL). Source code provided by Chemical Computing Group ULC, 1010 Sherbrooke St. West, Suite #910, Montreal, QC, Canada, H3A 2R7.

39. Mensali, N., Köksal, H., Joaquina, S., Wernhoff, P., Casey, N.P., Romecin, P., Panisello, C., Rodriguez, R., Vimeux, L., Juzeniene, A., et al. (2023). ALPL-1 is a target for chimeric antigen receptor therapy in osteosarcoma. Nat. Commun. *14*, 3375. https://doi.org/10.1038/s41467-023-39097-x.

40. Freeth, J., and Soden, J. (2020). New Advances in Cell Microarray Technology to Expand Applications in Target Deconvolution and Off-Target Screening. SLAS Discov. *25*, 223–230. https://doi.org/10.1177/2472555219897567.

41. Yoon, Y.S., Uchida, S., Masuo, O., Cejna, M., Park, J.S., Gwon, H.C., Kirchmair, R., Bahlman, F., Walter, D., Curry, C., et al. (2005). Progressive attenuation of myocardial vascular endothelial growth factor expression is a seminal event in diabetic cardiomyopathy - Restoration of microvascular homeostasis and recovery of cardiac function in diabetic cardiomyopathy after replenishment of local vascular endothelial growth factor. Circulation *111*, 2073–2085. https://doi.org/10.1161/01.Cir.0000162472.52990.36.

42. Hsu, J.J., Lu, J., Umar, S., Lee, J.T., Kulkarni, R.P., Ding, Y., Chang, C.C., Hsiai, T.K., Hokugo, A., Gkouveris, I., et al. (2018). Effects of teriparatide on morphology of aortic calcification in aged hyperlipidemic mice. Am. J. Physiol. Heart Circ. Physiol. *314*, H1203–H1213. https://doi.org/10.1152/ajpheart.00718.2017.

43. Pioli, P.D., Casero, D., Montecino-Rodriguez, E., Morrison, S.L., and Dorshkind, K. (2019). Plasma Cells Are Obligate Effectors of Enhanced Myelopoiesis in Aging Bone Marrow. Immunity *51*, 351–366.e6. https://doi.org/10.1016/j.immuni.2019.06.006.

44. Montecino-Rodriguez, E., Estrada, O.I., and Dorshkind, K. (2024). Transient PU.1 low fetal progenitors generate lymphoid progeny that contribute to adult immunity. Life Sci. Alliance *7*, e202402629. https://doi.org/10.26508/lsa.202402629.

45. Acin-Perez, R., Benador, I.Y., Petcherski, A., Veliova, M., Benavides, G.A., Lagarrigue, S., Caudal, A., Vergnes, L., Murphy, A.N., Karamanlidis, G., et al. (2020). A novel approach to measure mitochondrial respiration in frozen biological samples. EMBO J. *39*, e104073. https://doi.org/10.15252/embj.2019104073.

46. Low, B.E., Christianson, G.J., Lowell, E., Qin, W., and Wiles, M.V. (2020). Functional humanization of immunoglobulin heavy constant gamma 1 Fc domain human *FCGRT* transgenic mice. mAbs *12*, 1829334. https://doi.org/10.1080/19420862.2020.1829334.

CellPress OPEN ACCESS

STAR★METHODS

KEY RESOURCES TABLE

REAGENT or RESOURCE	SOURCE	IDENTIFIER
Antibodies		
Anti-ENPP1 antibody	Abcam	ab40003; RRID:AB_2099641
Anti-Vimentin antibody	Abcam	ab45939; RRID:AB_2257290
Anti-Cardiac Troponin I antibody	Abcam	ab47003; RRID:AB_869982
Anti-CD31 antibody	Abcam	ab7388; RRID:AB_305905
ENPP1 (L520) antibody	Cell Signaling Technology	2061S; RRID:AB_2231273
Anti-GAPDH Antibody	Millipore	ABS16; RRID:AB_10806772
PE anti-mouse ENPP1	BioLegend	149204; RRID:AB_2564620
Human ENPP-1 APC-conjugated Antibody	R&D Systems	FAB6136A; RRID:AB_3651408
APC anti-human ENPP3	BioLegend	324605; RRID:AB_756043
PE Mouse Anti-Human CD39	BD	555464; RRID:AB_395856
APC anti-human CD73	BioLegend	344003; RRID:AB_1877224
PE anti-human IgG Fc Antibody	BioLegend	410708; RRID:AB_2565786
CD11b- FITC (Clone: M1/70)	Thermo Fisher Scientific	11-0112-85; RRID:AB_464936
CD138- Biotin (Clone: 281-2)	BD Biosciences	553713; RRID:AB_394999
CD19⁻ Pacific Blue (Clone: 1D3)	ThermoFisher Scientific	48-0193-82; RRID:AB_2734905
CD21/CD35- FITC (Clone: 7G6)	BD Biosciences	553818; RRID:AB_395070
CD23⁻ APC (Clone: 2G8)	Southern Biotech	1585–11; RRID:AB_2794986
CD24(HAS)- PE/Cy7 (Clone: M1/69)	BD Biosciences	560536; RRID:AB_1727452
CD43⁻ APC (Clone: S7)	BD Biosciences	560663; RRID:AB_1727479
CD45R(B220)- PerCP-Cy5.5 (Clone: RA3-6B2)	ThermoFisher Scientific	45-0452-82; RRID:AB_1107006
CD5⁻ PerCP-Cy5.5 (Clone:53–7.3)	ThermoFisher Scientific	45-0051-82; RRID:AB_914334
CD93⁻ FITC (Clone: AA4.1)	ThermoFisher Scientific	11-5892-82; RRID:AB_465298
CD93⁻ PE-Cy7 (Clone: AA4.1)	ThermoFisher Scientific	25-5892-82; RRID:AB_469659
Fixable Viability Dye eFluor™ 780	ThermoFisher Scientific	65-0865-14
IgD- Brilliant Violet 605 (Clone: 11-26c.2a)	BioLegend	405727; RRID:AB_2562887
IgM- eFluor 450 (Clone: eB121-15F9)	ThermoFisher Scientific	48-5890-82; RRID:AB_10671539
IgM- PE (Polyclonal)	Southern Biotech	1020–09; RRID:AB_2794204
Ly-51(BP-1)- Biotin (Clone: 6C3)	BD Biosciences	553159; RRID:AB_394671
Purified CD16/32 (Clone: 93)	ThermoFisher Scientific	14-0161-86; RRID:AB_467135
Streptavidin- PE-Cy7	BD Biosciences	557598
Streptavidin-eFluor 450	Thermo Fisher Scientific	48-4317-82; RRID:AB_10359737
Chemicals, peptides, and recombinant proteins		
ENPP1 Human Recombinant	Prospec	ENZ-729
Human IgG	Sigma	I4506
ATP	Thermo Scientific	R0441
Fenestra HDVC agent	MediLumine	HDVC-121
Q5 high-fidelity DNA polymerase	New England Biolabs	M0491S
Critical commercial assays		
Cell titer-Glo	Promega	G7570
Gibson Assembly kit	New England Biolabs	E5510S
RNeasy Plus Micro Kit	Qiagen	74034
iScript cDNA Synthesis Kit	BioRad	1708890

(Continued on next page)

Continued

REAGENT or RESOURCE	SOURCE	IDENTIFIER
Von Kossa Stain Kit	StatLab	KTVKO
Deposited data		
Single nucleus RNA sequencing	This paper	GEO: GSE225826
Experimental models: Cell lines		
HEK293T	ATCC	CRL-3216
Experimental models: Organisms/strains		
Mouse: Humanized ENPP1 mouse	This paper	NA
Mouse: B6.Cg-*Fcgrt*^{tm1Dcr} Tg(FCGRT) 32Dcr/DcrJ	The Jackson Laboratory	014565
Oligonucleotides		
Primers for cloning, see Table S7 in method details	This paper	N/A
Primers for ECM genes, see Table S8 in method details	This paper	N/A
Primer: Human ENPP1 specific Forward: ACCTGGATTCAAGCATGGCA	This paper	N/A
Primer: Human ENPP1 specific Reverse: TGGGGTTTCTTGTGAAGGGG	This paper	N/A
Primer: Mouse ENPP1 specific Forward: ACAGCTTAATCTGACCACAGAA	This paper	N/A
Primer: human ENPP1 specific Reverse: TTTAGTCTCGGTGGCGTGAG	This paper	N/A
Recombinant DNA		
Human ENPP1	Horizon Discovery	MHS6278-202806113
Mouse ENPP1	Horizon Discovery	OMM5895-202525461
Rat ENPP1	GenScript	NM_053535.1
Pig ENPP1	GenScript	XM_021087933.1
Monkey Universal Reference CDNA	Zyagen	KD-UR-40
pHIV-EGFP	Addgene	#21373
Human ENPP3	R&D Systems	RDC2698
Human ENPP4	Horizon Discovery	MHS6278-202806522
Human ENPP5	Horizon Discovery	MHS6278-202807818
Human CD39	Horizon Discovery	MHS6278-202802580
Human CD73	Horizon Discovery	MHS6278-202759798
pIRES2-EGFP	Clontech	6029–1
pAcGFP1-C1	Clontech	632470
Software and algorithms		
Molecular Operating Environment (MOE)	Computing Group ULC	2022
ImageQuant software	GE healthcare,	Version 8.2
Amide software	Sourceforge	Version 1.0.6
ORS Dragonfly software	Object research systems	Version 2022.2
Prism	GraphPad	Version 9.0

EXPERIMENTAL MODEL AND STUDY PARTICIPANT DETAILS

Animal care and use

All animal experiments were conducted with the approval of the Animal Research Committee at the University of California, Los Angeles (UCLA). Animals were housed in the UCLA vivarium and cared for in compliance with the guidelines by the American Association for Accreditation of Laboratory Animal Care (AAALAC). Healthy male and female animals aged between 8 and 14 weeks were used in the study.

Generation of humanized ENPP1 mice

A humanized ENPP1 mouse was generated (C57BL/6NT background) by CRISPR/Cas-mediated genome engineering. The mouse Enpp1 gene (NCBI Reference Sequence: NM_001308327.1) is located on mouse chromosome 10. 25 exons have been identified. Similarly, the human ENPP1 gene (NCBI Reference Sequence: NM_006208.3) is located on human chromosome 6. 25 exons have been identified, with the ATG start codon in exon 1 and TGA stop codon in exon 25. To create the humanized ENPP1 mouse, the partial coding region of mouse ENPP1 exon 1 was replaced with the "human ENPP1 CDS-polyA" cassette. To engineer the donor vector, homology arms were generated by PCR using BAC clone as template. Cas9 and gRNA were co-injected into fertilized eggs along with the donor vector for mice production. The pups were genotyped by PCR.

Myocardial infarction and hENPP1mAb administration in humanized ENPP1 mice or ENPP1/Tg32 mice

Myocardial infarction (MI) was induced by ligation of the left anterior descending (LAD) coronary artery as previously described.[34] Adult humanized ENPP1 mice, both male and female were used for the study. hENPP1mAb (10 mg/kg, every 3 days) was administrated intraperitoneally, starting 3 days before myocardial infarction and continued twice weekly till Day 12 after MI for a total of 6 doses. Control animals were injected with polyclonal human IgG (Sigma, i4506) in an identical manner.

Humanized ENPP1/Tg32 animals (homozygous for all alleles) were similarly subjected to myocardial infarction and then injected intravenously a single loading dose of hENPP1mAb (100 mg/kg) or a single dose (100 mg/kg IV) of human polyclonal IgG. The single shot of the antibody is injected as soon as the animals recover from anesthesia and are able to move around in the cage.

Biodistribution studies of hENPP1mAb *in vivo*

Human IgG and hENPP1mAb were conjugated with *p*-Isothiocyanatobenzyl Desferrioxamine. 89Zr-oxalate was obtained from 3D Imaging LLC. On arrival, 89Zr-oxalate was diluted with 40% (v/v) 2 M Na_2CO_3 and allowed to incubate for 3 min. The activity was then diluted with a 2.5 times volume of 1 M N-(2-hydroxyethyl)piperazine-N-(2-ethanesulfonic acid) (pH 7.0). Final pH was checked with pHydrion plastic indicator strips (Micro Essential Laboratory) to confirm a pH of 7. IgG and hENPP1mAb conjugated protein were incubated for 1 h at room temperature at about 148–185 kBq/mg (4–5 mCi/mg). Radiolabeling efficiency was measured by instant thin-layer chromatography (ITLC) (Biodex Medical Systems) using 20 mM citrate buffer, pH 5.6, as the mobile phase with a Wizard 3″ 1480 Automatic Gamma Counter (PerkinElmer).

Humanized ENPP1 mice (14 weeks, male) were anesthetized with 2% vaporized isoflurane and injected via i.v. injection (tail vein) with 100 μL CT contrast agent Fenestra HDVC agent (MediLumine), followed by another i.v. injection of either Zr89-IgG antibody (n = 4) or radiolabeled Zr89-hENPP1mAb antibody (n = 4) at 0.5 mg/kg (110–120 μCi) in 100 μL. The rationale for using a dose of 0.5 mg/kg, rather than 10 or 100 mg/kg, is based on the tracer principle of PET imaging, which allows the visualization of biological and physiological processes with small amounts of specific radiopharmaceuticals.[35] A dose of 10 mg/kg or 100 mg/kg of the Zr89-labeled radioactive antibody would saturate the PET detector, as it is designed to be highly sensitive to tiny amounts of radiation. Each group was imaged with PET (energy window 350–650 keV) and CT (voltage 80kVP, current 150 μA, 720 projections, 200μm resolution) on a GNEXT PET/CT scanner (Sofie Biosciences, Dulles, VA).[36] MicroPET/CT images were acquired at 0.5h, 1h, 2h, 4h, 6h, 24h, 48h, 72h, 144h, and 240h post-injection (static PET, 10–40 min). The PET images were reconstructed using the 3D-OSEM/MAP algorithm (24 subsets and 3 iterates) with random, attenuation, and decay correction. The CT images were reconstructed using a Modified Feldkamp Algorithm. Amide software (Sourceforge, Version 1.0.6) was used to quantify PET signal intensity in organs in the co-registered PET/CT images.

METHOD DETAILS

Generation of humanized hENPP1mAb

An immunization protocol was initiated using the catalytic domain of human ectonucleotide pyrophosphatase/phosphodiesterase 1 (ENPP1) as the immunogen in BALB/c mice, employing Hock immunization method. Following immunization, mice were euthanized for lymph node extraction. The collected lymph nodes were pooled and subjected to B-cell enrichment followed by electrofusion to generate hybridomas.

Hybridomas were plated into 384-well plates and hybridoma supernatant screening was performed. The primary hybridoma screen was performed against human ENPP1 (1 μg/mL). This primary screening yielded 279 hybridomas that reacted against human ENPP1 and primary hits were confirmed and filtered through subsequent confirmation and counter-screening assays.

Further selection was performed using Fluorescence-Activated Cell Sorting (FACS) analysis with HEK293 cells expressing human ENPP1, utilizing culture supernatants to gauge antibody binding affinity. A total of 138 hybridomas exhibited a Mean Fluorescence Intensity (MFI) exceeding threshold, indicating high-affinity interaction with ENPP1.

An enzymatic inhibition assay specific to ENPP1 was then employed to assess the functional blockade capability of the antibodies produced by these hybridomas, leading to the selection of 8 top-performing clones for further characterization and cloning. Clone 12-J4 was identified as a lead candidate based on its enzymatic inhibition profile and was subsequently purified to obtain the monoclonal antibody.

The 12-J4 monoclonal antibody humanization process was performed in the following manner. Three humanized VH and three humanized VL sequences were designed with a T20 Analyzer score of humanness ranging from 84 to 98. A sequence liability

assessment was conducted for both VH and VL sequences to identify any residues that would be vulnerable to oxidation or aggregation. Following molecular construction and DNA scale-up, nine humanized variants utilizing the different VH and VL pairings were produced using a 10 mL TunaCHO 7-day process, followed by Protein A purification and buffer exchanging. CE-SDS analysis and endotoxin measurements (<1 EU/mg) confirmed quality, and intact mass QC by mass spectrometry showed expected molecular weights for the IgGs. The nine variants were confirmed to bind ENPP1 with KD values of less than 10 nM, showing minimal loss of affinity due to humanization. The murine antibody was of IgG1 isotype and a human IgG1 Fc was grafted onto the murine antibody.

One lead candidate (hENPP1mAb) was selected for detailed study based on similar biochemical and biophysical characteristics and with binding and potency properties similar to the original parent clone.

Homology modeling and protein-protein docking of hENPP1mAb with human ENPP1

During protein-protein docking, antibody and antigen are treated as rigid bodies therefore multiple conformations of the antibody and the antigen are required. Energetically feasible conformations of the antigen and the antibody were modeled from available crystal or cryoEM structures. In the case of the antibody, homology models were created since no crystal structures were available.

Modeling of the antigen

In the case of the antigen, five crystal structures were obtained from rcsb.org: 6wew, 6weu, 6wet and 6wev and each was aligned to chain cA of 6wfj. All ligands and hetero-groups were removed and the resulting protein structures prepared in MOE[37] using the standard structure preparation at pH 7.0; all hydrogens were added and any missing loops added.

Modeling of the antibody

Homology models of antibodies: Based on specific antibody sequence, 90 homology models were built as a light and heavy chain of the antibody. Crystal structures with $\geq$ 80% identity were found from rcsb.org. Each of the light and heavy chains of the crystal structures were aligned to the light and heavy chains of the original homology model. Next, all combinations of the light and heavy chains were created using MOE homology modeling.[37] The models of each chain were created together so that any energetically unfavorable conformations were eliminated.

Protein-protein docking

The protein-protein docking application in MOE 2022[38] was used for all docking studies. The process starts with a rigid-body sampling of possible complexes where each amino acid is represented as a bead. The search space was restrained with the hydrophobic patch potential. The antibody binding site was restricted to the 9 light-chain amino acids and the proximal heavy chain amino acids. The antibody docking was guided toward the enzyme active site, however large portions of the antigen structure were explored because of the radial restraint from the enzyme active site. That is, there was no requirement that the antibody occupies the active site. The enzyme active site was chosen near the enzyme active site based on the pdb ligand, SRA found in the ENPP1 crystal structure (pdbid: 6weu). The enzyme active site was defined based on the following amino acid residues [218, 255, 256, 257, 260, 276, 277, 290, 295, 321, 322, 323, 324, 325, 326, 340, 342, 371, 373, 376, 377, 380, 423, 424, 426, 528, 535]. The 90 antibody homology models were docked to each of the 5 antigen structures without the enzyme active site occupied.

Each final docked pose was scored by the molecular mechanics energy using the Amber10 forcefield (E) and a separated hydrophobic patch potential (S). Each antibody:antigen pair required approximately 30 min to complete. The set of 450 pairs were run with 2 sets of search parameters for a total of 900 simulations. After completing the runs the results were filtered by score (<-60) and energy (<-7000 kcal/mol). The filtering resulted in 97 and 59 structures in each of two sets of search parameters. Each set of structures were clustered based on proximity to the enzyme active site and the $C\alpha$- $C\alpha$ distances between heavy and light chain docked models.

Assessment of binding profile of hENPP1mAb using the Retrogenix membrane array

In the Retrogenix screen, 2 μg/mL of hENPP1mAb was screened for binding against human HEK293 cells, each cell line individually expressing one of 6101 full-length human plasma membrane proteins, secreted and cell surface-tethered human secreted proteins plus a further 396 human heterodimers. Initially, HEK293 cells were reverse-transfected with slides spotted with expression vectors for ZsGreen1 and human ENPP1 in both plasma membrane and secreted forms, alongside CD20 or EGFR. hENPP1mAb at various concentrations, a Rituximab biosimilar, or PBS were then applied post-fixation, with binding assessed using an Alexa Fluor 647 labeled anti-human IgG Fc detection antibody (AF647 anti-hIgG Fc). For library screening, 6101 expression vectors for a range of human proteins and 396 heterodimers were arrayed on slides, with ENPP1 mAb added post-cell fixation and binding detected similarly. This screening utilized duplicate slide-sets and ImageQuant software (GE healthcare, Version 8.2) for analysis, categorizing protein interactions based on signal intensity. The potential interactions alongside control vectors for CD20 (positive control) and EGFR (transfection and negative control), were expressed in HEK293 cells on new slides for confirmation screen. Slides were treated with 2 μg/mL of ENPP1 mAb, 1 μg/mL Rituximab biosimilar (array positive control) or no test molecule (secondary only; negative control). Binding to target-expressing cells and untransfected cells was again evaluated by fluorescence imaging. The assay was performed by Retrogenix labs, Charles River, UK.[39,40]

Potency curves of hENPP1mAb in inhibiting human ENPP1 catalytic activity

To generate concentration dependent potency curves, hENPP1mAb was serially diluted in assay buffer (50 mM Tris +2 mM $MgCl_2$ + 0.005% Tween 20 + 0.1% BSA). Diluted mAb was incubated with an equal volume of human ENPP1 protein (ProSpec, ENZ-729) for

30 min at room temperature in a 384 well plate. After 30 min, ATP (Thermo Scientific, R0441) was added to all the wells in the plate by washer dispenser and incubated at room temperature for 30 min. Cell titer-Glo (Promega, G7570) was then added to the plate and incubated for 10 min. The degree of luminescence in each well were detected by plate reader (BioTek, H1). The half maximal inhibitory concentrations (IC50) were determined using GraphPad Prism 9.

Plasmid construction and transfection

Full-length cDNA clones for human ENPP1 (BC059375) and mouse ENPP1 (BC160371) were acquired from Horizon Discovery, while cDNA clones for rat ENPP1 (NM_053535.1) and pig ENPP1 (XM_021087933.1) were obtained from GenScript. The cDNAs were amplified via PCR and cloned into XbaI-BamHI enzymatic sites of the pHIV-EGFP vector (Addgene #21373) using a Gibson Assembly kit (New England Biolabs). PCR amplifications utilized Q5 high-fidelity DNA polymerase (New England Biolabs), unless otherwise specified.

Monkey ENPP1 cDNA was cloned from Monkey Universal Reference cDNA (Zyagen) using Accuprime PCR (Invitrogen) for the upstream GC-rich region and Q5 PCR for the downstream region. A subsequent round of PCR was conducted to incorporate XbaI-BamHI sites for cloning into the pHIV-EGFP vector. An internal BstXI enzymatic site was utilized to ligate the two cDNA fragments to form the full-length cDNA.

MGC Sequence-Verified human cDNAs of ENPP4, ENPP5, CD39, CD73 (Horizon Discovery), and ENPP3 (Cat# RDC2698, R&D Systems) were cloned into the pIRES2-EGFP vector (Clontech) for expression studies. ENPP3 was transferred via NheI-XhoI enzymatic digestion and cloning, while ENPP4, ENPP5, CD39, and CD73 were PCR-amplified with primers incorporating NheI and XmaI enzymatic sites and cloned into NheI-XmaI sites of pIRES2-EGFP. Additionally, human ENPP1 in the pHIV-EGFP vector (XbaI-BamHI fragment) was cloned into NheI-BamHI sites of pIRES2-EGFP. ENPP1 and ENPP5 were further tagged with GFP at N- and C- terminus, respectively. ENPP1 (BstYI fragment from pIRES2-EGFP-ENPP1) was cloned into BglII site of pAcGFP-C1 (Clontech), while pIRES2-EGFP-ENPP5 was modified by PCR and subsequent bacterial *in vivo* assembly to remove ENPP5 stop codon and IRES2. Successful modification of the plasmids without error was confirmed by whole-plasmid sequencing. The cloning primers sequences are listed in Table S7.

PCR fidelity and sequence flanking restriction cloning sites were checked by Sanger sequencing to confirm no error in cDNA and correct plasmid assembly.

For expression studies, HEK293t cells were seeded in 6-well plates at a density of 5x10ˆ5 cells per well. One day after seeding, cells were transfected with 2 μg of the respective plasmids per well using FuGENE HD transfection reagent (Promega). Following transfection, cells were cultured for 48 h prior to subsequent assays.

Human ENPP3/human CD39/human CD73/human ENPP4/human ENPP5 overexpression verification

After human CD39 or human Cd73 plasmid transfection. the transfected HEK293t cells were incubated with anti-human ENPP3, anti-human CD39 or anti-human CD73 antibody for 30 min. After incubation, cells were resuspended in 1% BSA for flow cytometry (Thermo Fisher, USA). For human ENPP5 expression verification, cells expressing Human ENPP5 with fused EGFP were detected using the Nikon Eclipse Ti2 confocal microscopy (Nikon, USA) and flow cytometry.

hENPP1 mAb binding affinity/avidity

Subsequent to plasmid transfection, the transfected HEK293t cells were incubated with hENPP1mAb for 30 min. Following thorough washing, cells were incubated with PE anti Human IgG 2nd antibody for another 30 min. After incubation, cells were resuspended in 1% BSA for flow cytometry. BD LSRII flow cytometer was used for all flow cytometry experiments. Data was analyzed using Flowjo software. For the Kd value measurement, hENPP1mAb was serially diluted and incubated with an equal number of human ENPP1 overexpressing HEK cell. The Kd value was determined using GraphPad Prism 9.

RT-PCR and gel electrophoresis to distinguish mouse and human ENPP1mRNA sequence

Human ENPP1 and mouse ENPP1 specific primers were designed for distinguishing murine and human mRNA sequences in the humanized ENPP1 mice. Human ENPP1 specific primer: ACCTGGATTCAAGCATGGCA (F)/TGGGGTTTCTTGTGAAGGGG (R), product size 206 bp. Mouse ENPP1 specific primer: ACAGCTTAATCTGACCACAGAA (F)/TTTAGTCTCGGTGGCGTGAG (R), product size 318 bp, were used. Total RNA was extracted from heart tissue of C57BL/6J wild type mice or humanized ENPP1 mice. The synthesized cDNA was obtained using the iScript cDNA Synthesis Kit (BioRad, 1708890). The human/mouse mixed primers were used to amplify the cDNA of C57BL/6J wild type mice or humanized ENPP1 mice separately. Amplified products were then subjected to agarose gel electrophoresis.

RNA extraction and Q-PCR

For RNA extraction from heart tissue, both the uninjured and injured regions were collected and homogenized in lysis buffer. Subsequently, RNA extraction was carried out using the RNeasy Plus Micro Kit (Qiagen, 74034). The synthesized cDNA was obtained using the iScript cDNA Synthesis Kit (BioRad, 1708890), followed by qPCR analysis. Human ENPP1 and mouse ENPP1 specific primers were used for measuring the human or mouse ENPP1 gene expression in the heart tissue of humanized ENPP1 mice.

To Determine the extracellular matrix gene expression in the injured heart. The injured regions of IgG or hENPP1mAb treated animals were collected at 3 days or 7 days post MI. 5 ECM genes were measured. The ECM gene primers sequences are listed in Table S8.

Histology studies

Animals were euthanized before necropsy, and the heart, liver, kidney, lung and spleen harvested and fixed in 4% paraformaldehyde in PBS at 4°C overnight. The fixed hearts were then dehydrated in 10% and 30% sucrose solutions. Subsequently, the hearts were snap frozen in Tissue-Tek O.C.T compound (SAKURA Finetek, 4583) and sectioned at a thickness of 10μm. For the immunohistochemical staining, the fixed organs were embedding in paraffin section for histological analysis.

For immunofluorescent staining, the tissue sections were post-fixed in 4% paraformaldehyde for 10 min, followed by permeabilization in 0.1% Triton X-100 for 15 min. Subsequently, the sections were blocked in 10% species-specific normal serum in 1% BSA/PBS for 1 h. The primary antibodies, diluted in 1% BSA/PBS, were then incubated with the sections overnight at 4°C. Afterward, the sections were incubated with the secondary antibodies diluted in PBS for 1 h. Samples were mounted by antifade media (Vector lab, H-1500). The images were captured using the Nikon Eclipse Ti2 confocal microscopy (Nikon, USA) and analyzed using NIS Element AR software (Nikon) or ImageJ. To determine myocyte size in ENPP1CKO mice post-injury, the cross-sectional area of 100 cardiac myocytes in the injured region was measured for each mouse. CD31 staining was utilized to measure capillary density.[41] 6 random areas from each injured region of heart section were counted the number of capillaries, then expressing the data as capillaries/mm^2.

For immunohistochemical staining, the sections were stained with hematoxylin and eosin (Fisher Chemical, SE23-500D) or Masson Trichrome Staining (Thermo Scientific, 87019). The stained sections were scanned using the Aperio AT2 (Leica, Germany). The fibrotic area was analyzed in heart sections from the apex to the mid-ventricle. The scar tissue area was calculated as the fraction of the left ventricular surface area occupied by the scar tissue.

For Von Kossa staining to determine ectopic calcification in different organs, aorta, heart, kidney, and aortic valves were stained by Von Kossa Stain Kit (Statlab, KTVKO).

Immunoblotting

Uninjured and injured heart tissue were harvest at 3 days, 7 days and 14 days after myocardial infarction. The tissue samples were homogenized in RIPA buffer (Thermo Scientific, 89900) with protease and phosphatase inhibitor cocktails (Thermo Scientific, 78442). Protein was quantified by BCA assay (Thermo Scientific, 78442) and equal amount of proteins were loaded on 4%–12% Tris-Glycine precast gels (Thermo Scientific, XP04125BOX) for Western blot analysis. The proteins were transferred to PVDF membranes, which were then incubated overnight with primary antibodies. The membranes were treated with a secondary antibody conjugated with horseradish peroxidase, followed by ECL substrate (Thermo Scientific, 32209), and chemoluminescent signal detected by western blot imaging system (Cytiva, IQ600).

Echocardiography

Baseline echocardiography was conducted prior to injury, followed by evaluations at 1, 2, and 4 weeks post-myocardial infarction. During echocardiography, the animals were anesthetized using a combination of 1.5% isoflurane and 95% oxygen. Short/long axis B-mode and M-mode images were acquired using the Vevo-3100 imaging system and an mx400 transducer from Visual Sonics. Vevo Lab software was utilized for all measurements and analysis.

Ectonucleotidase assay of injured heart tissue

At day 7 post MI, uninjured and injured regions of hearts from IgG treated or hENPP1mAb-treated mice were harvested and homogenized using assay buffer (50 mM Tris +2 mM MgCl2 + 0.005% Tween 20 + 0.1% BSA) containing protease and phosphatase inhibitor cocktails. The lysate was kept on ice for 30 min and then centrifuged at 3000 xg, 4°C to remove debris. Next, the lysate was diluted and passed through a 3 kD Spin Column (Millipore, UFC900324) to retain the protein fraction. The protein-rich lysate was normalized to protein concentration, and then incubated with ATP (2 μM) for 2 h at room temperature. The residual ATP level was measured using the Cell Titer-Glo luminescent Assay (Promega, G7572).

In vivo microCT imaging

Bone density micro-CT

IgG and hENPP1mAb treated Enpp1 humanized mice were anesthetized with 2% vaporized isoflurane. Animals were imaged with high-resolution gated Cardiac CT on a micro CT scanner developed by the Crump Institute for molecular imaging at UCLA (CrumpCAT).[42] The bone density was analyzed using AMIDE software (Sourceforge, Version 1.0.6).

Retrospective cardiac CT gating methodologies

IgG and hENPP1mAb treated Enpp1 humanized mice were anesthetized with 2% vaporized isoflurane and injected via i.v. injection (tail vein) with 100 μL CT contrast agent Vivovist agent (Nanoprobes). Animals were imaged at 200 micron spatial resolution on the CrumpCAT scanner, which employs our proprietary retrospective cardiac gating method. Mouse scans are performed using the acquisition parameters shown in Table S9. Once acquisition is complete, the retrospective "gating" software extracts respiratory

and cardiac signals directly from projection data with no need for special hardware (electrodes, cushions, etc). Time series (signals) are extracted by summing pixel intensity values inside a region for every projection.

For the respiratory signal, the region includes the lower part of the thoracic cavity and the diaphragm. For the cardiac signal, the region includes the left atrium and the upper part of the thoracic cavity. Regions are automatically identified by co-registering a mouse thorax template onto a projection and by maximizing Mutual Information. Cardiac frequencies of up to 600 bpm can be detected with this method. Projections corresponding to the inspiration phase of the respiratory cycle are rejected to eliminate motion blurring. The remaining projections (~70%) are assigned a percentage value representing the fraction of the cardiac cycle to which they belong. They are then grouped into 12 phases, according to their assigned cycle percentage: first phase groups projections with assigned cycle percentage from 0 to 8.33%, second phase: 8.33%–16.67%, and so on.

The duration of a phase is therefore equal to 1/12 of the average cardiac cycle length. For example, at 400 bpm the average cardiac cycle length is 150 ms and the average phase duration is 12.5 ms. It is worth mentioning that both the respiratory and cardiac cycles can vary during the scan as the method is insensitive to such variations.

Phase datasets contain between 1000 and 1400 projections and are reconstructed individually according to the parameters in Table S9. Contrary to back-projection methods, iterative reconstruction produces consistent image levels across phases, regardless of the number of projections in each phase. The application of post-reconstruction filters helps with noise reduction.

Images were analyzed using AMIDE software (Sourceforge, Version 1.0.6). 3D Rendering was performed by ORS Dragonfly software (Object research systems, Version 2022.2).

Single nuclei RNA sequencing

Single nuclei isolation

Sham (uninjured), IgG or hENPP1mAb treated Mice hearts were harvest at 7 days after MI. The injured or sham uninjured heart tissue was dissected and homogenized using nuclei lysis buffer. Nuclei pellet was resuspended and filtered through 100μm and 20μm Cell Strainer (43-50000-98, PluriStrainer). Nuclei concentrations were counted using Countess II Automated Cell Counter (Thermo Fisher Scientific).

Library preparation and sequencing

Single nuclei gene expression libraries were created using Chromium Next GEM Single ‘ell 3’ (v3.1 Chemistry) (PN-1000121, 10x Genomics), Chromium Next GEM Chip G Single Cell Kit (PN-1000127, 10x Genomics), and Single Index Kit T Set A (PN-1000213, 10x Genomics) according to the manufacturer’s instructions. Briefly, nuclei were loaded to target 10,000 nuclei to form GEMs and barcode individual nuclei into cDNA libraries. Library quality was assessed using 4200 TapeStation System and D1000 ScreenTape (Agilent) and Qubit 2.0 (Invitrogen) for concentration and size distribution. Samples were sequenced using NovaSeq6000 (Illumina) using Paired End 2X100 cycles (28 + 10+10 + 90). 200 million reads were targeted for each sample, targeting 20,000 reads per nuclei.

Single nucleus sequencing data analysis

Preprocessing of the raw data was conducted following the Cell Ranger pipeline (10X Genomics). The Cellranger output expression matrices were merged for each sample. The R package Seurat (v4.3.0) was used to cluster the cells in the merged matrix. Cells with less than 100 genes or more than 1e4 transcripts or 1% of mitochondrial expression were first filtered out as low-quality cells for the snRNA-seq samples. The NormalizeData function was used to normalize the expression level for each cell with default parameters. The FindVariableFeatures function was used to select variable genes with default parameters. The ScaleData function was used to scale and center the counts in the dataset. Principal component analysis (PCA) was performed on the variable genes. The RunHarmony function from the Harmony package was applied to remove potential batch effect among samples processed in different batches. Uniform Manifold Approximation and Projection (UMAP) dimensional reduction was performed using the RunUMAP function. The clusters were obtained using the FindNeighbors and FindClusters functions with the resolution set to 0.6. The cluster marker genes were found using the FindAllMarkers function. The cell types were annotated by overlapping the cluster markers with the published marker genes. The dot plot was plotted using the DotPlot function. The violin plots were plotted using the VlnPlot function. Differential expression analysis between two group of cells was conducted using the FindMarkers function. Genes with adjusted p value smaller than 0.05 were considered significantly differentially expressed. Enrichr was used for pathway enrichment analysis on the differentially expressed genes.

In vivo toxicity test

To determine any potential toxicity associated with the hENPP1mAb, the IgG or hENPP1mAb were administered to the humanized ENPP1 animals, every 3 days for 2 weeks (10 mg/kg), Animal were euthanized at 4 weeks for blood test and different organs histology diagnosis. To determine the acute toxicity of hENPP1mAb administration, a single dose was (100 mg/kg) injected into the animal, and the animals were harvested at 3 days after the injection for complete blood count and serum biochemistry.

Complete blood count and serum biochemistry

Peripheral blood was obtained via cardiac puncture. Whole blood or serum was collected in heparinized tubes (365965, BD Biosciences) or in serum tubes (365963, BD Biosciences). 250μL whole blood was used for determining complete/differential blood counts and 120μL serum was used for biochemistry analysis. Both Complete blood count and serum biochemistry were tested by IDEXX Bioanalytic (IDEXX).

B cell function analysis

To measure the B cell function after the hENPP1mAb treatment, a single dose of IgG control or hENPP1mAb at 100 mg/kg dose was administered to humanized ENPP1/Tg32 mice and then the animals were harvested at 7 days. Animal blood, bone marrow and spleen were collected for flow cytometry assay.[43,44] All staining procedures were performed in cold Ca++Mg++ free PBS. Samples were first incubated with a CD16/32 antibody to block non-specific binding of antibodies to cells via Fc receptors. All antibodies utilized are listed in the key resources table. For surface staining, cells were incubated on ice for 30 min with the appropriate dilution of antibodies. Unbound antibodies were washed from cells with cold Ca++Mg++ free PBS. To be able to exclude dead cells during analyses of samples for intracellular staining, cells were first pre-incubated with the Fixable Viability Dye eFluorTM 780 (ThermoFisher) as recommended by the manufacturer protocol. Subsequently, the cells were stained for surface antigens. Samples were run on an LSRII (BD Biosciences) flow cytometer. Frequencies of cell populations were determined using the FlowJo Software v10 samples.

Metabolomics

To conduct a metabolomic analysis on the hearts of both IgG and hENPP1mAb-treated animals following cardiac injury, 10 mg of injured tissue was homogenized in 1 mL of 80% methanol. The homogenate was then incubated on dry ice for 30 min. Following incubation, the homogenate underwent vertexing and subsequent centrifugation at 15,000x g for 15 min at 4°C. The supernatant was carefully transferred to a glass vial and subjected to vacuum drying in preparation for LC/MS analysis.

Dried metabolites were resuspended in 50% ACN:water and an aliquot was loaded onto a Luna 3 μm NH2 100 A (150 × 2.0 mm) column (Phenomenex). The chromatographic separation was performed on a Vanquish Flex (Thermo Scientific) with mobile phases A (5 mM NH4AcO pH 9.9) and B (ACN) and a flow rate of 200 μL/min. A linear gradient from 15% A to 95% A over 18 min was followed by 7 min isocratic flow at 95% A and re-equilibration to 15% A. Metabolites were detected with a Thermo Scientific Q Exactive mass spectrometer run with polarity switching (+3.5/−3.5 kV) in full scan mode with an m/z range of 70–975. The open source Maven (v 8.1.27.11) application was used to quantify the targeted metabolites by area under the curve using expected retention time and accurate mass measurements (<5 ppm). Data analysis was performed using in-house R scripts (https://github.com/graeberlab-ucla/MetabR).

Seahorse respiration assay

Injured regions of hearts from IgG or hENPP1mAb treated mice were harvested at day 3 post MI and stored in −80°C until prepared for the Seahorse experiments. Frozen tissues were thawed on ice and homogenized in 250 μL MAS buffer (70 mM sucrose, 220 mM mannitol, 5 mM KH2PO4, 5 mM MgCl2, 1 mM EGTA, 2 mM HEPES pH 7.2) with a bead homogenizer.[45] A BeadBlaster (Benchmark Scientific) was set to homogenize with 2 cycles of 30 s on, 30 s off at 6.5 m/s. The beads were washed with 50 μL MAS to recover the remaining homogenate. Homogenates were centrifuged at 500 x g and the supernatant was collected for testing. Protein concentrations were determined by BCA assay kit (Thermo Fisher). Homogenates were loaded into Seahorse XF96 microplate at 1.5 μg/well in MAS buffer (20 μL each well) and centrifuged at 2,000×g for 5 min at 4°C. After centrifugation the volume was increased to 150μL by adding 130μL MAS containing cytochrome *c* (10 μg/mL). Substrate injections at port A included final concentrations of 1 mM NADH to determine the respiratory capacity of mitochondrial Complex I or 5 mM succinate with 2 μM rotenone to determine the respiratory capacity of mitochondrial Complex II. The following compounds were injected sequentially to final concentration of 2 μM rotenone with 4 μM antimycin A (Port B); 0.5 mM TMPD with 1 mM ascorbic acid (Port C); and 50 mM azide (Port D). OCR rates were measured using Seahorse XF96 Extracellular Flux Analyzer (Agilent Technologies) and normalized to protein or mitochondrial content quantified by MitoTracker Deep Red (MTDR). For MTDR normalization, 0.375 μg/well of homogenate was stained with 500 nM MTDR for 10 min followed by two wash steps to remove the dye (Thermo Fisher). MTDR fluorescence was read on a Tecan Spark plate readers (Ex: 633 nm; Em: 678 nm).

Pharmacokinetic analysis of hENPP1mAb in Tg32 homozygous mice

7 week old male B6.Cg-Fcgrttm1Dcr Tg(FCGRT)32Dcr/DcrJ (Tg32, JAX stock# 014565) mice, homozygous for the Tg(FCGRT)32Dcr human FcRn transgene, were used in the pharmacokinetic study. On Day 0, body weights were measured, and hENPP1mAb (10 mg/kg) or a human IgG1 chimeric antibody HuLys11 at an identical dose were administered intravenously to mice. 25 μL blood samples were collected from each mouse at 1, 3, 7, 10, 14, 17, 21, and 28 days. The blood samples were collected into 1 μL K3EDTA, processed to plasma, and stored at −20°C. A human IgG ELISA (Mabtech 3850-1AD-6) chosen for its high sensitivity for HuLys11 was used to measure human IgG (surrogate for hENPP1mAb) in peripheral blood samples. The PK assay and half-life calculation were performed by the Jackson Laboratory using standard algorithms.[46]

QUANTIFICATION AND STATISTICAL ANALYSIS

All data is presented as mean ± standard error of the mean (SEM), and the value of n represents biological replicates. Statistical analysis was performed using GraphPad (Prism). Specific tests are mentioned in figure legends. A p value < 0.05 was considered as statistically significant.

Cell Reports Medicine, Volume *5*

Supplemental information

A humanized monoclonal antibody targeting an ectonucleotidase rescues cardiac metabolism and heart function after myocardial infarction

Shen Li, Bo Tao, Jijun Wan, Enca Montecino-Rodriguez, Ping Wang, Feiyang Ma, Baiming Sun, Yiqian Gu, Sivakumar Ramadoss, Lianjiu Su, Qihao Sun, Johanna Ten Hoeve, Linsey Stiles, Jeffrey Collins, R. Michael van Dam, Mikayla Tamboline, Richard Taschereau, Orian Shirihai, Douglas B. Kitchen, Matteo Pellegrini, Thomas Graeber, Kenneth Dorshkind, Shili Xu, and Arjun Deb

Supplementary Materials for

A Humanized Monoclonal Antibody Targeting an Ectonucleotidase Rescues Cardiac Metabolism and Heart Function after Myocardial Infarction

Shen Li[1,2,3,4,5,6], Bo Tao[1,2,3,4,5,6], Jijun Wan[1,2,3,4,5,6], Enca Montecino-Rodriguez[13], Ping Wang[1,2,3,4,5,6], Feiyang Ma[7], Baiming Sun[1,2,3,4,5,6],Yiqian Gu[3,4,5], Sivakumar Ramadoss[1,2,3,4,5,6], Lianjiu Su[1,2,3,4,5,6], Qihao Sun[1,2,3,4,5,6], Johanna Ten Hoeve[6,8,9,10], Linsey Stiles[6,11], Jeffrey Collins[6,9,10], R. Michael van Dam[6,9,10], Mikayla Tamboline[6,9,10], Richard Taschereau[6,9,10],Orian Shirihai[6,11], Douglas B. Kitchen[12], Matteo Pellegrini[3,4,5], Thomas Graeber[6,8,9,10], Kenneth Dorshkind[13], Shili Xu[6,9,10,14], Arjun Deb*[1,2,3,4,5,6]

*Address Correspondence to: adeb@mednet.ucla.edu

The following is included in the supplementary material.

Fig. S1 to S10
Tables S1-S9

Fig. S1

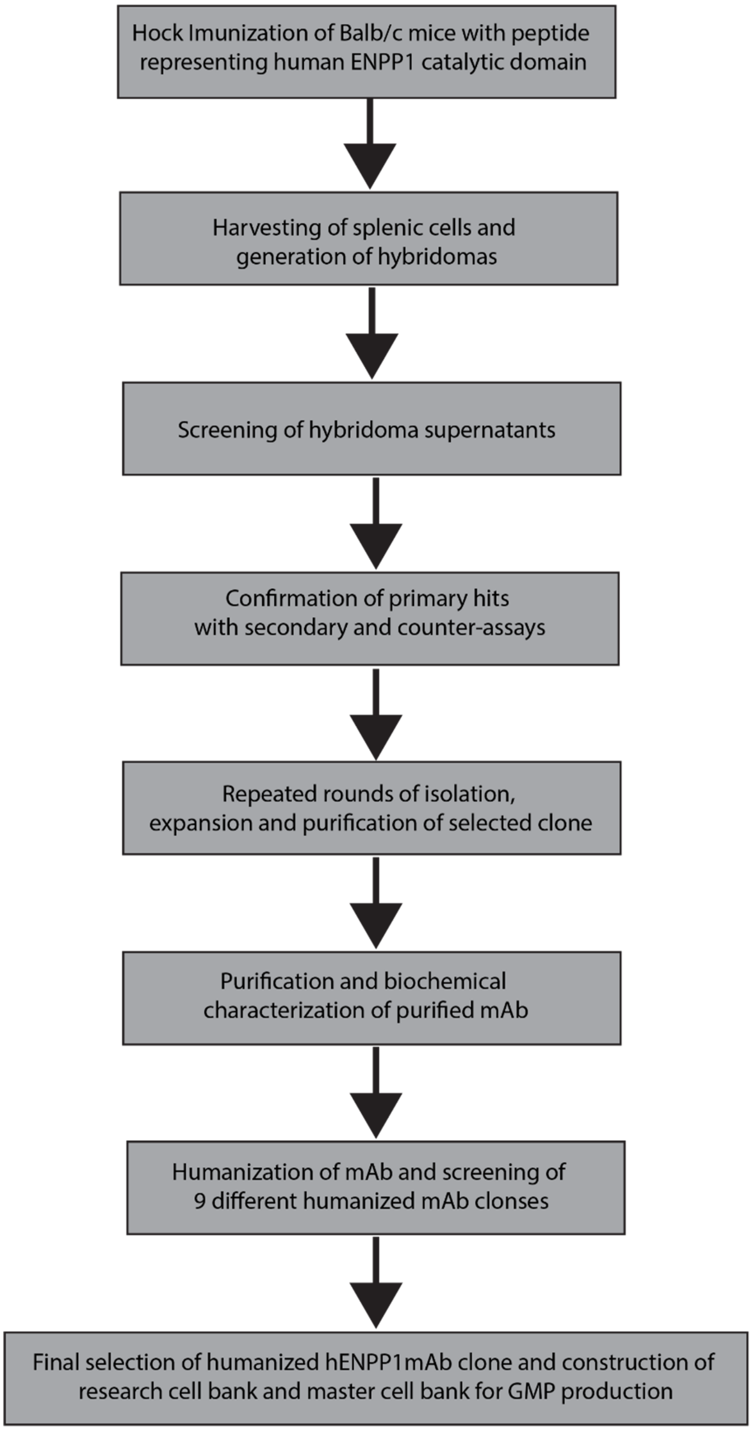

Fig S1. Schematic diagram of hENPP1mAb generation. Related to Figure 1. Schematic diagram of steps followed to generate a humanized ENPP1mAb. A peptide representing the human ENPP1 catalytic domain (generated in a mammalian cell line) was injected into Balb/c mice to immunize mice, followed by isolation of splenic cells and generation of hybridomas, screening of hybridoma supernatants and selection and expansion of clones, purification of mouse monoclonal antibody, followed by rounds of further cloning, and then full humanization of antibody, followed by screening of humanized clones and final selection of hENPP1mAb for research cell and master cell bank production.

Fig. S2

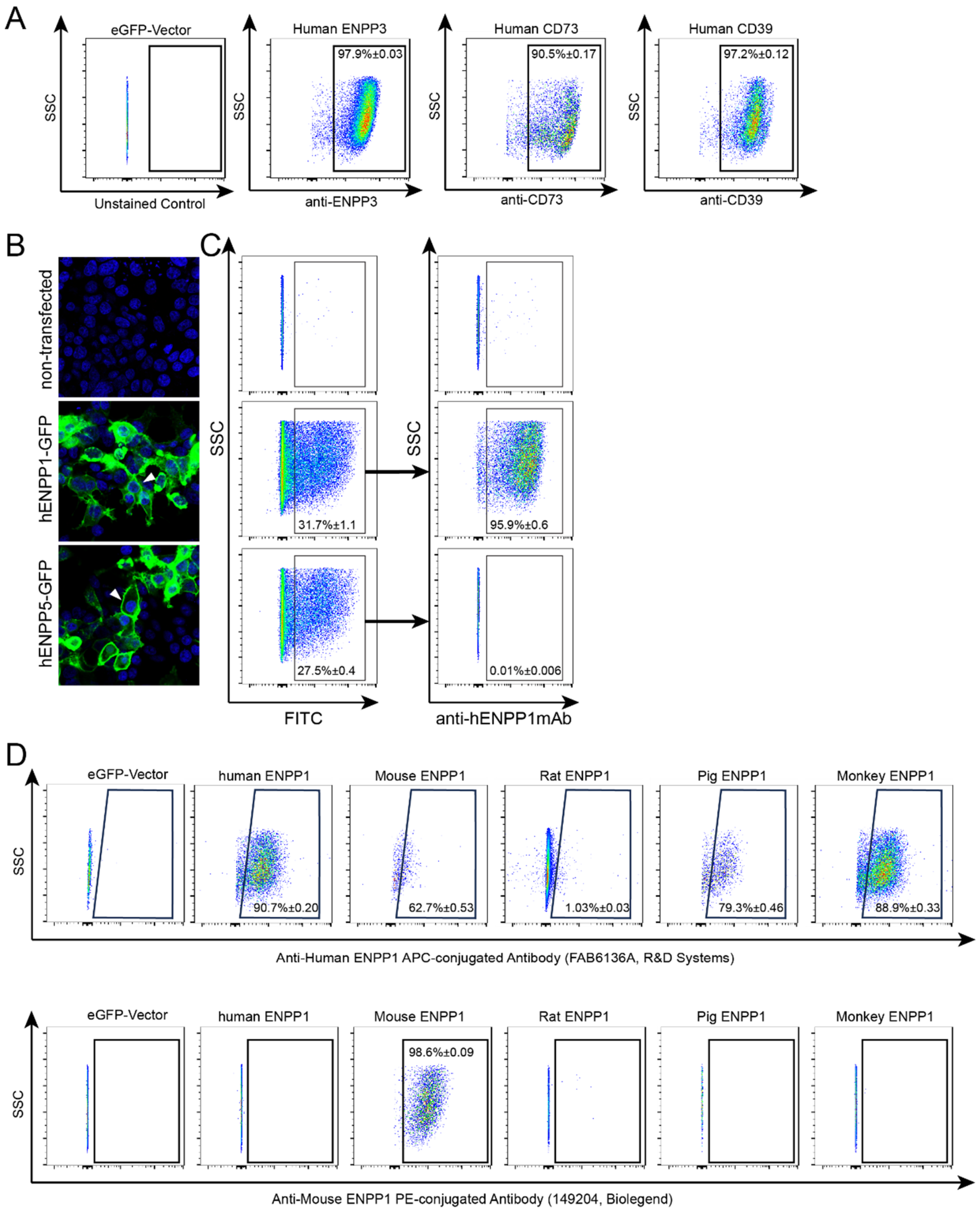
A
eGFP-Vector
Human ENPP3
Human CD73
Human CD39
97.9%±0.03
90.5%±0.17
97.2%±0.12
SSC
Unstained Control
anti-ENPP3
anti-CD73
anti-CD39
B
non-transfected
hENPP1-GFP
hENPP5-GFP
C
SSC
31.7%±1.1
27.5%±0.4
95.9%±0.6
0.01%±0.006
FITC
anti-hENPP1mAb
D
eGFP-Vector
human ENPP1
Mouse ENPP1
Rat ENPP1
Pig ENPP1
Monkey ENPP1
90.7%±0.20
62.7%±0.53
1.03%±0.03
79.3%±0.46
88.9%±0.33
Anti-Human ENPP1 APC-conjugated Antibody (FAB6136A, R&D Systems)
98.6%±0.09
Anti-Mouse ENPP1 PE-conjugated Antibody (149204, Biolegend)

Fig S2. Verification the overexpression of different targets in HEK cell line. Related to Figure 1 and 2. **(A)** Commercial anti-ENPP3, anti-Cd73, and anti-CD39 antibodies were used to detect corresponding antigens in HEK cells over-expressing them. (n=3). **(B)** hENPP1-GFP and hENPP5-GFP fusion proteins were expressed in HEK cells. Left panel demonstrates surface expression of fused hENPP1-GFP and hENPP5-GFP on cell surface (arrowheads) compared to non-transfected cells. **(C)** Flow cytometry to determine expression of GFP and binding to hENPP1mAb. FITC expression in hENPP1-GFP and hENPP5-GFP expressing HEK cells, with unstained cells used for gating. hENPP1mAb binds to hENPP1-GFP fused protein but does not bind to hENPP5-GFP. **(D)** Flow cytometry demonstrating binding of species specific ENPP1 to either commercially available anti-human ENPP1 antibody or anti-mouse ENPP1 antibody. Species specific ENPP1 was cloned and over-expressed in HEK cells (n=3) The anti-human antibody reacts against human, pig and monkey ENPP1. A mouse specific antibody is used to demonstrate mouse specific ENPP1 expression**.**

Fig S3

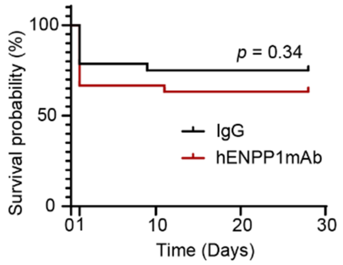

Fig S3. Kaplan Meier curves of survival after MI in the IgG and hENPP1mAb injected animals (n=28 in IgG, n=30 in hENPP1mAb). Related to Figure 3 and 4.

Fig. S4

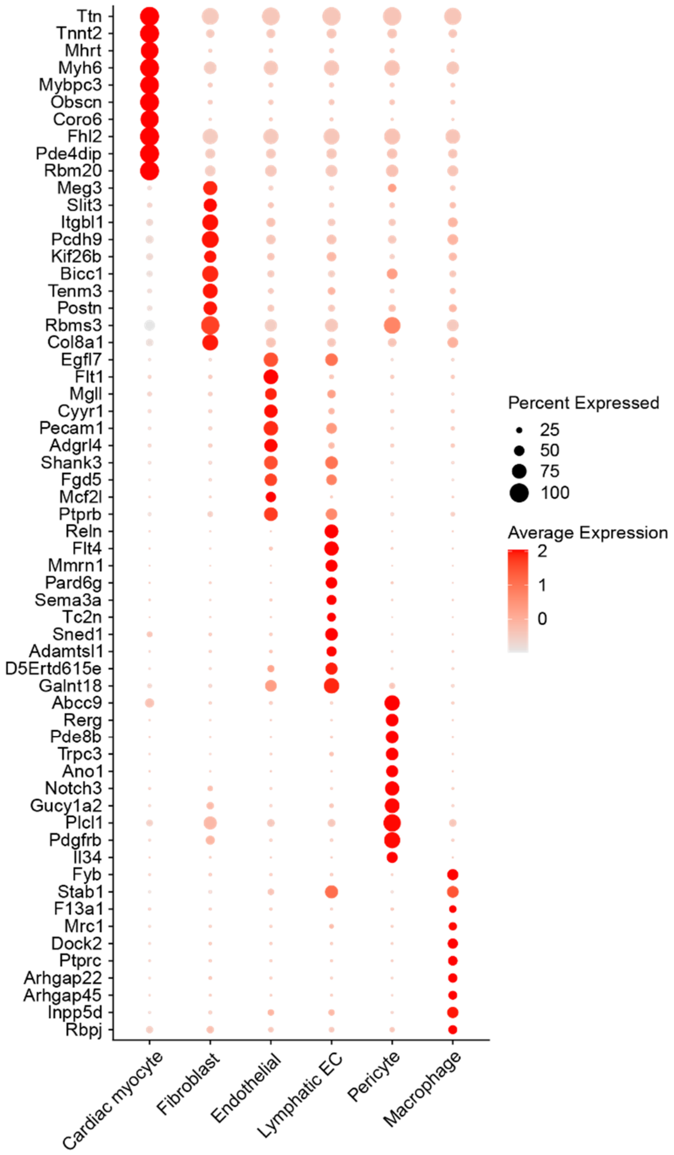

Fig S4. Single-nuclei RNA sequencing of hearts of IgG and hENPP1mAb treated animals at 7 days post MI. Related to Figure 5. Canonical genes used to characterize various cell population in the infarcted

Fig. S5

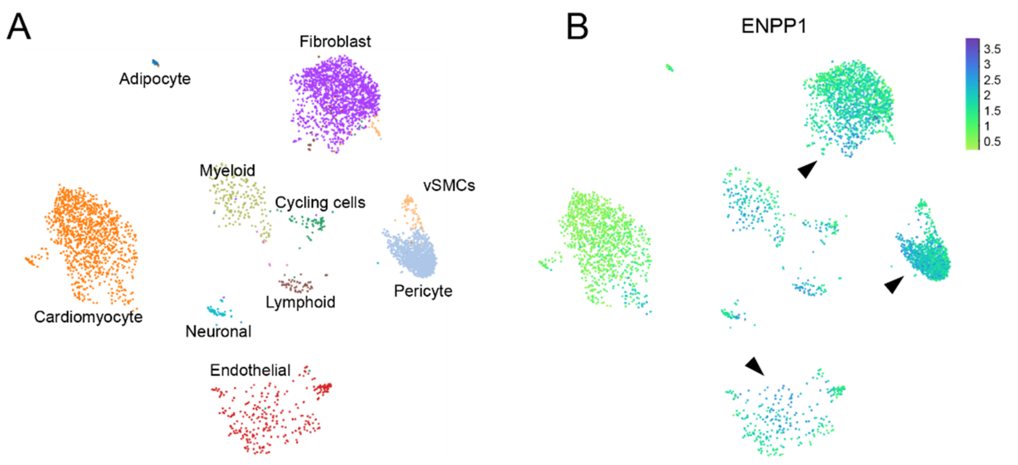

Fig S5. ENPP1 expression in different cell clusters in the infarcted human heart. Related to Figure 5. **(A)** Uniform manifold approximation and projection (UMAP) of snRNA-seq data of infarcted human heart samples. **(B)** Analysis of ENPP1 expression across cell populations. ENPP1 is observed to be expressed (arrowheads) predominantly in non-myocyte population of cardiac tissue in infarcted human hearts. (n=23 patients, Sampling time points, 2-166 days after infarction.) (ENPP1 expression data analyzed from published data sets in Kuppe, Christoph et al. "Spatial multi-omic map of human myocardial infarction." Nature Vol. 608,7924 (2022): 766-777. doi:10.1038/s41586-022-05060-x)

Fig. S6

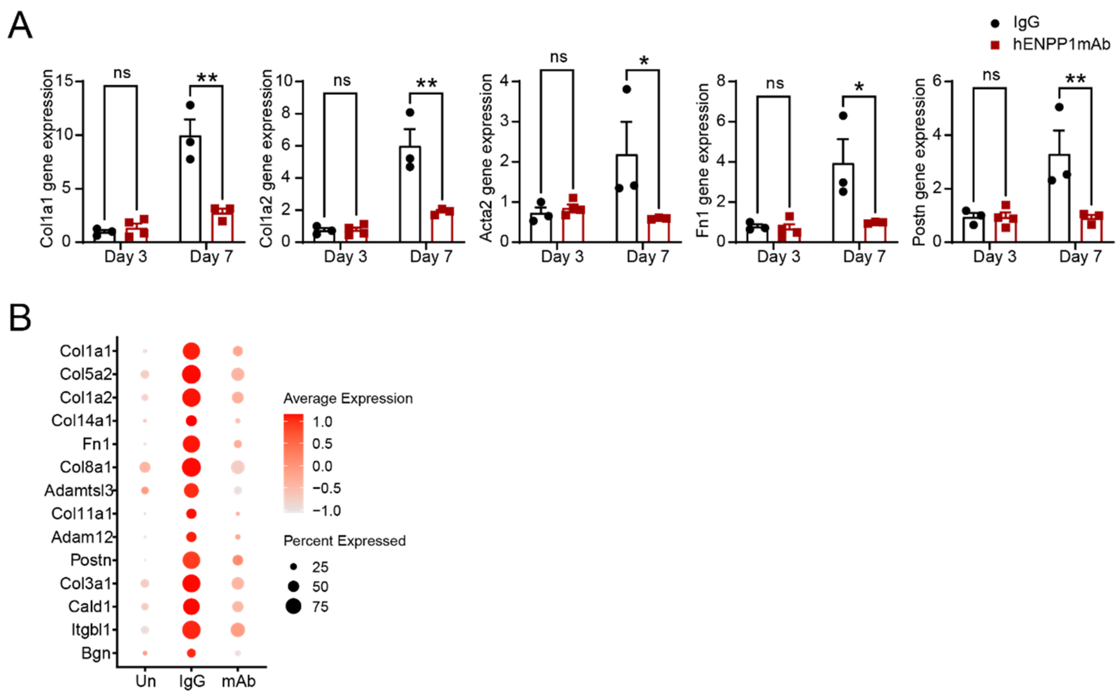

Fig S6. Extracellular matrix and myofibroblast gene expression in IgG and hENPP1mAb treated animals after MI. Related to Figure 5. (A) qPCR on injured region of hearts of IgG and hENPP1mAb treated animals at Day 3 and Day 7 following MI. (n=3 in Day 3 IgG, Day 7 IgG, and Day 7 hENPP1mAb; n=4 in Day 3 mAb). **(B)** Expression of ECM and myofibroblast genes in hearts of IgG and hENPP1mAb injected animals (Day 7 MI) compared to sham injured animals. Data represented as mean ± S.E.M., **p<0.01, *p<0.05, ns: not significant, Statistical significance was determined using Unpaired multiple t-test in **(A)**.

Fig. S7

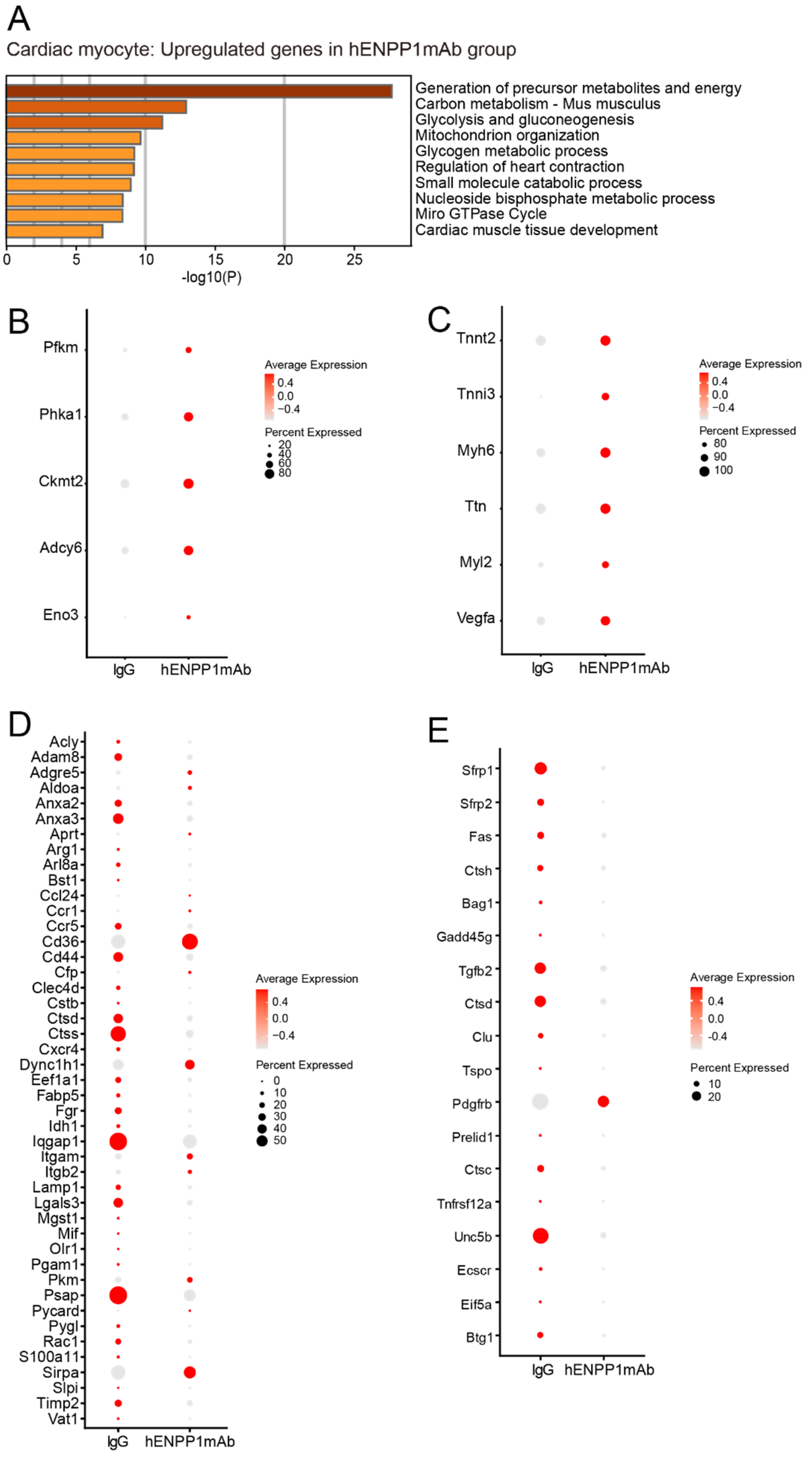
A
Cardiac myocyte: Upregulated genes in hENPP1mAb group
Generation of precursor metabolites and energy
Carbon metabolism - Mus musculus
Glycolysis and gluconeogenesis
Mitochondrion organization
Glycogen metabolic process
Regulation of heart contraction
Small molecule catabolic process
Nucleoside bisphosphate metabolic process
Miro GTPase Cycle
Cardiac muscle tissue development
0 5 10 15 20 25
-log10(P)
B
Pfkm
Phka1
Ckmt2
Adcy6
Eno3
IgG hENPP1mAb
Average Expression
0.4
0.0
−0.4
Percent Expressed
20
40
60
80
C
Tnnt2
Tnni3
Myh6
Ttn
Myl2
Vegfa
IgG hENPP1mAb
Average Expression
0.4
0.0
−0.4
Percent Expressed
80
90
100
D
Acly
Adam8
Adgre5
Aldoa
Anxa2
Anxa3
Aprt
Arg1
Arl8a
Bst1
Ccl24
Ccr1
Ccr5
Cd36
Cd44
Cfp
Clec4d
Cstb
Ctsd
Ctss
Cxcr4
Dync1h1
Eef1a1
Fabp5
Fgr
Idh1
Iqgap1
Itgam
Itgb2
Lamp1
Lgals3
Mgst1
Mif
Olr1
Pgam1
Pkm
Psap
Pycard
Pygl
Rac1
S100a11
Sirpa
Slpi
Timp2
Vat1
IgG hENPP1mAb
Average Expression
0.4
0.0
−0.4
Percent Expressed
0
10
20
30
40
50
E
Sfrp1
Sfrp2
Fas
Ctsh
Bag1
Gadd45g
Tgfb2
Ctsd
Clu
Tspo
Pdgfrb
Prelid1
Ctsc
Tnfrsf12a
Unc5b
Ecscr
Eif5a
Btg1
IgG hENPP1mAb
Average Expression
0.4
0.0
-0.4
Percent Expressed
10
20

Fig S7. Single Nuclei RNA-Seq demonstrating transcriptomic changes in myocytes and non-myocyte populations in hearts of IgG and hENPP1mAb treated animals at Day 7 post MI. Related to Figure 5. **(A)** GO analysis of main pathways differentially upregulated in cardiac myocytes in hENPP1mAb treated versus IgG control animals. **(B)** Dot plot demonstrating differential upregulation in myocytes of genes regulating glycolysis in hENPP1mAb injected animals. **(C)** Dot plot demonstrating differential upregulation of genes encoding for contractile proteins in hENPP1mAb injected animals. **(D)** Dot plot demonstrating the expression of inflammatory genes in macrophages of IgG versus hENPP1mAb injected animals. **(E)** Dot blot representing gene expression of pro-apoptotic genes across myocyte and non-myocyte cell populations of IgG versus hENPP1mAb injected animals. ($p<0.05$ for all genes shown differentially expressed, n=3/group for single nuclear transcriptomics)

Figure S8

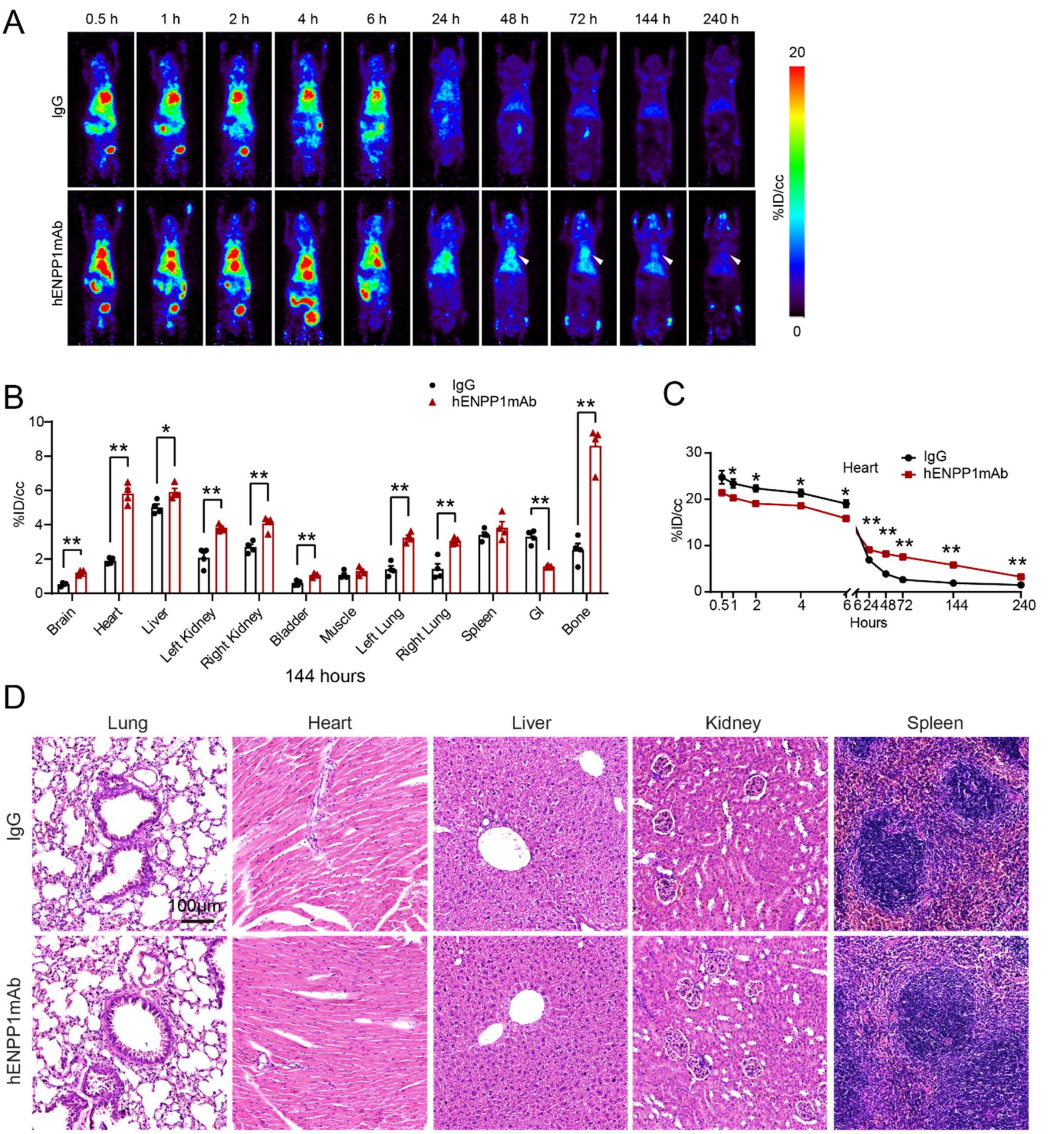

Fig S8. Organ wide biodistribution of radio-labeled hENPP1mAb after single dose administration in humanized ENPP1 animals. Related to Figure 2. (A) Representative longitudinal microPET imaging at 0.5, 1, 2, 4, 6, 24, 48, 72, 144, and 240 hours after Zr-IgG or 89Zr-hENPP1mAb injection in humanized ENPP1 mice. Arrowhead indicates the abundant 89Zr-hENPP1mAb signal in the heart at 48, 72, 144 and 240 hours (n=4 animals/group). **(B)** In vivo biodistribution of 89Zr-IgG or 89Zr-hENPP1mAb in brain, heart, liver, kidney, bladder, muscle, lung, spleen, gastrointestinal system and bone at 144 hours after injection (n=4 animals/group). **(C)** Time–activity (%ID/cc) curves in the hearts of 89Zr-IgG or 89Zr-hENPP1mAb treated animals. **(D)** Hematoxylin/eosin staining demonstrating normal histology of lung, heart, liver, kidney and spleen in both IgG (n=14) and hENPP1mAb injected animals (n=13). Data are represented as mean±SEM. **p<0.01, *p<0.05, Statistical significance was determined using Student's t-test, 2 tailed.

Fig. S9

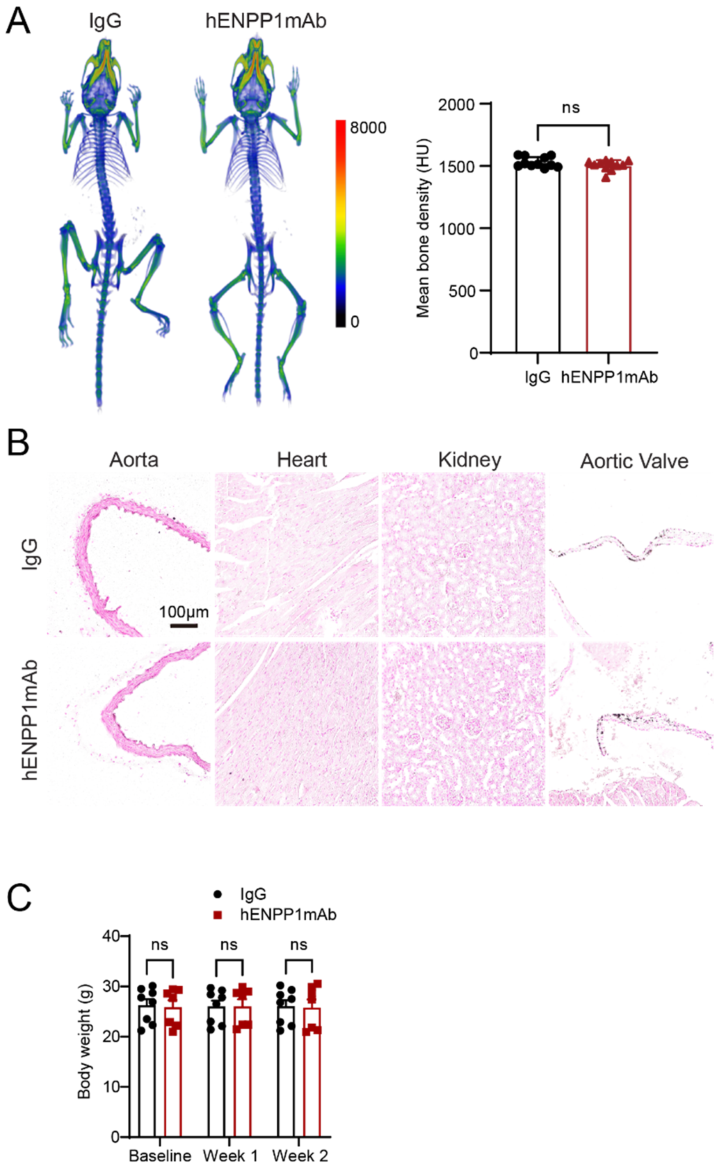

Fig S9. hENPP1mAb does not cause decreased bone mass or ectopic calcification. Related to Figure 2. (A) 3D rendering of bone density in IgG and hENPP1mAb treated humanized ENPP1 mice, 2 weeks after completion of 2 weeks of hENPP1mAb (i.e. 4 weeks after initial dose). Quantitative estimation of bone density (n=11 animals/group). The mean bone density (HU, Hounsfield unit) was calculated based on x-ray attenuation on microCT. **(B)** Von Kossa staining demonstrating absence of calcium deposits in aorta, heart, kidney and aortic valve in both IgG (n=14) and hENPP1mAb (n=13) treated mice. Organs were harvested at 4 weeks (i.e. 2 weeks after completion of 2 week treatment with hENPP1mAb). **(C)** Body weight at baseline, week 1 and week 2 following IgG control or hENPP1mAb treatment (n=8 in IgG and n=7 in hENPP1mAb). Data are represented as mean±SEM. ns: not significant, Statistical significance was determined using Student's t-test, 2 tailed.

Fig. S10

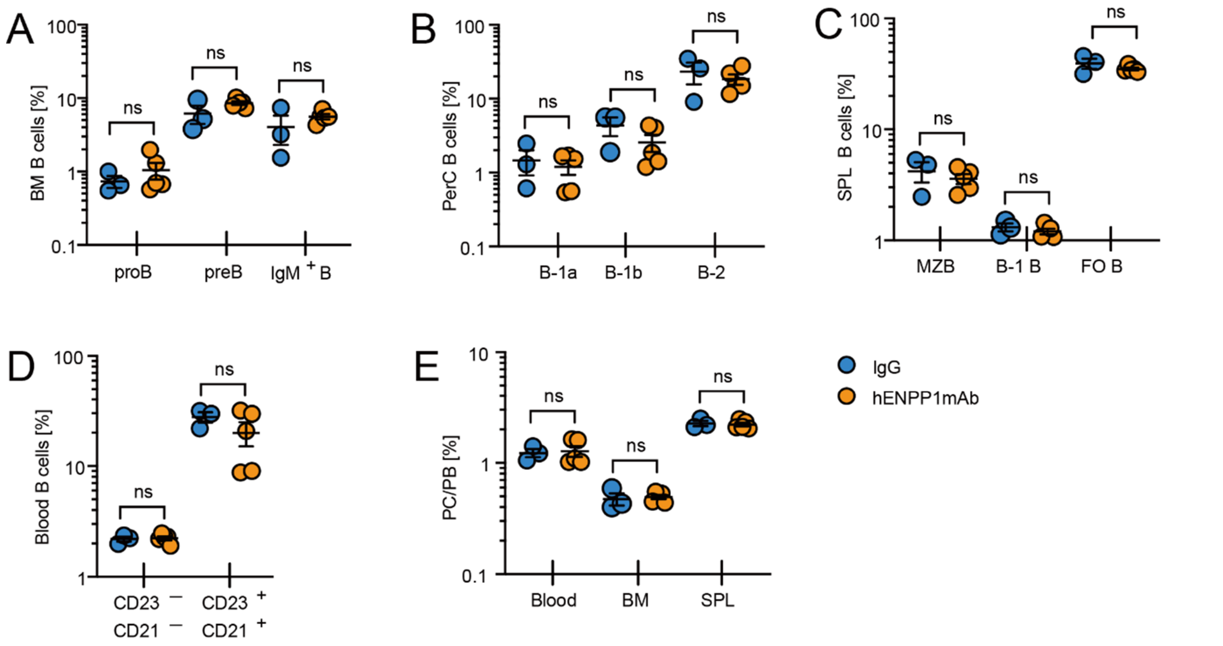

Fig S10. B cell populations between IgG treated and hENPP1mAb treated mice at 7 days following injection. Related to Figure 2. (A) Frequencies of B lineage cells in the bone marrow (BM) (proB, preB and IgM+ B cells). **(B)** Frequencies of B-1a, B-1b and B-2 cells in the peritoneal cavity (PerC). **(C)** Frequencies of Marginal Zone (MZB), B-1 and Follicular (FO) B-2 cells in the spleen (SPL). **(D)** Frequencies of CD23— CD21— IgM+ and CD23+ CD21+ IgM+ B cells in peripheral blood (Blood) and **(E)** Frequencies of plasma cells/plasma blasts (PC/PB) in peripheral blood (Blood), bone marrow (BM) and spleen (SPL). n=3 in IgG and n=5 in hENPP1mAb. Data represented as mean ± S.E.M., ns: not significant, Statistical significance was determined using Unpaired multiple t-test.

Table S1. Contingency table demonstrating number of animals with mild, moderate or severe impairment of ejection fraction at 4 weeks post MI, following treatment with hENPP1mAb versus IgG. Related to Figure 3.

	<20%	20%-40%	>40%	Total
IgG	11	10	0	21
hENPP1mAb	1	8	10	19

χ^2 =18.50, p value < 0.0001

Table S2. Contingency table demonstrating number of animals with mild, moderate or severe fibrosis at 4 weeks following MI in hENPP1mAb versus IgG treated groups. Related to Figure 4.

	<20%	20%-40%	>40%	Total
IgG	1	5	9	15
hENPP1mAb	7	12	3	22

χ^2 =9.39, p value =0.0091

Table S3. Serum biochemistry, complete blood counts (CBC) and differential blood counts in IgG and hENPP1mAb treated animals. Related to Figure 2. ($p>0.05$ for all values listed)

Biochemistry	IgG (n=14)	hENPP1mAb (n=13)
ALP (U/L)	86.14±29.046	83.77±26.671
AST (U/L)	280.5±218.169	387.46±206.731
ALT (U/L)	70.14±63.043	71.54±41.780
Albumin (g/dL)	3.03±0.338	3.09±0.161
Total Protein (g/dL)	4.89±0.475	5.05±0.161
BUN (mg/dL)	21.79±4.042	22.54±1.506
Creatinine (mg/dL)	0.10±0.104	0.11±0.104
Glucose (mg/dL)	209.79±55.445	217.85±27.850
Calcium (mg/dL)	8.59±0.818	8.43±0.523
Phosphorus (mg/dL)	8.21±1.959	8.48±0.939

CBC Parameters	IgG (n=14)	hENPP1mAb (n=13)
WBC (K/uL)	2.89±1.585	4.42±3.453
RBC (M/uL)	9.69±0.739	9.57±0.693
HGB (g/dL)	14.03±0.731	14.01±1.074
HCT (%)	44.87±3.334	44.5±3.062
MCV (fL)	46.29±0.994	46.46±1.127
Red Cell Distribution Width (%)	20.94±1.241	20.45±1.053
MCH (pg)	14.5±0.586	14.63±0.275
MCHC (g/dL)	31.31±1.275	31.47±0.705
Platelet Count (K/uL)	513.79±349.156	464.92±405.397

.

Differential (%)	IgG (n=14)	hENPP1mAb (n=13)
Neutrophil	10.78±5.353	9.12±5.937
Band (%)	none seen	none seen
Lymphocytes	81.11±5.945	82.36±7.209
Monocytes	4.6±2.134	6.23±3.164
Eosinpphils	3.24±2.632	2.17±1.770
Basophils	0.26±0.377	0.12±0.286
Metamyelocyte	none seen	none seen
Melocyte	none seen	none seen
Promyelocyte	none seen	none seen
Unclassified	none seen	none seen

Table S4. Serum biochemistry, CBC and Differential blood counts after 3 days of IgG and hENPP1mAb treatment. Related to Figure 2. ($p>0.05$ for all values listed)

Biochemistry	IgG (n=3)	hENPP1mAb (n=3)
ALP (U/L)	112.67±15.044	135.67±2.082
AST (U/L)	1041.50±919.946	828.67±250.698
ALT (U/L)	260.67±200.884	165.33±110.744
Albumin (g/dL)	2.97±0.231	2.93±0.321
Total Protein (g/dL)	4.77±0.416	4.73±0.586
BUN (mg/dL)	30.00±3.000	28.67±3.215
Creatinine (mg/dL)	0.10±0.100	0.10±0.100
Glucose (mg/dL)	242.00±20.664	245.00±27.875
Calcium (mg/dL)	8.47±2.146	9.00±0.265
Phosphorus (mg/dL)	10.17±0.987	9.37±2.281

CBC Parameters	IgG (n=3)	hENPP1mAb (n=3)
WBC (K/uL)	5.17±2.212	4.73±0.473
RBC (M/uL)	9.34±0.598	9.03±1.512
HGB (g/dL)	13.77±0.777	13.87±1.060
HCT (%)	42.47±3.584	39.70±8.390
MCV (fL)	45.67±1.528	44.00±2.646
Red Cell Distribution Width (%)	23.07±4.652	24.07±4.990
MCH (pg)	14.77±0.635	15.57±2.369
MCHC (g/dL)	32.53±2.747	35.97±7.941
Platelet Count (K/uL)	546.00±540.381	366.00±291.484

.

Differential (%)	IgG (n=3)	hENPP1mAb (n=3)
Neutrophil	7.83±4.537	9.77±8.177
Band (%)	0	0
Lymphocytes	87.33±6.658	84.77±11.927
Monocytes	4.07±2.101	4.10±2.152
Eosinophils	0.77±1.328	1.10±1.905
Basophils	0	0.27±0.462
Metamyelocyte	0	0
Melocyte	0	0
Promyelocyte	0	0
Unclassified	0	0

Body weight (g)	IgG (n=3)	hENPP1mAb (n=3)
Day 0	22.37±0.72	21.77±0.37
Day 3	21.43±0.39	21.83±0.47

Table S5. Elimination phase pharmacokinetic parameters of IgG and hENPP1mAb in Tg32 mice. Related to Figure 7.

	Half-Life (days)	Clearance (mL/days)	AUC (µg.days/mL)	Volume of Distribution (mL)
**IgG**	14.0±0.7	0.142±0.017	1274±161	2.85±0.32
**hENPP1mAb**	14.7±0.8	0.138±0.009	1210±36	2.93±0.24

Table S7. Sequences of cloning primers used. Related to STAR Methods.

	Forward Primer	Reverse Primer
human ENPP1	GCGGCCGCTGAGTTAACTATTCTAGAA CCATGGACGTGGGGGAGGAGCCGCT	GGGGGGGGAGGGAGAGGGGCGGAT CCTCAGTCTTCTTGGCTAAAGG
pig ENPP1	CGCTGAGTTAACTATTCTAGAACCATG GAGCGCGACGGCTGTG	GGGGAGGGAGAGGGGCGGATCCTC AGTCTTCTTGGTTAAAG
rat ENPP1	CGCTGAGTTAACTATTCTAGAACCATG GAGCGCGACGGCGAACA	GGGGAGGGAGAGGGGCGGATCCTC AGTCTTCTTGGCTGAAGAT
mouse ENPP1	GCGGCCGCTGAGTTAACTATTCTAGAA CCATGGAGCGCGACGGCGA	GGGGGGGGAGGGAGAGGGGCGGAT CCCTAGTCTTCTTGGCTGAAGATTG
monkey ENPP1 1st round upstream	ACGATGGAGCGCGACGGCT	AGGCAGCATCACAGCGACAGTTCC
monkey ENPP1 1st round downstream	ATGGACGTGGGGGAGGAGC	GCTGAGTACGCAAGTTTCCCTGAGAT
monkey ENPP1 2nd round upstream	TATTCTAGAACCATGGAGCGCGACGGC TG	AGGCAGCATCACAGCGACAGTTCC
monkey ENPP1 2nd round downstream	TATATTCCATGGACGTGGGGGAGGAG C	CTAGGATCCTCAGTCTTCTTGGCTAA AGGTTGGCAAATGTG
human ENPP4	TATGCTAGCCACCATGAAGTTATTAGTA ATACTTTTGTTTTCTGG	ATCCCGGGTCACCCAATTAAAGGATC ATC
human ENPP5	TCCGCTAGCCACCATGACTTCGAAATT TCTCTTGGTG	ATCCCGGGTTAGGCTTGTAATAATGG TTGAGCT
human CD39	TCCGCTAGCCACCATGGAAAGTGAAGA GTTGGCA	ATCCCGGGCTATACCATATCTTTCCA GAAATATGAAG
human CD73	TATGCTAGCCACCATGTGTCCCCGAGC CG	ATCCCGGGCTATTGGTATAAAACAAA GATCACTGC
ENPP5-GFP	CCGGTCGCCACCATGGTGAGCAAGGG	CATGGTGGCGACCGGGGCTTGTAAT AATGGTTGAGCT

Table S8. Sequences of ECM genes primers used. Related to STAR Methods.

	Forward primer	Reverse primer
Col1a1	TTCTCCTGGCAAAGACGGAC	CGGCCACCATCTTGAGACTT
Col1a2	CCCAGAGTGGAACAGCGATT	ATGAGTTCTTCGCTGGGGTG
Acta2	GTACCACCATGTACCCAGGC	GCTGGAAGGTAGACAGCGAA
Fn1	ATGAGAAGCCTGGATCCCCT	GAGAGCTTCCTGTCCTGTCT
Postn	TCCCTGATGTCATTACTGATCC	AACCATCTTCAGCCCTGAGC

Table S9. In vivo micro-CT imaging Parameters. Related to STAR Methods.

Acquisition Parameters	
x-ray tube voltage:	50 kVp
x-ray tube current:	200 μA
Exposure time:	20 ms
Number of projections:	21600
Scan duration:	12 mins
Capture rate:	1751 projections/min.
Camera pixels:	972 x 768
Pixel size:	149.6 μm

Reconstruction Parameters	
Method:	Iterative reconstruction (OSEM)
Number of subsets:	16
Number of iterations:	6
Voxel size:	0.108 mm isotropic
Volumetric image size:	380 x 380 x 200 voxels (thorax only)
Post-reconstruction filter:	Gaussian (spatial), Median (temporal)
